# The cancellation effect at the group level

**DOI:** 10.1111/evo.13995

**Published:** 2020-05-21

**Authors:** Aslıhan Akdeniz, Matthijs van Veelen

**Affiliations:** ^1^ Department of Economics and Business University of Amsterdam 1012 WX Amsterdam The Netherlands; ^2^ Tinbergen Institute 1082 MS Amsterdam The Netherlands

**Keywords:** Cancellation effect, evolution of cooperation, group selection

## Abstract

Group selection models combine selection pressure at the individual level with selection pressure at the group level. Cooperation can be costly for individuals, but beneficial for the group, and therefore, if individuals are sufficiently much assorted, and cooperators find themselves in groups with disproportionately many other cooperators, cooperation can evolve. The existing literature on group selection generally assumes that competition between groups takes place in a well‐mixed population of groups, where any group competes with any other group equally intensely. Competition between groups however might very well occur locally; groups may compete more intensely with nearby than with far‐away groups. We show that if competition between groups is indeed local, then the evolution of cooperation can be hindered significantly by the fact that groups with many cooperators will mostly compete against neighboring groups that are also highly cooperative, and therefore harder to outcompete. The existing empirical method for determining how conducive a group structured population is to the evolution of cooperation also implicitly assumes global between‐group competition, and therefore gives (possibly very) biased estimates.

There is a wide variety of positions on the role of group selection in human evolution. One end of the spectrum considers group selection to be a key ingredient of human evolution (Sober and Wilson 1998; Wilson and Wilson 2007; Haidt 2012; Richerson et al. 2016). The other side suggests that “group selection has no useful role to play in psychology or social science” (Pinker 2015); see also Williams 1966 and Wade 1978. In this article we will not resolve this controversy, nor take a position in this debate, but what we will do is consider a crucial element that has been missing, both from the current group selection models, and from the current empirical approach to establishing how conducive group structure is to the evolution of cooperation.

The defining characteristic of a group selection model is that it captures the opposing effects of selection at the individual level, where defectors do better than cooperators within groups, and selection at the group level, where groups with more cooperators do better than groups with fewer cooperators (Wilson and Wilson 2007). The existing models within the group selection literature all share the property that competition between groups happens globally; all groups compete with all other groups equally intensely (Traulsen and Nowak 2006; Boyd and Richerson 2009; Simon 2010; Simon et al. 2013; Luo 2014; van Veelen et al. 2014; Luo and Mattingly 2017). This is a useful simplification if the aim is to illustrate the possibility of a tug of war between the different levels of selection. It may however not always be particularly realistic. Groups themselves typically live in a structured population of groups, where neighboring groups compete with each other more than they do with groups that are further away. Local dispersal would then imply that groups with many cooperators are typically surrounded by groups that also contain many cooperators, compared to the groups that surround groups with many defectors. More cooperative groups therefore might also be subject to more intense competition at the group level. This can significantly dampen the benefits of being a cooperative group, which, in turn, affects the balance between selection at the individual and at the group level. In models without group structure a similar phenomenon, but then at the individual level, is called the cancellation effect (Wilson et al. 1992; Taylor 1992a, b). We show that the cancellation effect also exists at the group level, where it plays out in a more complex way, and that it can make a sizable difference for the conditions under which cooperation can evolve by group selection. This also has empirical implications. The current standard approach to determining how large the benefit to the group should be, compared to the cost to the individual, for cooperation to evolve by group selection implicitly assumes global between‐group competition (Aoki and Nozawa 1984; Crow and Aoki 1984; Weir and Cockerham 1984; Bowles 2006; Bell et al. 2009; Langergraber et al. 2011; Walker 2014). If competition between groups is not global, but at least to some extent local, then this procedure paints too positive a picture of how favorable conditions are for the evolution of cooperation by group selection.

## Model

**Figure 1:**
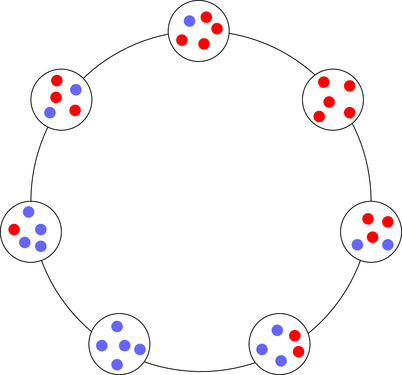
An example of a population state on a cycle with m=7 groups of n=5 individuals each. The blue dots indicate cooperators and the red dots indicate defectors.

To study the difference between global and local group competition, we consider a stylized model, in which m groups consisting of n individuals live on a cycle (Fig. 1). Individuals can either be a cooperator (C) or a defector (D). In every time period, one of three types of events will happen: individual reproduction, group reproduction, or migration. These events happen with probabilities p, q, and r, respectively, where p+q+r=1. We compare two different processes for group reproduction, one with local and one with global between‐group competition.

If an individual reproduction event occurs, first a random group is selected, where all groups have equal probability of being chosen. Then an individual from the selected group is chosen to reproduce. Within the group, defectors get a payoff of 1 and cooperators get a payoff of 1−c. The intensity of selection w is then used to transform these payoffs to values $f_{C}$ and $f_{D}$:

$$\begin{array}{c} f_{C}=1-wc\;\text{and}\;f_{D}=1. \end{array}$$

**Figure 2:**
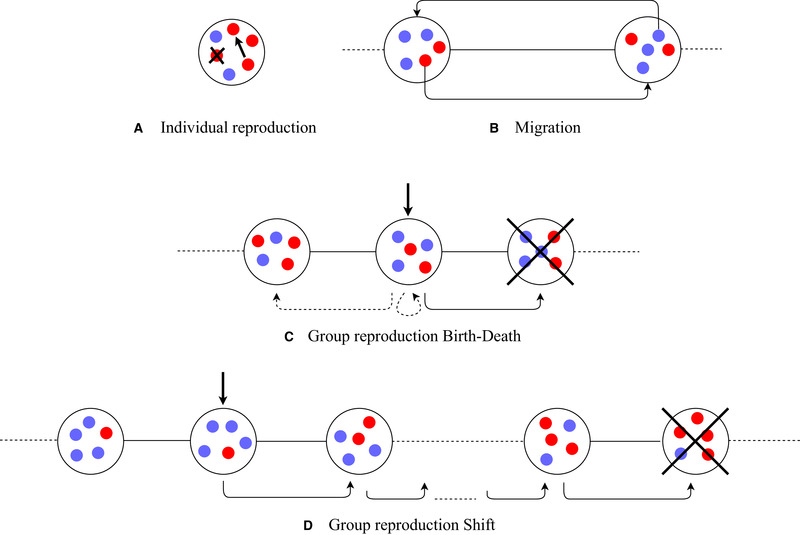
(A) At an individual reproduction event, one individual reproduces, and one individual within the same group dies. In this example, one defector is chosen for reproduction, and another defector is chosen to die, so the overall group composition has not changed. Defectors have a higher chance of being chosen for individual reproduction than cooperators do. (B) At a migration event, two individuals from neigboring groups trade places. (C) In the Birth‐Death process, the group that is chosen to reproduce produces an identical offspring group. This offspring group then replaces one of the neighboring groups, or, with a small probability, it replaces the parent group itself. (D) In the Shift process, the group that is chosen to reproduce also produces an identical offspring group, but here any group can be chosen to die, including the parent group. If the parent group and the dying group are more than one position apart, all groups between them move over one position. In both processes, groups with many cooperators have a higher chance of being chosen for group reproduction than groups with many defectors.

The probabilities with which individuals are chosen for reproduction within the group are proportional to these values. Whenever an individual reproduces, an individual from the same group is chosen to die, where each individual, including the parent, but excluding the offspring, is chosen with probability $\frac{1}{n}$ (Fig. 2A).

If a group reproduction event occurs, then one group is chosen to reproduce, and one group is chosen to die. The groups are numbered i=1,…,m, and $k_{i}$ is the number of cooperators in group i. These groups live on a cycle, so i and i+1 are neighboring groups, and so are groups 1 and m. The group payoff of group i is 1 plus b times the share of cooperators in the group. The intensity of selection w is then used to transform these payoffs to values

$$\begin{array}{c} g\left(k_{i}\right)=1+w\frac{k_{i}}{n}b. \end{array}$$

We consider two update processes for group reproduction; Birth‐Death and Shift. In both of them, first a group is chosen for reproduction, where each group's probability of being chosen is proportional to their value $g(k_{i})$. With Birth‐Death, the offspring group then replaces the left or the right neighbor of the parent group, both with probability $\frac{m-1}{2m}$, and it replaces its own parent group with probability $\frac{1}{m}$ (Fig. 2C). This makes competition at the group level local. With Shift, each group, including the parent group, but excluding the offspring group, is chosen to die with probability $\frac{1}{m}$. Unless the offspring group replaces the parent group, the new group is placed either to the right or to the left of the parent group, with equal probability, and every other group in between the parent group and the dying group moves over one spot (Fig. 2D). With Shift, every group is equally likely to die, irrespective of the composition of their neighboring groups. Competition between groups is therefore global, as it is in the standard group selection models that have a well‐mixed population of groups.

Finally, if a migration event happens, then a random pair of individuals from neighboring groups trade places (Fig. 2B).

## Results

We first analyze this model in the limit of weak selection using inclusive fitness. We can do this, because the effects that being a cooperator instead of a defector has on individual reproduction rates, and on individuals death rates, as well as the effects it has on reproduction and death rates of groups, satisfy generalized equal gains from switching in the limit of weak selection (van Veelen et al. 2017, van Veelen 2018).

**Figure 3:**
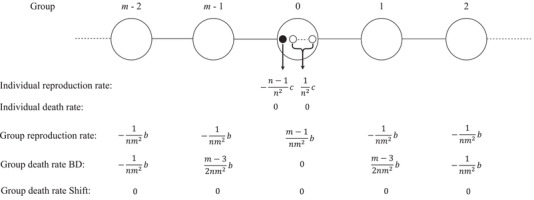
An overview of all the fitness effects in the limit of weak selection, conditional on an individual event happening in the group of the focal individual, or a group event happening, respectively. The black dot represents the focal individual.

The fitness effects of the focal individual being a cooperator instead of a defector are given in Figure 3. Conditional on an individual event happening in the group of the focal individual, the probability that any given individual is chosen to reproduce is proportional to its payoffs, scaled by the intensity of selection. That implies that these probabilities are the individual's own value, which is either $f_{C}$ or $f_{D}$, over the sum of these values for everyone within the same group, including the individual itself. In the limit of weak selection, that amounts to a decrease proportional to $\frac{1}{n}c$ for the focal individual due to the decrease in the numerator of this probability, and an increase proportional to $\frac{1}{n^{2}}c$ for everyone, including the focal individual, due to the decrease in the denominators. Those add up to the changes in individual reproduction rates given in Figure 3. Individual death rates are unaffected.

Conditional on a group event happening, the probability that group i is chosen to reproduce is proportional to $g(k_{i})$, which is the average level of cooperation within the group, scaled by the intensity of selection. The effect of being a cooperator instead of a defector on the group average $k_{i}$ is $\frac{1}{n}b$, and in the limit of weak selection, the effect on the group reproduction probability is proportional to $\frac{1}{m}\frac{1}{n}b$ through an increase in the numerator for the group of the focal individual, and $-\frac{1}{m^{2}}\frac{1}{n}b$ through an increase in the denominator for every group, including the group of the focal individual. Those amount to the changes in group reproduction rates given in Figure 3.

For Birth‐Death, an increase in the reproduction rate of the group that the focal individual is in increases the death rates of the two neighboring groups, and reduces the death rates of all other groups. For Shift, all groups have a probability $\frac{1}{m}$ of dying, so changes in group reproduction rates do not affect any group's death rate.

In Section 3 of the Supporting Information, we derive and discuss these effects in detail. In the limit of w↓0, we also add them up, weighted by the relatedness of the individuals affected. The relatedness between two individuals whose groups are i steps apart is defined as the low mutation limit of

$$r_{i}=\frac{q_{i}-\bar{q}}{1-\bar{q}},$$

where $q_{i}$ denotes the stationary identical‐by‐descent probability for the two individuals, and $\bar{q}$ denotes the average identical‐by‐descent probability of a focal individual to all the individuals in the population, including the focal individual itself (see Section 4 in the Supporting Information for further details). This implies that these relatednesses are relative measures, which are positive for individuals in close by groups and negative for individuals in far‐away groups, and that they sum up to 0.

For the Birth‐Death process we then find that cooperators are selected for if

(1) $$-p\frac{1}{m}\left(1-r_{0}\right)\frac{1}{n}c+qn\left(r_{0}-\left(\frac{1}{m}r_{0}+\frac{m-1}{m}r_{1}\right)\right)\frac{1}{nm}b>0.$$

The first term reflects all changes in individual reproduction rates. The probability that an individual event happens is p. The probability that if it does, it happens in the group of the focal individual is $\frac{1}{m}$. If we write the effect on the individual reproduction rate of the focal individual as $-\frac{1}{n}c+\frac{1}{n^{2}}c$, then we can also see the individual effects as a combination of a reduction in individual reproduction rate of the focal individual by $\frac{1}{n}c$, and an increase in individual reproduction rate of $\frac{1}{n^{2}}c$ for everyone in the group, including the focal individual. With n individuals per group, the latter is equivalent to an effect of $\frac{1}{n}c$ on a randomly chosen individual from the same group, including the focal individual. This randomly chosen individual is related $r_{0}$ to the focal individual.

The term $qnr_{0}\frac{1}{nm}b$ reflects the effects through changes in group reproduction rates. The probability that a group event happens is q, and if it does, all n individuals in the group reproduce. If we write the effect on the group reproduction rate of the focal individual as $\frac{1}{nm}b-\frac{1}{nm^{2}}b$, then we can also see the group effects as a combination of an increase in reproduction rate of the group the focal individual is in by $\frac{1}{nm}b$, and a decrease in reproduction rate of $\frac{1}{nm^{2}}b$ for all groups, including the group the focal individual is in. The latter is equivalent to an effect of $\frac{1}{nm}b$ on a randomly chosen group, including the group the focal individual is in. A randomly chosen individual from a randomly chosen group is related $\sum_{i=0}^{m-1}r_{i}=0$ to the focal individual.

The term $-qn\left(\frac{1}{m}r_{0}+\frac{m-1}{m}r_{1}\right)\frac{1}{nm}b$ reflects the effects through changes in group death rates. This matches the group replacement rule for Birth‐Death, where a reproducing group replaces itself with probability $\frac{1}{m}$, and one of its neighboring groups with probability $\frac{m-1}{m}$. A randomly chosen individual from the neighboring groups is related $r_{1}$ to the focal individual.

For Shift, almost everything is the same, and the only thing that is different is that all group death rates are unaffected. That makes the counterpart of Condition (1) simpler.

(2) $$-p\frac{1}{m}\left(1-r_{0}\right)\frac{1}{n}c+qnr_{0}\frac{1}{nm}b>0.$$

There are two differences between these two conditions. The first is that $r_{0}$ will not be the same between the two processes, even if everything else (i.e., p, q, r, n, and m) is equal. In the Supporting Information, we calculate how $r_{0}$ depends on those five parameters for both processes, and it turns out that $r_{0}$ tends to be higher for Birth‐Death than for Shift. Therefore, if this was the only difference, it would actually be easier to evolve cooperation in Birth‐Death than it would be for Shift. The second difference is that Condition (1) has a $-(\frac{1}{m}r_{0}+\frac{m-1}{m}r_{1})$ term that is absent in Condition (2). This term reflects the cancellation effect, and it makes the evolution of cooperation harder.

We can rewrite both inequalities as conditions on the b/c ratio. Condition (1) for Birth‐Death then becomes

(3) $$\begin{array}{c} \frac{b}{c}>\frac{p}{q}\frac{1-r_{0}}{n\left(r_{0}-\left(\frac{1}{m}r_{0}+\frac{m-1}{m}r_{1}\right)\right)}. \end{array}$$

Condition (2) for Shift becomes

(4) $$\begin{array}{c} \frac{b}{c}>\frac{p}{q}\frac{1-r_{0}}{nr_{0}}. \end{array}$$

One can also consider a more general class of processes that are the same as Birth‐Death and Shift with respect to their individual reproduction, but that vary in how local between‐group competition is. For simplicity, we can assume for all processes that if a group is chosen to reproduce, then the parent group itself is chosen to die with probability $\varphi_{0}=\frac{1}{m}$. For the remainder of the probabilities $\varphi_{i},i=1,\ldots ,m-1$, we only assume symmetry ($\varphi_{j}=\varphi_{m-j}$) and, because they are probabilities, $\sum_{i=0}^{m-1}\varphi_{i}=1$. Groups between the reproducing group and the dying group then move over in the same way as they do in Shift. If we do, we find a more general condition that encompasses Conditions (3) and (4):

(5) $$\begin{array}{c} \frac{b}{c}>\frac{p}{q}\frac{1-r_{0}}{n\left(r_{0}-\sum_{i=0}^{m-1}\varphi_{i}r_{i}\right)}. \end{array}$$

Birth‐Death is a special case of this larger collection of models with $\varphi_{0}=\frac{1}{m}$, $\varphi_{1}=\varphi_{m-1}=\frac{m-1}{2m}$, and $\varphi_{i}=0$ for i=2,…,m−2. To get Condition (3) for Birth‐Death, we use $r_{1}=r_{m-1}$. Shift is a special case with $\varphi_{i}=\frac{1}{m}$ for all i, and to get Condition (4), we use $\sum_{i=0}^{m-1}r_{i}=0$. It should be noted that the relatednesses in Condition (5) are still endogenous; they depend on the process we choose.

**Figure 4:**
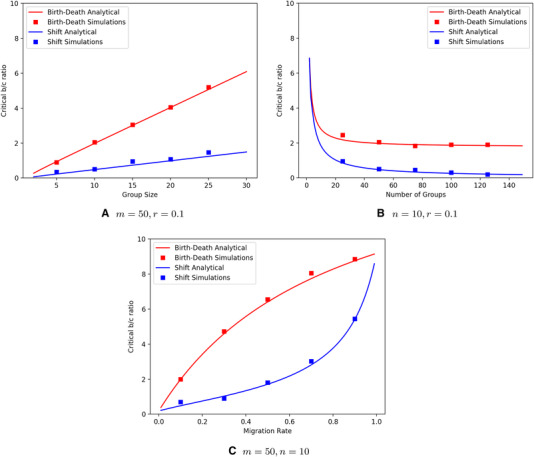
Critical b/c ratios in the limit of weak selection for Birth‐Death (red lines) and Shift (blue lines), as well as simulation results at an intensity of selection w=0.1, for Birth‐Death (red squares) and Shift (blue squares). In panels (A) and (B), one in every 10 events is a migration event (r=0.1). In panels (A) and (C), the number of groups is set to m=50. In panels (B) and (C), the group size is set to n=10. Probabilities p and q are chosen so that the average individual is as likely to die as a result of an individual reproduction event as it is to die from a group reproduction event under neutral selection: $p=\left(1-r\right)\frac{n}{n+1}$ and $q=\left(1-r\right)\frac{1}{n+1}$. Similar to a model without population structure at the group level (Traulsen and Nowak 2006), larger group sizes (A) and larger migration rates (C) increase the critical b/c ratio, and larger numbers of groups (B) decrease it. For m→∞, the threshold for Shift goes to 0, because $r_{0}$ then converges to 1. The gap between Birth‐Death and Shift is there for a range of group sizes, numbers of groups, and migration rates. The gap between the two processes disappears when the migration rate vanishes, in which case the dynamics are such that all groups are at within‐group fixation almost all of the time (see Sections 2 and 8 of the Supporting Information for why that makes the gap disappear). The gap also disappears when the migration rate is close to 1, and the whole population is shaken and stirred between any two reproduction events. Section 7 of the Supporting Information gives the complete argument why relatednesses $r_{0}$ and $r_{1}$ being close to 0 not only means that the right‐hand sides of Conditions (3) and (4) should be similar, but that they actually are exactly the same in the limit of r→1.

In Section 4 of the Supporting Information, we derive analytical expressions for relatednesses for Birth‐Death and Shift by extending the method from Grafen (2007) to group structured populations. That gives us relatednesses at neutrality, which is appropriate in the case of weak selection. Figure 4 shows how the critical b/c ratios in Conditions (3) and (4) depend on the group size, the number of groups, and the migration rate, if we fill in those relatednesses.

Once we move away from the limit of weak selection, the model quickly becomes intractable. To study the model not in the limit of weak selection, we ran simulations. The critical b/c ratios we find for an intensity of selection of w=0.1 are also shown in Figure 4. For intensity of selection w=0.5 they can be found in the Supporting Information. These simulations show a similar gap between Birth‐Death and Shift, and they suggest that the analytical results in the limit of weak selection are quite informative here (Wu et al. 2013). Section 5 in the Supporting Information also contains a mathematical proof that the threshold for Birth‐Death is always higher than the one for Shift as soon as the number of groups exceeds 3. For m=2 or m=3, the two different update processes imply the same dynamic.

## Relation to Other Models

We chose our model to make it as simple as possible to illustrate the difference between global and local between‐group competition. We expect that the cancellation effect at the group level will show up in all models with local between‐group competition. Other models that allow the scale of between‐group competition to vary may however not allow for such relatively straightforward comparisons. We will go over a few other models that one could also combine with ways to model local between‐group competition.

In Luo (2014) and van Veelen et al. (2014), the rate at which individuals reproduce is an individual characteristic, which is independent of the population state. It can either be high or low, depending on whether an individual is a defector or a cooperator. Also the reproduction rate of a group only depends on the number of cooperators in the group itself; it will be high if there are many, and low if there are few. This implies that the ratio of group events to individual events depends on the population state; if everyone is a cooperator, this ratio will be lower than if everyone is a defector. In our model, the probabilities p and q are fixed, and being a cooperator only has an effect on the individual reproduction rate, conditional on the group it is in being chosen to host an individual event. This is less realistic, and perhaps also less elegant, but it does make it easier to capture the effect of local between‐group competition in relatively concise formulas.

In Traulsen and Nowak (2006), individual reproduction events make groups grow bigger, and when they reach maximum capacity, occasionally an individual reproduction event does not lead to another individual within the group dying, but to the group splitting into two daughter groups. Cooperators reduce their own reproduction rate but increase the reproduction rate of others in their group. Individuals in groups with many cooperators reproduce more often, and therefore they also make their groups split more often. In our model, group reproduction events are not triggered by individual reproduction events.

There are also differences in the methods used to derive analytical solutions. Traulsen and Nowak (2006) assume a separation of timescales by considering the case where the probability that a group splits as a result of an individual reproduction event is vanishingly small. That results in a nested Moran process, for which they compute fixation probabilities in the limit of weak selection. That is different from our analysis. What is somewhat similar, is that the group reproduction stage ends up being condensed in both. In their case, it is the result of the separation of timescales. We simply assume that a group as a whole reproduces in one go, thereby bundling a sequence of individual reproduction events and a splitting event together. That is, again, not particularly realistic or elegant, but it does help avoid having to make other, perhaps more consequential unrealistic assumptions to be able to derive analytical solutions. Also, in Section 8.2 of the Supporting Information, we do take a somewhat similar approach by considering the limit of p→1, but without assuming selection to be weak. There, we find that the difference between Birth‐Death and Shift disappears with the separation of timescales. The reason why it does is similar to the reason why it dissipates without migration, when the dynamics also make groups be at within‐group fixation almost all of the time.

To find analytical solutions, Luo (2014), van Veelen et al. (2014), Simon (2010), and Simon et al. (2013) all assume a dynamic equilibrium, where every individual group will keep changing composition, but in the equilibrium distribution of group types in the population as a whole, these changes balance. They moreover consider a limit where of both the number of groups and the group size approach infinity. It may be possible to create a version of their model, where groups are situated on the cycle as well, but their approach to deriving analytical solutions would not generalize in a straightforward way.

Our model, where groups replace other groups, would fall under Multilevel Selection 2 in the classification of Okasha (2006), under “old group selection” in terms of West et al. (2007), or “replacement group selection” in terms of Molleman et al. (2013). This is not necessarily an unrealistic possibility; see Soltis et al. (1995). If, instead of replacing other groups more often, successful groups produce more offspring, which then migrate to other groups, then that would classify as Multi‐Level Selection 1, “new group selection,” or “contagion group selection.” Such a model would fit Rousset and Billiard (2000), who present a model with localized dispersal on a cycle, but without group level events. They do not interpret theirs as a group selection model, nor do they discuss the cancellation effect, but in their analysis, relatednesses with individuals in neighboring demes do play a similar role. Both their and our model can also be seen as examples of metapopulation models (Hanski 1998, 1999).

## Discussion and Implications for Empirical Studies

Our results show that in models of group selection, the evolution of cooperation can be quite a bit harder if between‐group competition is local instead of global. The difference in critical b/c ratios can be more than substantial between Birth‐Death, which has completely local between‐group competition, and Shift, for which between‐group competition is completely global. The particular structure we considered—the cycle—is obviously very simple, and not particularly realistic. It may represent some populations, if they are constrained by geographic characteristics, such as rivers, or chains of mountains. For example, Howell (1952) has noted that the Shilluk, a Nilotic tribe, are organized into divisions of settlements situated along the west bank of the Nile in a linear fashion. For this population, a one‐dimensional model is a good approximation. For most populations, however, the cycle is not a good model. We do nonetheless think that its simplicity allows us to demonstrate a more general effect, which we expect will also occur with more realistic and complex ways in which groups can be located in a higher dimensional spatial structure.

It is probably less unrealistic to assume that between‐group competition is at least to some extent local. Straightforward examples of local between‐group competition are warfare in the Enga society, which happens within the same ethnic group (Wiessner et al. 2010; Wiessner and Pupu 2012; Wiessner 2019), endemic warfare in the Asabano society in the precontact era (Lohmann 2014), or feuds among the Shilluk settlements mentioned above, which usually take place among direct neighbors (Howell 1952). Examples for which one can reasonably assume that between‐group competition is global, on the other hand, will be much harder to find.

This also has empirical implications. The current, well‐established approach in empirical studies concerning group selection is to measure $F_{ST}$'s—the empirical equivalent of $r_{0}$ in our model—to determine how large the benefit to the group should be, compared to the cost to the individual, for cooperation to evolve. The condition that Bell et al. (2009) uses for when cooperation will be selected for by group selection (see also Aoki and Nozawa 1984; Crow and Aoki 1984; Weir and Cockerham 1984; Bowles 2006, 2009; Langergraber et al. 2011; Walker 2014; Rusch 2018) is

$$\frac{\beta (w_{g},p_{g})}{\beta (w_{ig},p_{ig})}>\frac{1-F_{ST}}{F_{ST}}.$$

Here $\beta (w_{g},p_{g})$ is the increase in mean fitness of the group as a result of an increase in the frequency of cooperators, or altruists, and $\beta (w_{ig},p_{ig})$ is the decrease in fitness of an individual as a result of switching from defection to cooperation. The idea is that this criterion separates the fitness effects, on the left‐hand side of the inequality, from a measure that characterizes the population structure, on the right‐hand side of the inequality. In a setting where the fitness effects constitute a linear public goods game, played within groups that compete with each other globally, such a separation can indeed be made in this way (see Section 9 of the Supporting Information and van Veelen 2020). A small, collateral finding here is that in such a setting, one should compute the $F_{ST}$ without, and not with replacement, as is usually done.

If we were to measure $\beta (w_{g},p_{g})$ in a setting in which competition between groups is not actually global, but to some degree local, then the resulting value for $\beta (w_{g},p_{g})$ would not only reflect the effect of cooperators on the average fitness within the group, but a mixture of these fitness effects and the cancellation effect. A moderate value for $\beta (w_{g},p_{g})$ can both be the result of a moderate group benefit and the absence of the cancellation effect, and a high group benefit combined with the cancellation effect at the group level. In the latter case, the negative effect of having neighboring groups with many cooperators, combined with the positive correlation between being a cooperative group and having neighboring groups with many cooperators, would bias the estimated effect of—all else equal—the number of cooperators on average fitness within the group downward. In other words, this term would end up absorbing the cancellation effect. To disentangle all fitness effects and the cancellation effect, one would have to estimate a more complex statistical model, which would not only use the composition of the own group as an explanatory variable of the average fitness within the group, but also include the composition of neighboring groups as an explanatory variable. This would then have to be combined, not just with the relatedness within groups, but also with the relatedness with individuals in neighboring groups.

What most empirical papers do, however, is only estimate the $F_{ST}$, which is then taken as an indication of how conducive the population structure is to cooperation. The implicit assumption in that approach is that competition between groups is global. We have seen that the absence or presence of the cancellation effect—which is part of the population structure—can make a huge difference for how much the group needs to benefit from cooperators in it, relative to the individual costs, in order for cooperation to spread in the population. If competition between groups is not global, but at least to some extent local, then this procedure therefore paints a too positive picture of how favorable conditions are for the evolution of cooperation by group selection.

## CONFLICT OF INTEREST

The authors do not have a conflict of interest to declare.

Associate Editor: E. Kisdi

Handling Editor: M.R. Servedio

## Supporting information


**Figure 1**. An example of a population state on a cycle with *m* = 7 groups of *n* = 5 individuals each.
**Figure 2**. An example of the individual reproduction events.
**Figure 3**. The BD process.
**Figure 4**. The Shift process.
**Figure 5**. An example of a migration event, where a defector from the group on the left and a cooperator from the group on the right change places.
**Figure 6**. An overview of all the fitness effects in the limit of weak selection, conditional on an individual event happening in the group of the focal individual, or a group event happening, respectively.
**Figure 7**. An example of the last few steps in the iterative process of finding the critical *b*/*c*‐ratio.
**Figure 8**. Results for the critical *b*/*c* ratios, combined with simulations with 1,000,000 independent runs.
**Figure 9**. Same as Figure 8, but with *w* = 0.5.
**Figure 10**. Results for the critical *b*/*c* ratios for different migration rates *r*, for *n* = 10, *m* = 50, again combined with simulations with 1,000,000 independent runs.
