# Supplementary material for: The cancellation effect at the group level
Source: Evolution. 2020 May 21;74(7):1246–54. doi: 10.1111/evo.13995 (PMC7496855; doi:10.1111/evo.13995)
Supplement: Supplementary file 1 — Figure 1. An example of a population state on a cycle with m = 7 groups of n = 5 individuals each. Figure 2. An example of the individual reproduction events. Figure 3. The BD process. Figure 4. The Shift process. Figure 5. An example of a migration event, where a defector from the group on the left and a cooperator from the group on the right change places. Figure 6. An overview of all the fitness effects in the limit of weak selection, conditional on an individual event happening in the group of the focal individual, or a group event happening, respectively. Figure 7. An example of the last few steps in the iterative process of finding the critical b/c‐ratio. Figure 8. Results for the critical b/c ratios, combined with simulations with 1,000,000 independent runs. Figure 9. Same as Figure 8, but with w = 0.5. Figure 10. Results for the critical b/c ratios for different migration rates r, for n = 10, m = 50, again combined with simulations with 1,000,000 independent runs. [file EVO-74-1246-s001.pdf]

Supporting Information for: The cancellation effect at the group level by Aslıhan Akdeniz and Matthijs van Veelen

Contents

1 Introduction **3**

1.1 The cancellation effect at the individual level 3

1.2 The cancellation effect at the group level 3

2 The model **5**

2.1 Two update rules on the cycle 5

2.1.1 Individual events 5

2.1.2 Group events 6

2.1.3 Migration events 8

2.2 Alternative modeling choices 9

3 Analytical results in the limit of weak selection **12**

3.1 Changes in reproduction rates 12

3.1.1 Changes in individual reproduction rates 12

3.1.2 Changes in group reproduction rates 14

3.1.3 Overall effect through changes in reproduction rates 15

3.2 Changes in death rates 16

3.2.1 Changes in individual death rates 16

3.2.2 Changes in group death rates 16

3.2.3 Overall effect through changes in death rates 17

3.3 Overall effect of switching from D to C 18

3.3.1 Birth-Death 18

3.3.2 Shift 20

3.4 Comparing thresholds 21

3.5 Hamilton's rule 21

3.6 More general version and interpretation 21

3.6.1 Changes in group death rates 21

3.6.2 Overall effect of switching from D to C 22

4 Relatedness **24**

4.1 Birth-Death 24

4.1.1 $i = 1$ 25

4.1.2 $1 < i < m - 1$ 26

4.1.3 $i = 0$ 27

4.1.4 Solving the system 28

4.2 Shift 34

4.2.1 $i = 1$ 34

4.2.2 $1 < i < m - 1$ 36

4.2.3 $i = 0$ 37

4.2.4 Solving the system . . . 37
4.3 Three useful identities . . . 42
4.4 Alternative derivation of the three identities . . . 43
4.4.1 Within-group relatedness . . . 44
4.4.2 Relatedness with an individual from a random other group . . . 44
4.4.3 Relatedness between individuals that are i groups apart . . . 44
4.4.4 Identity I . . . 45
4.4.5 Identity II . . . 46
4.4.6 Identity III . . . 47

5 Birth-Death versus Shift **48**
5.1 When m is odd . . . 51
5.2 When m is even . . . 57
5.3 Limit results for the number of groups approaching infinity . . . 60
5.3.1 BD . . . 60
5.3.2 Shift . . . 60
5.3.3 Birth-Death versus Shift in the limit where number of groups approach infinity . . . 61

6 Simulations **62**

7 Theoretical results and simulations **65**

8 Analytical solutions, without migration, and for limit cases that are not the limit of weak selection **68**
8.1 Fixation probabilities of mutant groups . . . 68
8.2 The limit $p \to 1$ . . . 69
8.3 The limit $p \to 0$ . . . 71

9 Empirical implications **73**
9.1 F_{ST} . . . 73
9.2 Empirical estimates . . . 75

1 Introduction

1.1 The cancellation effect at the individual level

The cancellation effect at the individual level was discovered by Wilson et al. (1992) and Taylor (1992a;b). Before then, it was more or less generally thought that as soon as interacting individuals are related, there is scope for cooperation to evolve (see Hamilton, 1971; Boyd, 1982; Grafen, 1983; and other references in Wilson et al., 1992; Taylor, 1992a; and Taylor, 1992b, for exceptions). The idea was that positive relatedness means that cooperators are more likely to interact with cooperators than defectors are, which implies that, while cooperators pay the cost of cooperating, they will also be on the receiving end of cooperation more often. If they are indeed sufficiently much more often the recipients of cooperation than defectors are, and if the benefits are sufficiently large, then the cost of cooperation can be offset by the increase in benefits received.

What Wilson et al. (1992) and Taylor (1992a;b) discovered is that being around cooperators is not necessarily unambiguously good news. While being around more cooperators means receiving more cooperation – which is good – it can also mean being around individuals that cooperate more with one another, and therefore constitute more fierce competition – which is not good. For cases in which relatedness is caused by local dispersal, receiving more cooperation and facing more intense competition can go hand in hand, and therefore, if individuals have opportunities for cooperating that are as local as their competition is, no benefit is large enough to get costly cooperation to evolve. The main insight provided by Wilson et al. (1992) and Taylor (1992a;b) is therefore that it is not enough to be related; what is needed is a discrepancy between the relatednesses to the individuals with whom one has the opportunity to cooperate, and those with whom one has to compete. While the idea that positive relatedness alone would be enough for the evolution of cooperation was inspired by Hamilton's rule (Hamilton, 1964a;b), it was pointed out that, by defining the fitness effects of cooperation versus defection appropriately, the cancellation effect can actually be identified within the framework of Hamilton's rule; see Taylor (1992b); Grafen (2007); Allen et al. (2012), and Section 7 in van Veelen et al. (2017), all in settings where the game between individuals satisfies equal gains from switching.[1]

1.2 The cancellation effect at the group level

Group selection models aim at capturing the opposing effects of selection at the individual level, where defectors do better than cooperators within groups, and selection at the group level, where groups with more cooperators do better than groups with fewer cooperators (Sober and Wilson, 1998; Wilson and Wilson, 2007; Richerson et al., 2016). The existing models within the group selection literature share the property that competition between individuals happens within groups, and that competition between groups happens in a setting where all groups compete with all other groups equally intensely (Traulsen and Nowak, 2006; Simon, 2010; Simon et al., 2013; Luo, 2014; Luo and Mattingly, 2017; van Veelen et al., 2014). This last property is

[1] Ohtsuki (2012) analyzes a model that does not satisfy equal gains from switching. Other papers on the scale of cooperation versus the scale of competition are Queller (1992; 1994) and West et al. (2002), but they take a different approach. While Taylor (1992b); Grafen (2007); Allen et al. (2012) and van Veelen et al. (2017) include the cancellation effect by making sure to account for all fitness effects, and keeping the definition of relatedness the same, Queller (1992; 1994) and West et al. (2002) get Hamilton's rule to hold by changing the relatedness into *effective relatedness*. When we will do computations to include the cancellation effect at the group level, we will follow the first approach.

a useful simplification if the aim is to illustrate the possibility of a tug of war between the different levels of selection. It is, however, not particularly realistic. Groups themselves typically live in a structured population of groups, where they compete with their neighbouring groups more often than they do with groups that are farther apart. Local dispersal would then imply that groups with many cooperators are typically surrounded by groups that also contain many cooperators, compared to the groups that surround groups with many defectors, and therefore are also subject to more intense competition. This can significantly dampen the benefits of being a cooperative group, which in turn affects the balance between selection at the individual and at the group level.

In order to study the cancellation effect at the group level, and how it affects the balance between selection at different levels, we consider a stylized model, in which groups live on a cycle. We look at two replacement rules for groups. The first replacement rule is Birth-Death, where groups replace their direct neighbours. The second replacement rule is Shift, where a group at one position can reproduce, and a group anywhere else can die, and all groups in between the two just move over. With Birth-Death, groups compete with their direct neighbours, and the cancellation effect at the group level is the largest it can be. With Shift, there is no cancellation at the group level at all.

The rest of the Supporting Information is organized as follows. In Section 2, we describe the model, including both replacement rules. Analytical thresholds for the benefit-to-cost ratios in the limit of weak selection, both for Birth-Death and Shift, are derived in Section 3. These thresholds are expressed in terms of the parameters of the model and relatednesses. The relatednesses are endogenous themselves and also depend on the parameters of the model, so we compute these in Section 4. In Section 5, the benefit-to-cost ratios and the relatednesses are combined and it is shown that the critical benefit-to-cost ratio for the Birth-Death process is always higher than the critical benefit-to-cost ratio for the Shift process, making group selection models that assume away the cancellation effect more optimistic about the conditions for the evolution of cooperation. Section 6 describes the simulations. The simulation results, which are not in the limit of weak selection, are compared to the thresholds in the limit of weak selection in Section 7. In Section 8, we derive analytical results for some limits other than the limit of weak selection, and in Section 9 we discuss the empirical implications.

2 The model

2.1 Two update rules on the cycle

We consider a simple model, in which groups are situated on a cycle. Both the group size and the number of groups are fixed; at each point in time, there are m groups consisting of n individuals. Each individual can be either a cooperator (C) or a defector (D). In every time period, one of three types of events will happen: an individual can replace another individual within a group, a group can replace another group, or two individuals from two neighbouring groups can change places. These individual, group, and migration events happen with probabilities p, q and r, respectively, and, without loss of generality, we assume that $p + q + r = 1$.

In order to demonstrate the difference between global and local between-group competition, we compare two different replacement rules for group reproduction: Birth-Death (BD) and Shift. Competition between groups is local in BD, and global in Shift. Individual and migration events happen in the same way in both processes.

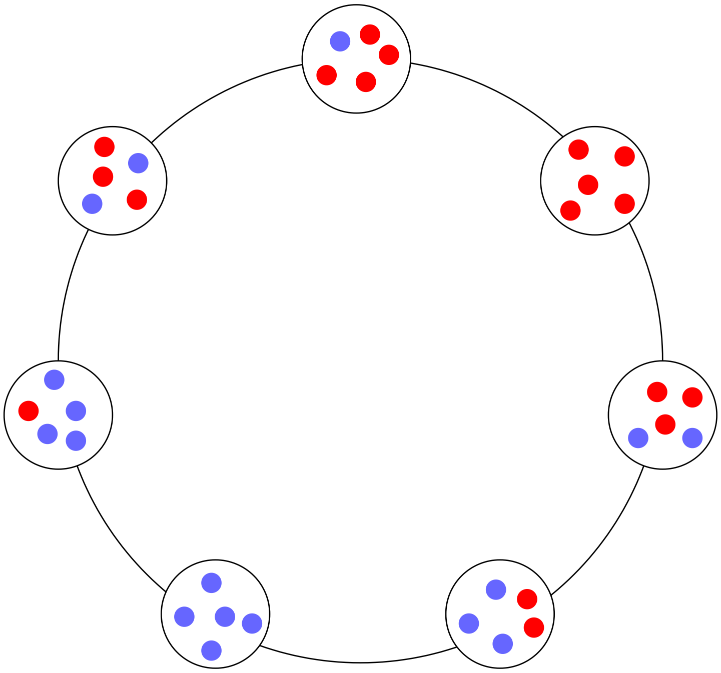

Figure 1: An example of a population state on a cycle with $m = 7$ groups of $n = 5$ individuals each. The blue dots indicate cooperators and the red dots indicate defectors. Each group has one right and one left neighbour.

2.1.1 Individual events

If an individual event occurs – which happens with probability p – then a random group is selected, and within that group, an individual is chosen to produce an identical offspring. All groups have equal probability of being chosen to host an individual level event. Within the group, defectors get an individual payoff of 1 and cooperators get an individual payoff of $1 - c$. The intensity of selection w is then used to transform these payoffs to values f_C and f_D:

$$f_C = 1 - w + w\,(1 - c) = 1 - wc \qquad \text{and} \qquad f_D = 1 - w + w\,(1) = 1$$

The probabilities with which individuals are chosen for reproduction within the group are proportional to these values.[2] The probability $p_C(k_i)$ that a cooperator is chosen, and the probability $p_D(k_i)$ that a defector is chosen, in a group with k_i cooperators, then become:

$$p_C(k_i) = \frac{k_i f_C}{k_i f_C + (n - k_i) f_D} = \frac{k_i(1 - wc)}{n - k_i wc}$$
$$p_D(k_i) = \frac{(n - k_i) f_D}{k_i f_C + (n - k_i) f_D} = \frac{n - k_i}{n - k_i wc} = 1 - p_C(k_i)$$

Whenever an individual reproduces, someone from the same group is chosen to die, where each individual, including the parent, but excluding the offspring, is chosen with probability $\frac{1}{n}$.

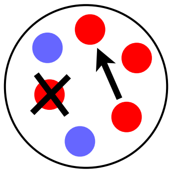

Figure 2: An example of the individual reproduction events. A defector is chosen for reproduction and produces an identical offspring. Another defector is chosen to die. In this case, the overall group composition has not changed.

If an individual event happens, the number of cooperators in that group can go up or down by one, or remain constant, depending on who reproduces and who dies within the group. Once a group reaches a state where all individuals are cooperators or all individuals are defectors, individual selection can not change the state of that group. Individual selection on its own, within a given group, therefore constitutes a Markov process with two absorbing states.

2.1.2 Group events

If a group event occurs – which happens with probability q – then one group is chosen to reproduce, and one group is chosen to die. Which group reproduces depends on the distribution of cooperators among groups. If the groups are numbered from 1 to m, then a population state is a vector k, in which $k_i \in \{0, 1, ..., n\}$ is the number of cooperators in group i, for $i = 1, ..., m$. Groups i and $i + 1$ are neighbouring groups, for $i = 1, ..., m - 1$, as well as groups 1 and m, which makes this a cycle. The group payoff of group i is $1 + \frac{k_i}{n} b$. The intensity of selection w is then used to transform these payoffs to values

$$g(k_i) = 1 - w + w\left(1 + \frac{k_i}{n} b\right) = 1 + w\frac{k_i}{n} b$$

[2] One could call these values "fitnesses", in line with Nowak (2006). We chose to not use that term here, and reserve the word fitness, and fitness effects, for (effects on) expected numbers of offspring.

Both in BD and in Shift, first a group is chosen for reproduction, where each group's probability of being chosen is proportional to their value $g(k_i)$ as given below.

$$q(k_i, K) = \frac{g(k_i)}{\sum_{j=1}^{m} g(k_j)} = \frac{1 + w\frac{k_i}{n}b}{n + w\frac{K}{n}b}$$

where $K = \sum_{j=1}^{m} k_j$ is the total number of cooperators in the population. If a group is chosen for reproduction, it produces an identical offspring group. Which group is being replaced depends on which replacement rule is used at the group level.

Birth-Death (BD)

The offspring group replaces its own parent group with probability $\frac{1}{m}$, and it replaces either the left or the right neighbour of the parent group, both with probability $\frac{m-1}{2m}$. If the offspring group replaces the parent group, the population state does not change. The possibility to replace the parent group is included in order to make the analytical comparison between the two processes more straightforward. This does not have any profound consequences; any process with that possibility is equivalent to a process without that possibility, and with a lower probability of having a group reproduction event at all.

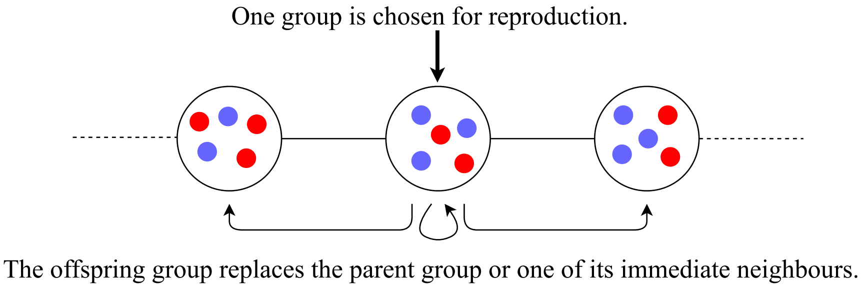

Figure 3: The BD process. One of the groups is chosen to reproduce, proportional to group values, and produces an identical offspring group. The offspring group replaces one of the neighbouring groups, or, with a small probability, it replaces the parent group itself.

Shift

Each group, including the parent group, but excluding the offspring group, is chosen to die with probability $\frac{1}{m}$. Hence, with Shift, every group is equally likely to die and competition between groups is therefore global, as it is in the standard group selection models with a well-mixed population of groups. Once a group dies, the offspring group is either placed to the right or to the left of the parent group, with equal probability, and every other group between the parent group and the dying group moves over one spot.

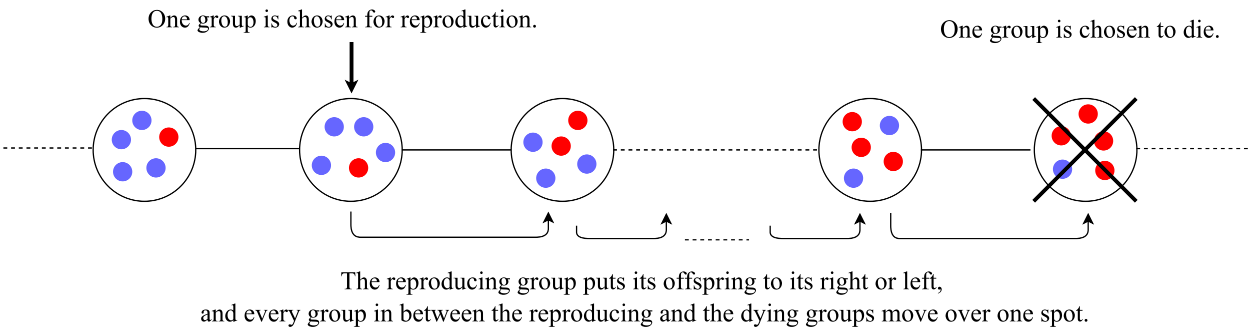

Figure 4: The Shift process. One of the groups is chosen to reproduce, proportional to group values, and produces an identical offspring group. The offspring replaces one of the groups chosen randomly from the whole pool of groups.

Group selection by itself also constitutes a Markov process with multiple absorbing states. Once all groups have the same fraction of cooperators, the population state can no longer change by group level events. Therefore, the states in which all groups have the same composition would be the absorbing states of a Markov process with group level events only.

2.1.3 Migration events

If a migration event occurs – which happens with probability r – then a random group is selected, and within that group, a random individual is chosen to migrate. All groups have equal probability of being chosen to host a migration event, and within the group, all individuals are equally likely to be chosen to become the migrant. Then a coin toss determines whether the individual moves to the group on the left or to the group on the right. Within the receiving group, a randomly chosen individual trades places with the first individual.

Migration makes a difference for which sets of states are absorbing. Without migration, the set that consists of all population states in which some groups consist of cooperators only, other groups consist of defectors only, but no group is mixed, is absorbing. Group events can still make a population transition from one population state within this set to another population state within this set, but no group or individual level event can make the population transition from a state within this set to one outside it. With migration, on the other hand, this is not an absorbing set of population states. When there is migration, the only sets of states that are absorbing are the set that only contains the state where everyone is a cooperator, and the set that only contains the state where everyone is a defector. Besides these two singleton sets, no sets of states are absorbing if we include migration.

This observation is important for understanding why, as we will see later, very low migration rates will reduce the gap between the critical b/c ratios for the two processes. Once absorbed within the set where all groups are homogeneous, the dynamics for the different replacement rules are only different in speed, while the differences in fixation probabilities disappear.

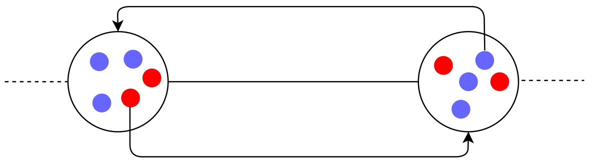

Figure 5: An example of a migration event, where a defector from the group on the left and a cooperator from the group on the right change places

2.2 Alternative modeling choices

There are places in the model where, with the literature on group selection in mind, one can easily think of other ways to determine who reproduces and who is replaced. The reason for the choices we made is typically that they allow us to derive relatively tractable analytical solutions, which help illustrate the cancellation effect. Alternative choices would produce the same gap between full cancellation at the group level (BD) and no cancellation at the group level (Shift), but some would come with (much) more complicated analytical solutions.

Luo (2014)

In Luo (2014) and van Veelen et al. (2014), the reproduction rate of an individual is an individual characteristic, where defectors have a higher individual reproduction rate than cooperators do. That implies that a group with more defectors is more likely to host an individual reproduction event than a group with fewer defectors. In our model, every group is equally likely to host an individual reproduction event. Another implication of the choices in Luo (2014) and van Veelen et al. (2014) is that there, the ratio of group level reproduction events to individual level reproduction events depends on how many cooperators there are in the population as a whole; with many cooperators, group level reproduction events happen more frequently, relative to individual level reproduction events, compared to a population with fewer cooperators. In our model, this ratio is $\frac{p}{q}$, which is constant.

The modeling choices in Luo (2014), and similar ones in Simon (2010) and Simon et al. (2013), produce a model that is in some respects more elegant than ours. When combined with a structured population of groups like the cycle, and with different replacement rules for group reproduction, the cancellation effect would be present and absent in their model in the same way as it is in our version. While our model may be a bit less elegant, it does allow for a more straightforward derivation of the formulas for the critical b/c ratios. (Just to be sure: groups in Luo (2014) and van Veelen et al. (2014) compete globally, making this otherwise equivalent to the Shift model without migration).

A final difference, not in the model itself, but in the approach to deriving analytical solutions, is that Simon (2010); Simon et al. (2013); Luo (2014), and van Veelen et al. (2014) all take limits of group size n and number of groups m going to infinity, while we derive analytical solutions in the limit of weak selection.

Traulsen & Nowak (2006)

In Traulsen and Nowak (2006), individuals within groups affect their own and each other's individual reproduction rates. Cooperators lower their own individual reproduction rate, and increase the individual

reproduction rates of their fellow group members. When a group is at maximimum capacity, there is a small probability that at an individual reproduction event, the offspring does not replace another group member, but makes the group split. Because individuals in all-cooperator groups reproduce more frequently than individuals in all-defector groups, all-cooperator groups also split more often, and therefore produce more offspring groups. This is a bit further removed from our model, but it would of course be possible to make versions of this model where groups are situated on a cycle, and, in case a group splits, it either replaces a neighbouring group (BD), or a randomly chosen group (Shift).

There are also differences in the methods used to derive analytical solutions. One reason not to use an extended version of their model is that their method for finding analytical solutions makes assumptions that make it hard to identify the cancellation effect at the group level by comparing Birth-Death and Shift. Traulsen and Nowak (2006) assume a separation of timescales, by considering the case where the probability that a group splits as a result of an individual reproduction event is vanishingly small. This implies that almost all of the time, groups will be at maximum capacity, and will consist of one type of individual only. The separation of timescales results in a nested Moran process, for which they compute fixation probabilities in the limit of weak selection. In Section 8.2 we take a similar approach by considering the limit of $p \to 1$, but without assuming selection to be weak. There, we find that the difference between Birth-Death and Shift disappears with the separation of timescales. The reason why it does is similar to the reason why it does without migration, when the dynamics also make groups be at within-group fixation almost all of the time. This is described in Section 7.

Traulsen and Nowak (2006) also have a version with (global) migration. In the limit they consider, migration events happen with a frequency that is of the same order of magnitude as splitting events. This leaves the separation of timescales intact, which implies that groups will still either be all-cooperator or all-defector groups most of the time. For identifying a difference between Birth-Death and Shift, we would need migration to occur sufficiently frequently to keep at least some groups away from within group fixation. Although their model does not allow for a straightforward extension to a structured population of groups, in which we can easily identify the cancellation effect at the group level, we do find a few consistent patterns. The number of groups m, group size n, and migration rate r all affect the critical b/c ratio in our model in ways that are similar to how they affect that ratio in their model.

Public goods game

Although the game in our model does have the key properties of a public goods game, where paying individual costs come with collective benefits, one can also define payoffs so that the game looks like a public goods game already at the individual level. Such a version of the model would leave all model assumptions unchanged, except for the individual and group payoff functions. The values for a cooperator and a defector in group i, in which there are k_i cooperators, then become:

$$f_C^{PGG}(k_i) = 1 - w + w\left(1 + \frac{k_i}{n}b - c\right) = 1 + w\left(\frac{k_i}{n}b - c\right)$$

and

$$f_D^{PGG}(k_i) = 1 - w + w\left(1 + \frac{k_i}{n}b\right) = 1 + w\left(\frac{k_i}{n}b\right)$$

where w is the intensity of selection. The value for the group is defined as:

$$g(k_i)^{PGG} = 1 - w + w\left(1 + \frac{k_i}{n}(b-c)\right) = 1 + w\left(\frac{k_i}{n}(b-c)\right)$$

where w is the intensity of selection. We assume that $b > c$.

In this formulation, the payoffs seem to make this a public goods game, already at the individual level, as all individuals get a higher payoff when all individuals play C (when they all get $b - c$), compared to what all individuals get when all play D (when they all get 0). In our setting, this is however not really an improvement for all at the individual level. The probability of a group being chosen to host an individual event is $\frac{1}{m}$, and this probability is therefore independent of the population state. When all individuals have the same payoff, whether it is all large or all small, their probability of reproducing, conditional on their group being chosen to host the individual reproduction event, is $\frac{1}{n}$. These payoffs therefore only seemingly introduce the public goods nature at the individual level. In alternative settings, where groups with many cooperators also host more individual reproduction events than groups with many defectors, the reproduction rates of everyone will be higher if everyone is a cooperator rather than everyone being a defector, but now this is compensated by an also elevated death rate, again neutralizing all "gains from cooperation" at the individual reproduction level. The only difference between the formulations is how the change in individual reproduction rate depends on the total number of cooperators in the group. In the formulation we chose, being a cooperator and not a defector decreases ones individual reproduction rate by $\frac{1}{n-wk_ic} - \frac{1-wc}{n-w(k_i+1)c}$, if k_i is the number of cooperators among the other members of the group. In this "PGG" formulation, that difference is $\frac{1+w\frac{k_i}{n}b}{n+wk_i(b-c)} - \frac{1+w\left(\frac{k_i+1}{n}b-c\right)}{n+w(k_i+1)(b-c)}$. In other words, in our formulation, the reduction in individual reproduction rate gets a bit larger when more others cooperate; while in this alternative PGG formulation, the reduction in individual reproduction rate gets a bit smaller.

This alternative formulation does furthermore link individual and group values, by making the latter the average of the former. The gains in group reproduction rate relate in a more straightforward way; the $b - c$ in the PGG version replaces the b in our version.

Given that the effective differences between these two versions of the model are only minor details, it is not surprising that simulations also give very similar results. For obtaining analytic results, however, our version is easier to work with.

3 Analytical results in the limit of weak selection

We would like to derive critical b/c ratios, above which cooperation is selected for, in the limit of weak selection. In order to do that, we will go over the effects of being a cooperator instead of a defector on reproduction rates and death rates. The b and the c are just model parameters, so they are not the fitness benefits and fitness costs of cooperation. The effects that we compute below do amount to those fitness benefits and costs. Because these effects satisfy equal gains from switching locally (they are additive in the limit of weak selection), we can follow an inclusive fitness approach, where these effects are weighted with the relatednesses to the individual that the effects are on, in order to determine the direction of selection (van Veelen et al., 2017; van Veelen, 2018). We will use this approach to derive critical b/c ratios, which are therefore formulated in terms of model parameters. In the following subsections, we consider a case where one individual switches from being a defector to being a cooperator, and we calculate the effects of this change by the focal individual on every individual in the population, weighted by the corresponding relatednesses.

Since the probabilities concerning reproduction events, both at the individual level and at the group level, are the same in BD and in Shift, the effects of being a cooperator instead of a defector on reproduction rates will be the same for both. Moreover, in both processes, the individual death rate is the same. The difference between the two processes, therefore, will only start showing up when we compute the effects on group death rates.

3.1 Changes in reproduction rates

3.1.1 Changes in individual reproduction rates

Changes in my reproduction rate

If I am a C instead of a D, with i other C players in my group, and my group is chosen for an individual update, I change my probability of being chosen for reproduction from $\frac{1}{n-iwc}$ to $\frac{1-wc}{n-(i+1)wc}$, which is a difference of $\frac{1-wc}{n-(i+1)wc} - \frac{1}{n-iwc}$. If we take the derivative with respect to w for both terms, we get:

$$\frac{d\left(\frac{1-wc}{n-(i+1)wc}\right)}{dw} = -\frac{n-(i+1)}{(n-(i+1)wc)^2} \cdot c \qquad \text{and} \qquad \frac{d\left(\frac{1}{n-iwc}\right)}{dw} = \frac{i}{(n-iwc)^2} \cdot c$$

Evaluated at $w = 0$, we get:

$$\left.\frac{d\left(\frac{1-wc}{n-(i+1)wc}\right)}{dw}\right|_{w=0} = -\frac{n-(i+1)}{n^2} \cdot c \qquad \text{and} \qquad \left.\frac{d\left(\frac{1}{n-iwc}\right)}{dw}\right|_{w=0} = \frac{i}{n^2} \cdot c$$

which means that the change in probability of being chosen for reproduction, for w close to 0 (weak selection), can be approximated by

$$\frac{1-wc}{n-(i+1)wc}-\frac{1}{n-iwc}\approx -\frac{n-1}{n^2}c\cdot w$$

Changes in the reproduction rate of a C in my group

If I am a C instead of a D, with i other C players in my group, and my group is chosen for an individual update, I change other C's individual probability of being chosen for reproduction from $\frac{1-wc}{n-iwc}$ to $\frac{1-wc}{n-(i+1)wc}$, which is a difference of $\frac{1-wc}{n-(i+1)wc}-\frac{1-wc}{n-iwc}$. If we take the derivative with respect to w, we get:

$$\frac{d\left(\frac{1-wc}{n-(i+1)wc}\right)}{dw}=-\frac{n-(i+1)}{(n-(i+1)wc)^2}\cdot c \qquad \text{and} \qquad \frac{d\left(\frac{1-wc}{n-iwc}\right)}{dw}=-\frac{n-i}{(n-iwc)^2}\cdot c$$

Evaluated at $w=0$, we get:

$$\left.\frac{d\left(\frac{1-wc}{n-(i+1)wc}\right)}{dw}\right|_{w=0}=-\frac{n-(i+1)}{n^2}\cdot c \qquad \text{and} \qquad \left.\frac{d\left(\frac{1-wc}{n-iwc}\right)}{dw}\right|_{w=0}=-\frac{n-i}{n^2}\cdot c$$

which means that the change in their probability of being chosen for reproduction, for w close to 0 (weak selection), can be approximated by

$$\frac{1-wc}{n-(i+1)wc}-\frac{1-wc}{n-iwc}\approx \frac{1}{n^2}c\cdot w$$

Changes in the reproduction rate of a D in my group

If I am a C instead of a D, with i other C players in my group, and my group is chosen for an individual update, I change other D's individual chance of being chosen for reproduction from $\frac{1}{n-iwc}$ to $\frac{1}{n-(i+1)wc}$, which is a difference of $\frac{1}{n-(i+1)wc}-\frac{1}{n-iwc}$. If we take the derivative with respect to w, we get:

$$\frac{d\left(\frac{1}{n-(i+1)wc}\right)}{dw}=\frac{i+1}{(n-(i+1)wc)^2}\cdot c \qquad \text{and} \qquad \frac{d\left(\frac{1}{n-iwc}\right)}{dw}=\frac{i}{(n-iwc)^2}\cdot c$$

Evaluated at $w=0$, we get:

$$\left.\frac{d\left(\frac{1}{n-(i+1)wc}\right)}{dw}\right|_{w=0}=\frac{i+1}{n^2}\cdot c \qquad \text{and} \qquad \left.\frac{d\left(\frac{1}{n-iwc}\right)}{dw}\right|_{w=0}=\frac{i}{n^2}\cdot c$$

which means that the change in their probability of being chosen for reproduction, for w close to 0 (weak selection), can be approximated by

$$\frac{1}{n-(i+1)wc} - \frac{1}{n-iwc} \approx \frac{1}{n^2}c \cdot w$$

The effects on individual reproduction rates should be multiplied by $p\frac{1}{m}$ in order to account for the probability with which an individual event happens, and that my group is chosen to host it. The changes in individual reproduction rates within the group add up to 0, as they should with a fixed group size. There are no effects on individual reproduction rates in other groups.

3.1.2 Changes in group reproduction rates

Changes in my group's reproduction rate

If I am a C instead of a D, with i other C players in my group, $i = 0, 1, ..., n-1$, and j other C's in the population as a whole, $j = i, i+1, ..., i+n(m-1)$, then if a group level event happens, I change my group's probability of being chosen for reproduction from $\frac{1+w\frac{i}{n}b}{m+w\frac{j}{n}b}$ to $\frac{1+w\frac{i+1}{n}b}{m+w\frac{j+1}{n}b}$, which is a difference of $\frac{1+w\frac{i+1}{n}b}{m+w\frac{j+1}{n}b} - \frac{1+w\frac{i}{n}b}{m+w\frac{j}{n}b}$. If we take the derivative of both terms with respect to w, we get:

$$\frac{d\left(\frac{1+w\frac{i+1}{n}b}{m+w\frac{j+1}{n}b}\right)}{dw} = \frac{\frac{m(i+1)-(j+1)}{n}}{\left(m+w\frac{j+1}{n}b\right)^2} \cdot b \qquad \text{and} \qquad \frac{d\left(\frac{1+w\frac{i}{n}b}{m+w\frac{j}{n}b}\right)}{dw} = \frac{\frac{mi-j}{n}}{\left(m+w\frac{j}{n}b\right)^2} \cdot b$$

Evaluated at $w = 0$, we get:

$$\left.\frac{d\left(\frac{1+w\frac{i+1}{n}b}{m+w\frac{j+1}{n}b}\right)}{dw}\right|_{w=0} = \frac{m(i+1)-(j+1)}{nm^2} \cdot b \qquad \text{and} \qquad \left.\frac{d\left(\frac{1+w\frac{i}{n}b}{m+w\frac{j}{n}b}\right)}{dw}\right|_{w=0} = \frac{mi-j}{nm^2} \cdot b$$

which means that the change in my group's probability of being chosen for reproduction, for w close to 0 (weak selection), can be approximated by

$$\frac{1+w\frac{i+1}{n}b}{m+w\frac{j+1}{n}b} - \frac{1+w\frac{i}{n}b}{m+w\frac{j}{n}b} \approx \frac{m-1}{nm^2}b \cdot w$$

Changes in other groups' reproduction rates

If I am a C instead of a D, with i other C players in my group, $i = 0, 1, ..., n-1$, and j other C's in the population as a whole, $j = i, i+1, ..., i+n(m-1)$, then if a group level event happens, I change the probability of being chosen for reproduction of a random other group with k cooperators from $\frac{1+w\frac{k}{n}b}{m+w\frac{j}{n}b}$ to $\frac{1+w\frac{k}{n}b}{m+w\frac{j+1}{n}b}$, which is a difference of $\frac{1+w\frac{k}{n}b}{m+w\frac{j+1}{n}b} - \frac{1+w\frac{k}{n}b}{m+w\frac{j}{n}b}$. If we take the derivative with respect to w, we get:

$$\frac{d\left(\frac{1+w\frac{k}{n}b}{m+w\frac{j+1}{n}b}\right)}{dw}=\frac{\frac{mk-(j+1)}{n}}{\left(m+w\frac{j+1}{n}b\right)^2}\cdot b \qquad \text{and} \qquad \frac{d\left(\frac{1+w\frac{k}{n}b}{m+w\frac{j}{n}b}\right)}{dw}=\frac{\frac{mk-j}{n}}{\left(m+w\frac{j}{n}b\right)^2}\cdot b$$

Evaluated at $w = 0$, we get:

$$\left.\frac{d\left(\frac{1+w\frac{k}{n}b}{m+w\frac{j+1}{n}b}\right)}{dw}\right|_{w=0}=\frac{mk-(j+1)}{nm^2}\cdot b \qquad \text{and} \qquad \left.\frac{d\left(\frac{1+w\frac{k}{n}b}{m+w\frac{j}{n}b}\right)}{dw}\right|_{w=0}=\frac{mk-j}{nm^2}\cdot b$$

which means that the change in that other group's probability of being chosen for reproduction, for w close to 0 (weak selection), can be approximated by

$$\frac{1+w\frac{k}{n}b}{m+w\frac{j+1}{n}b}-\frac{1+w\frac{k}{n}b}{m+w\frac{j}{n}b}\approx-\frac{1}{nm^2}b\cdot w$$

The effects on group reproduction rates should be multiplied by q in order to account for the probability with which a group event happens. The changes in group reproduction rates add up to 0, as they should with a fixed number of groups.

3.1.3 Overall effect through changes in reproduction rates

The combined effects, close to neutrality, all weighted with the corresponding relatednesses, are:

$$\frac{p}{m}\left(-\frac{n-1}{n^2}+(n-1)r_s\frac{1}{n^2}\right)c\cdot w+q\left(\frac{m-1}{nm^2}+(n-1)r_s\frac{m-1}{nm^2}-n(m-1)r_o\frac{1}{nm^2}\right)b\cdot w$$

where r_s is the relatedness between an individual and a randomly chosen other individual from the same group, and r_o is the relatedness between an individual and a randomly chosen individual from a randomly chosen other group.

In Section 4, we derive identities concerning different relatednesses. We can use one of those to rewrite the combined effects. Equation (39) states that $r_o=-\frac{1}{n(m-1)}-\frac{n-1}{n(m-1)}r_s$, and using that, we can see rewrite the combined effects as

$$-\frac{p}{m}\frac{n-1}{n^2}(1-r_s)c\cdot w+q\frac{1}{nm}\left(1+(n-1)r_s\right)b\cdot w$$

Equation (8) moreover states that $r_0=\frac{1}{n}+\frac{n-1}{n}r_s$, and therefore that $\frac{n-1}{n}(1-r_s)=1-r_0$ and $1+(n-1)q_s=nq_0$, where r_0 is the relatedness between an individual and a randomly chosen individual from the same group – which is labeled group 0 – including the individual itself. The only difference with r_s is that there the

individual itself is excluded, hence the simple relation between r_0 and r_s. We can use this to rewrite the combined effects as

$$-\frac{p}{m}\frac{1}{n}(1-r_0)c \cdot w + q\frac{1}{nm}(nr_0)\, b \cdot w$$

3.2 Changes in death rates

3.2.1 Changes in individual death rates

Individual death rates do not change when a player switches from being D to C.

3.2.2 Changes in group death rates

Changes in group death rates differ between the two update processes at the group level.

Birth-Death

Changes in my group's death rate

If I am a C instead of a D, with i other C players in my group, $i = 0, 1, ..., n-1$, and j other C's in the population as a whole, $j = i, i+1, ..., i+n(m-1)$, then, if a group level event happens, we have seen that I change the reproduction rate of my own group by approximately $\frac{m-1}{nm^2} b \cdot w$. Since with probability $\frac{1}{m}$ the offspring group replaces the parent group, that comes with an increase in death rate equal to

$$\frac{1}{m}\frac{m-1}{nm^2} b \cdot w$$

We have also seen that I change the reproduction rate of all other groups by approximately $-\frac{1}{nm^2} b \cdot w$, including my two neighbouring groups. If one of those is chosen for reproduction, it replaces my group with probability $\frac{m-1}{2m}$, reducing my death rate by

$$2 \cdot \frac{1}{2}\frac{m-1}{m}\frac{1}{nm^2} b \cdot w$$

These two cancel out exactly, so the overall effect on the death rate of my group is 0, as my neighbours are now less likely to be chosen for reproduction and replace my group, but my group is more likely to reproduce and replace itself.

Changes in the death rate of the two neighbouring groups

Following a similar argument, we find that the probability that one of my next-door neighbour groups is replaced changes by:

$$\frac{1}{2}\frac{m-1}{m}\frac{m-1}{nm^2} b \cdot w - \frac{1}{2}\frac{m-1}{m}\frac{1}{nm^2} b \cdot w - \frac{1}{m}\frac{1}{nm^2} b \cdot w$$

The first term is the product of the probability that, if my group is chosen for reproduction, it replaces a given neighbour, and the increase in my groups probability to reproduce. The second term is the product of the probability of the neighbouring group of the neighbouring group to replace the neighbouring group, if chosen for reproduction, and the decrease in their probability of being chosen for reproduction. The third term is the product of the probability of the neighbouring group to replace itself, if chosen for reproduction, and the decrease in their probability of being chosen for reproduction. This sum can be rewritten as:

$$\left(\frac{1}{2}(m-1)(m-2)-1\right)\frac{1}{nm^3}b\cdot w=\frac{1}{2}(m-3)\frac{1}{nm^2}b\cdot w$$

This effect is the same for both right and left next-door neighbours.

Changes in the death rates of other groups

The probability of being replaced changes by:

$$2\cdot-\frac{1}{2}\frac{m-1}{m}\frac{1}{nm^2}b\cdot w-\frac{1}{m}\frac{1}{nm^2}b\cdot w=-\frac{1}{nm^2}b\cdot w$$

for any of the $m-3$ other groups. The first term on the left hand side is twice the product of the probability that a given neighbouring group replaces a given group, times the change in reproduction probability of those neighbouring groups. The second term is the product of the probability a group replaces its parent group, when chosen for reproduction, and the change in reproduction probability of such a group.

The effects on group reproduction rates should be multiplied by q in order to account for the probability with which a group event happens.

Shift

Changes in group death rates

For the Shift process, group death rates do not change when a player switches from being D to C.

3.2.3 Overall effect through changes in death rates

Birth-Death

The overall effect through death rates again combines the effects on individual death rates (which are zero) and the effects on group death rates affecting self, group members, and members of other groups, each weighted with the corresponding relatedness measure:

$$\frac{p}{m}\cdot 0 + q\left(0-(n-1)r_s\cdot 0 + nr_1\frac{1}{2}\frac{m-3}{nm^2}b\cdot w - n\left(\sum_{i=2}^{m-2} r_i\right)\frac{1}{nm^2}b\cdot w + nr_{m-1}\frac{1}{2}\frac{m-3}{nm^2}b\cdot w\right)$$
$$= q\left(nr_1\frac{1}{2}\frac{m-3}{nm^2} + nr_1\frac{1}{nm^2} - n\left(\sum_{i=1}^{m-1} r_i\right)\frac{1}{nm^2} + nr_{m-1}\frac{1}{nm^2} + nr_{m-1}\frac{1}{2}\frac{m-3}{nm^2}\right)b\cdot w$$
$$= q\left(nr_1\frac{m-3}{nm^2} + 2nr_1\frac{1}{nm^2} - n\left(\sum_{i=1}^{m-1} r_i\right)\frac{1}{nm^2}\right)b\cdot w$$
$$= q\left(nr_1\frac{m-1}{nm^2} + (1+(n-1)r_s)\frac{1}{nm^2}\right)b\cdot w$$
$$= q\frac{1}{nm^2}\left(n(m-1)r_1 + 1 + (n-1)r_s\right)b\cdot w$$
$$= q\frac{1}{nm^2}\left(n(m-1)r_1 + nr_0\right)b\cdot w$$

where we used the identity $r_1 = r_{m-1}$, and Equations (37), and (8) from Section 4.

Shift

For Shift, the overall effect through changes in death rates is zero, since the effects on death rates of both individuals and groups are zero.

3.3 Overall effect of switching from D to C

The overall effect of a player switching from playing D to C would be the effect on reproduction rates minus the effect on death rates.

3.3.1 Birth-Death

The overall effect for the BD process is

$$-p\frac{1}{mn}(1-r_0)c\cdot w + q\frac{m}{nm^2}\left(nr_0\right)b\cdot w - q\frac{1}{nm^2}\left(n(m-1)r_1 + nr_0\right)b\cdot w$$
$$= -p\frac{1}{mn}(1-r_0)c\cdot w + q\frac{1}{nm^2}((m-1)(nr_0) - (m-1)nr_1)b\cdot w$$
$$= -p\frac{1}{mn}(1-r_0)c\cdot w + q\frac{m-1}{nm^2}(nr_0 - nr_1)b\cdot w$$

This effect is positive if

$$-p\frac{1}{m}(1-r_0)\frac{1}{n}c + q\frac{m-1}{m}(nr_0 - nr_1)\frac{1}{nm}b > 0$$

This gathers the effects as summarized in Fig. 6. The probability that an individual event happens is p. The probability that if it does, it happens in the group of the focal individual is $\frac{1}{m}$. If we write the effect on the individual reproduction rate of the focal individual as $-\frac{1}{n}c + \frac{1}{n^2}c$, then we can also see the individual

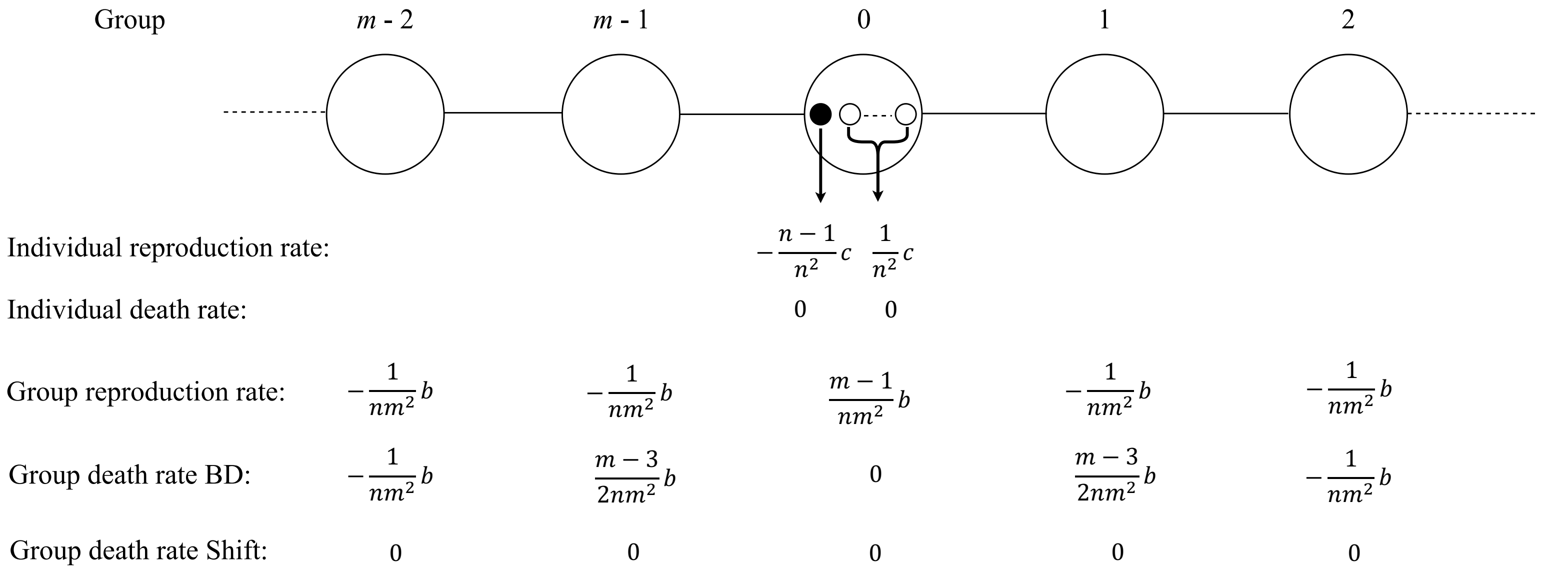

Figure 6: An overview of all the fitness effects in the limit of weak selection, conditional on an individual event happening in the group of the focal individual, or a group event happening, respectively. The black dot represents the focal individual.

effects as a combination of a reduction in individual reproduction rate of the focal individual by $\frac{1}{n}c$, and an increase in individual reproduction rate of $\frac{1}{n^2}c$ for everyone in the group, including the focal individual. With n individuals per group, the latter is equivalent to an effect of $\frac{1}{n}c$ on a randomly chosen individual from the same group, including the focal individual. This randomly chosen individual is related r_0 to the focal individual.

The probability that a group event happens is q. For all groups other than the focal group and its direct neighbours, the group reproduction rate and the group death rate go down by the same amount. The difference between the change in group reproduction rate and the change in the group death rate is $\frac{m-1}{nm^2}b$ for the focal group, and $-\frac{1}{2}\frac{m-1}{nm^2}b$ for both neighbouring groups, which adds up to $-\frac{m-1}{nm^2}b$. In the group of the focal individual, there are n individuals, who on average are related r_0 to the focal individual, and also in the neighbouring group there are n individuals, and those are related r_1 to the focal individual.

One way to rewrite this inequality would be

$$-p\frac{1}{m}\left(1-r_0\right)\frac{1}{n}c+qn\left(r_0-\left(\frac{1}{m}r_0+\frac{m-1}{m}r_1\right)\right)\frac{1}{nm}b>0 \tag{1}$$

The term $qnr_0\frac{1}{nm}b$ now reflects the effects through changes in group reproduction rates, while the term $-qn\left(\frac{1}{m}r_0+\frac{m-1}{m}r_1\right)\frac{1}{nm}b$ reflects the effects through changes in group death rates. This matches the group replacement rule for Birth-Death, where a reproducing group replaces itself with probability $\frac{1}{m}$, and one of its neighbouring groups with probability $\frac{m-1}{m}$.

Another way to rewrite this condition would be

$$q\frac{m-1}{m}n(r_0-r_1)b > p(1-r_0)c$$

which implies that, in the BD process, cooperation is selected for if

$$\frac{b}{c} > \frac{p}{q}\frac{m}{m-1}\frac{1-r_0}{n(r_0-r_1)} \tag{2}$$

3.3.2 Shift

Since the effect on death rates is zero, the overall effect would be equal to the effect on reproduction rates:

$$-p\frac{1}{mn}(1-r_0)c\cdot w + q\frac{1}{nm}\left(nr_0\right)b\cdot w$$

This effect is positive if

$$-p\frac{1}{m}(1-r_0)\frac{1}{n}c + qnr_0\frac{1}{nm}b > 0 \tag{3}$$

This gathers the effects for Shift, which are also summarized in Fig. 6. The effects on individual reproduction rates are the same as for Birth-Death. On the group level, there are now only changes in reproduction rates and no changes in death rates. The changes in group reproduction rates can be seen as a combination of an increase in group reproduction rate of the group of the focal individual by $\frac{1}{nm}c$, and a decrease in group reproduction rate of $\frac{1}{nm^2}c$ for every group, including the group of the focal individual. The latter is equivalent to an effect of $\frac{1}{nm}c$ on a randomly chosen group, including the group of the focal individual. A randomly chosen individual from the same group, including the focal individual itself, is related r_0 to the focal individual, and a randomly chosen individual from a randomly chosen group, including the group of he focal individual, is related $\sum_{i=0}^{m-1}\frac{1}{n}r_i = 0$ to the focal individual.

If we simplify this condition, we get

$$qnr_0b > p(1-r_0)c$$

which implies that, in the Shift process, cooperation is selected for if

$$\frac{b}{c} > \frac{p}{q}\frac{1-r_0}{nr_0} \tag{4}$$

3.4 Comparing thresholds

There are two differences between these two thresholds. To pinpoint the first, we can write Condition (2), which gives the threshold for Birth-Death, as

$$\frac{b}{c} > \frac{p}{q} \frac{1 - r_0}{n \left(r_0 - \left(\frac{1}{m} r_0 + \frac{m-1}{m} r_1 \right) \right)}$$

This condition has has a $-\left(\frac{1}{m}r_0 + \frac{m-1}{m}r_1\right)$ term that is absent in Condition (4). This term reflects the cancellation effect, and it pushes the threshold up. The second difference is that r_0 will not the same across the two processes, even if everything else (that is: p, q, r, n and m) is equal. In Section 4, we calculate how r_s, and thereby also r_0, depends on those five parameters for both processes, and it turns out that r_0 is higher for BD than for Shift. Therefore, if it was not for the first difference, the critical b/c ratio would actually be *lower* for BD than for Shift.

3.5 Hamilton's rule

We derived these thresholds using inclusive fitness. Therefore, it is worth pointing out that the b and the c in those thresholds are model parameters, and that they are *not* the fitness benefits and fitness costs of cooperation. It would therefore not be correct to read these formulas themselves as versions of Hamilton's rule, where the b and the c would represent the fitness benefits and costs, and the right hand side would replace $\frac{1}{r}$ (Ohtsuki et al., 2006; Nowak et al., 2010). These parameters do however determine the size of the fitness effects, as we have seen in the derivations that preceded Conditions (2) and (4).

3.6 More general version and interpretation

The processes we consider are Birth-Death, with completely local between-group competition, and Shift, with completely global between-group competition. One can however also consider a more general class of processes that are the same as Birth-Death and Shift with respect to their individual reproduction, but that vary in how local between-group competition is. To keep them comparable, we can assume for all processes that if a group is chosen to reproduce, then the parent group itself is chosen to die with probability $\phi_0 = \frac{1}{m}$. The remainder of the probabilities can be chosen freely, and are given by $\phi_i, i = 1, ..., m-1$, where we do assume symmetry ($\phi_j = \phi_{m-j}$) and, since they are probabilities, $\sum_{i=0}^{m-1} \phi_i = 1$. Groups between the reproducing group and the dying group then move over in the same way as they do in Shift.

3.6.1 Changes in group death rates

For such a process, we can write the effect through the change in death rate of group i as a sum of the change in its death rate as a result of the focal group reproducing, and the changes as a result of all other groups reproducing:

$$\frac{m-1}{nm^2}b\cdot\phi_i\cdot w+\sum_{j=1}^{m-1}-\frac{1}{nm^2}b\cdot\phi_{|i-j|}\cdot w$$

This can be rewritten as

$$\begin{aligned}&\left(\frac{1}{nm}-\frac{1}{nm^2}\right)b\cdot\phi_i\cdot w+\sum_{j=1}^{m-1}-\frac{1}{nm^2}b\cdot\phi_{|i-j|}\cdot w\\ &=\frac{1}{nm}b\cdot\phi_i\cdot w+\sum_{j=0}^{m-1}-\frac{1}{nm^2}b\cdot\phi_{|i-j|}\cdot w\\ &=\frac{1}{nm}b\cdot\phi_i\cdot w-\frac{1}{nm^2}b\cdot w\end{aligned}$$

Here, we use $\sum_{j=0}^{m-1}\phi_{|i-j|}=\sum_{j=0}^{m-1}\phi_j=1$.

3.6.2 Overall effect of switching from D to C

The overall effect then becomes

$$\begin{aligned}&-p\frac{1}{mn}(1-r_0)c\cdot w+q\frac{m}{nm^2}\left(nr_0\right)b\cdot w-q\cdot n\sum_{i=0}^{m-1}\left(\frac{1}{nm}b\cdot\phi_i\cdot w-\frac{1}{nm^2}b\cdot w\right)r_i\\ &=-p\frac{1}{mn}(1-r_0)c\cdot w+q\frac{1}{nm}\left(nr_0-n\sum_{i=0}^{m-1}\phi_i r_i\right)b\cdot w\end{aligned}$$

Here we use $\sum_{i=0}^{m-1}r_i=0$. This is positive if

$$-p\frac{1}{m}(1-r_0)\frac{1}{n}c+q\left(nr_0-n\sum_{i=0}^{m-1}\phi_i r_i\right)\frac{1}{nm}b>0 \tag{5}$$

Birth-Death is a special case of this larger collection with $\phi_0=\frac{1}{m}$, $\phi_1=\phi_{m-1}=\frac{m-1}{2m}$ and $\phi_i=0$, for $i=2,...,m-2$. To get Condition (1) for Birth-Death, we use $r_1=r_{m-1}$. Shift is a special case with $\phi_i=\frac{1}{m}$ for all i, and to get Condition (3), we use $\sum_{i=0}^{m-1}r_i=0$.

In the descriptions of how Conditions (1) and (3) summarize the fitness effects, we have seen that the first term in both of them, which is also the first term here, summarizes the effects of changes in individual reproduction rates. We have also seen how the first half of the second term, $qnr_0\frac{1}{nm}b$, summarizes the effects of changes in group reproduction rates. The second half of the second term, $-qn\sum_{i=0}^{m-1}\phi_i r_i\frac{1}{nm}b$, finally, summarizes the effect of changes in the group death rates. This term therefore captures the cancellation effect at the group level for these models.

We can also write the condition as

$$\frac{b}{c} > \frac{p}{q} \frac{1 - r_0}{n\left(r_0 - \sum_{i=0}^{m-1} \phi_i r_i\right)} \tag{6}$$

It should be noted though that the relatednesses are endogenous; they also depend on the process. Since we focus on Birth-Death and Shift, we only compute relatednesses for those.

The whole of Section 3.6 follows a very helpful suggestion by one of the reviewers. Also the use of Equation (8) earlier on, and the focus on and interpretation of Conditions (1) and (3) follow suggestions of this reviewer.

4 Relatedness

In this section, we will calculate relatednesses r_i between individuals that are i groups apart, both for Birth-Death and for Shift, in the limit of weak selection, using identical-by-descent (IBD) probabilities. Two individuals are considered IBD if they descend from a common ancestor, and no mutations have occurred along their lineage. We will first derive the relatedness measures by assuming a mutation probability u per individual reproduction (and, at a group reproduction event, all individuals in the group reproduce with the same mutation probability), and then take the limit of $u \downarrow 0$ to find the no-mutation limit of relatedness measures. To do so, we will first derive the recurrence relations for IBD probabilities by assuming a stationary distribution $\{q_i\}$ and then derive the no-mutation limit for relatedness using the following identity (Malécot, 1948; Rousset, 2004; Taylor et al., 2007a;b; Grafen, 2007; Durrett, 2008)

$$r_i = \frac{q_i - \bar{q}}{1 - \bar{q}} \tag{7}$$

where q_i denotes the stationary IBD probability for two individuals whose groups are i steps apart and $\bar{q}$ denotes the average IBD probability of a focal individual to all the individuals in the population, including self.

Two observations will be useful in our later derivations. The first is that, by symmetry, $q_i = q_{m-i}$ for $0 \leq i \leq m$. The second is that, for $i = 0$, we can relate q_0 – the IBD probability for two members from the same group, drawn with replacement – and q_s – the same probability without replacement – in a straightforward way:

$$q_0 = \frac{1 + (n-1)q_s}{n}. \tag{8}$$

since the individual's relatedness to herself is 1.

4.1 Birth-Death

In the BD process, an individual can be replaced if:

- An individual event happens, the group the individual is in is chosen to host it, and within the group, she is chosen to be replaced.
- A group event happens, one of the neighbouring groups of the group the individual is in is chosen to reproduce, and that group replaces her group.
- A group event happens, the group that the individual belongs to is chosen, and replaces itself.
- A migration event occurs, the individual's group is chosen to be one of the groups in which the migration takes place, and the individual is chosen to be swapped.

Combining these events, we can derive the recurrence relations for the stationary IBD probabilities.

4.1.1 $i = 1$

For $i = 1$, we have the following recurrence relation:

$$q_1 = \underbrace{p\frac{2}{m}\frac{1}{n}(1-u)q_1}_{\substack{\text{replaced in an}\\ \text{individual event}}} + \underbrace{q\left(\frac{1}{m}\frac{m-1}{m}(1-u)(q_0+q_2) + \frac{2}{m^2}(1-u)q_1\right)}_{\text{replaced in a group event}} + \underbrace{r\frac{1}{m}\frac{1}{n}\left(2q_2 + \frac{2(n-1)}{n}q_s + \frac{1}{n}q_1\right)}_{\text{replaced in a migration event}}$$

$$+ \left(\underbrace{p\left(\frac{m-2}{m} + \frac{2}{m}\frac{n-1}{n}\right)}_{\substack{\text{no change through}\\ \text{individual events}}} + \underbrace{q\left(\frac{m-4}{m} + \frac{m+1}{m^2} + \frac{m-1}{m^2}\right)}_{\text{no change through group events}} + \underbrace{r\left(\frac{m-3}{m} + \frac{2}{m}\frac{n-1}{n} + \frac{1}{m}\frac{(n-1)^2}{n^2}\right)}_{\text{no change through migration events}}\right) q_1$$

Here we use the probabilities with which different replacement events happen. We will sometimes refer to the two individuals in two neighbouring groups as the two focal individuals, and to their groups as the two focal groups.

If an individual event happens, the probability that any given group is chosen to host it is $\frac{1}{m}$. The probability that any individual within that group is chosen to be replaced is $\frac{1}{n}$. Therefore, for two given individuals in neighbouring groups, that adds up to $\frac{2}{m}\frac{1}{n}$. There are a few ways in which they can both not be affected. One of the other $m-2$ groups can be chosen, which happens with probability $\frac{m-2}{m}$; or one of the two neighbouring groups can be chosen, while some other individual is replaced, which happens with probability $\frac{2}{m}\frac{n-1}{n}$.

If a group event happens, then one of the two neighbouring groups can replace the other – which happens with probability $\frac{1}{m}\frac{m-1}{m}\frac{1}{2}$ – or the other can replace the one, which happens with the same probability. In both cases, q_0 is the relevant IBD probability. Also, a neighbour outside the focal pair of groups can replace one of the two in the focal pair, which again happens with a probability that is twice $\frac{1}{m}\frac{m-1}{m}\frac{1}{2}$. In this case, q_2 is the relevant IBD probability. Finally, with a probability of twice $\frac{1}{m^2}$, one of the focal groups replaces itself, in which case, q_1 is the relevant IBD probability. Nothing happens to the pair if a group is chosen to reproduce that is neither of the two groups within the focal pair, nor one of their direct neighbours, which happens with probability $\frac{m-4}{m}$. Also nothing happens if one of the neighbours of the focal pair is chosen, and they replace themselves ($\frac{1}{m^2}$) or their other neighbour ($\frac{1}{m}\frac{m-1}{m}\frac{1}{2}$). These probabilities together times 2 is $\frac{1}{m}\frac{m+1}{m^2}$. Finally, nothing happens if one of the two focal groups is chosen, and replaces a group outside the focal pair, which happens with probability $\frac{1}{m}\frac{m-1}{m^2}$.

With a migration event, every neighbouring pair is doing an exchange with probability $\frac{1}{m}$. This pair consists of both focal groups with probability $\frac{1}{m}$, and it is a pair that consists of one of the two focal groups and its neighbour on the other side with probability $\frac{2}{m}$. In the last case, the focal individual is chosen with probability $\frac{1}{n}$, and q_2 is the relevant IBD probability. In case the exchange is between the focal pair of groups, the two focal individuals themselves are chosen to switch with probability $\frac{1}{n^2}$, one of them is swapped with an individual that is not the other with probability $\frac{n-1}{n^2}$, and the other is swapped with an individual that is not the one with the same probability. In the first case, nothing changes with regard to the IBD probabilities, and in the latter two cases, q_s is the relevant IBD probability. Nothing happens at migration if one of the other $m-3$ pairs is chosen ($\frac{m-3}{m}$), a focal group and a neighbouring group outside the focal pair is chosen, but the focal individual is not chosen ($\frac{2}{m}\frac{n-1}{n}$), or the focal pair itself is chosen, but two individuals other

than the focal ones trade places ($\frac{1}{m}\frac{(n-1)^2}{n^2}$).

If we gather the terms with q_1 on the left-hand side, we get

$$\begin{aligned}\Bigg(1 - p\left(\frac{2}{m}\frac{1}{n}(1-u) + \frac{m-2}{m} + \frac{2}{m}\frac{n-1}{n}\right) - q\left(\frac{2}{m^2}(1-u) + \frac{m-2}{m}\right)\\ -r\left(\frac{1}{m}\frac{1}{n^2} + \frac{m-3}{m} + \frac{2}{m}\frac{n-1}{n} + \frac{1}{m}\frac{(n-1)^2}{n^2}\right)\Bigg) q_1\\ = q\frac{m-1}{m^2}(1-u)(q_0+q_2) + r\frac{2}{mn}\left(q_2 + \frac{n-1}{n}q_s\right)\end{aligned}$$

If we use the identity $q_0 = \frac{1}{n} + \frac{n-1}{n}q_s$, and multiply left and right by $\frac{m}{2}$, this can be rewritten as

$$\begin{aligned}\Bigg(\frac{m}{2} - \frac{p}{n}(1-u) - p\left(\frac{m-2}{2} + \frac{n-1}{n}\right) - \frac{q}{m}(1-u) - q\left(\frac{m-2}{2}\right)\\ -r\left(\frac{1}{2}\frac{1}{n^2} + \frac{m-3}{2} + \frac{n-1}{n} + \frac{1}{2}\frac{(n-1)^2}{n^2}\right)\Bigg) q_1\\ = \left(\frac{q}{2}\frac{m-1}{m}(1-u) + \frac{r}{n}\right)(q_0+q_2) - r\frac{1}{n^2}\end{aligned}$$

If we furthermore use that $p+q+r=1$, this can be further simplified to

$$\left(1 - (1-u)\left(\frac{p}{n} + \frac{q}{m}\right) - p\frac{n-1}{n} - r\left(\frac{n-1}{n}\right)^2\right) q_1 = \left((1-u)\frac{q}{2}\frac{m-1}{m} + \frac{r}{n}\right)(q_0+q_2) - \frac{r}{n^2} \tag{9}$$

4.1.2 $1 < i < m-1$

The recurrence relations for $1 < i < m-1$ are derived in a similar way. The differences with the recurrence relation for $i = 1$ arise because the two groups no longer are each other's neighbours, which means that the groups can no longer replace each other, nor can they exchange individuals.

$$\begin{aligned}q_i = \underbrace{p\frac{2}{m}\frac{1}{n}(1-u)q_i}_{\substack{\text{replaced in an}\\ \text{individual event}}} + \underbrace{q\left(\frac{1}{m}\frac{m-1}{m}(1-u)(q_{i-1}+q_{i+1}) + \frac{2}{m^2}(1-u)q_i\right)}_{\text{replaced in a group event}} + \underbrace{r\frac{2}{m}\frac{1}{n}(q_{i-1}+q_{i+1})}_{\substack{\text{replaced in a}\\ \text{migration event}}}\\ + \left(\underbrace{p\left(\frac{m-2}{m} + \frac{2}{m}\frac{n-1}{n}\right)}_{\substack{\text{no change through}\\ \text{individual events}}} + \underbrace{q\left(\frac{m-6}{m} + 2\frac{m+1}{m^2} + 2\frac{m-1}{m^2}\right)}_{\text{no change through group events}} + \underbrace{r\left(\frac{m-4}{m} + \frac{4}{m}\frac{n-1}{n}\right)}_{\substack{\text{no change through}\\ \text{migration events}}}\right) q_i\end{aligned}$$

If we gather the terms with q_i on the left-hand side, we get:

$$\left(1 - p\left(\frac{2}{m}\frac{1}{n}(1-u) + \frac{m-2}{m} + \frac{2}{m}\frac{n-1}{n}\right) - q\left(\frac{2}{m^2}(1-u) + \frac{m-2}{m}\right) - r\left(\frac{m-4}{m} + \frac{4}{m}\frac{n-1}{n}\right)\right) q_i$$
$$= \left(q\frac{m-1}{m^2}(1-u) + r\frac{2}{mn}\right)(q_{i-1} + q_{i+1})$$

If we multiply left and right by $\frac{m}{2}$, and use $p + q + r = 1$, this can be rewritten as

$$\left(\frac{p}{n} + q + \frac{2r}{n} - (1-u)\left(\frac{p}{n} + \frac{q}{m}\right)\right) q_i = \left((1-u)\frac{q}{2}\frac{m-1}{m} + \frac{r}{n}\right)(q_{i-1} + q_{i+1}) \tag{10}$$

4.1.3 $i = 0$

To derive q_s – and therewith q_0 – we will use the recurrence relation concerning the IBD probabilities for two individuals within the same group.

$$q_s = p\frac{1}{m}\frac{2}{n^2}(1-u) + p\left(\frac{1}{m}\frac{2(n-1)}{n^2}(1-u) + \frac{1}{m}\frac{n-2}{n} + \frac{m-1}{m}\right) q_s + q\left(\frac{1}{m^2} + \frac{m-1}{m^2}\right)(1-u)^2 q_s$$
$$+q\left(\frac{m-3}{m} + \frac{m+1}{m^2} + \frac{m-1}{m^2}\right) q_s + r\frac{4}{mn} q_1 + r\left(\frac{m-2}{m} + \frac{2(n-2)}{mn}\right) q_s$$

The relevant probabilities in case of an individual event are $\frac{1}{m}\frac{2}{n^2}$ for the focal group being chosen and one focal individual replacing the other, or vice versa; $\frac{1}{m}\frac{2(n-1)}{n^2}$ for the focal group being chosen, and any individual other than the other focal one replacing one of the focal ones (including herself), or vice versa; $\frac{1}{m}\frac{n-2}{n}$ for the focal group being chosen, and one of the other individuals being replaced; and $\frac{m-1}{m}$ for a different group being chosen.

In case of a group event, the relevant probabilities are $\frac{1}{m^2}$ for the focal group replacing itself; $\frac{m-1}{m^2}$ for the focal group being replaced by a neighbouring group; $\frac{m-3}{m}$ for a group being chosen to reproduce that is not the focal group nor a neighbour; $\frac{m+1}{m^2}$ for a neighbouring group being chosen to reproduce, and replacing itself or it's other neighbour; and $\frac{m-1}{m^2}$ for the focal group being chosen to reproduce, and replacing a neighbouring group and not itself.

In case of a migration event, the relevant probabilities are $\frac{4}{mn}$ for the focal group to exchange a member with the neighbouring group on the right or on the left, and one of the two focal individuals being chosen; $\frac{2(n-2)}{mn}$ for the exchange happening between the focal group and any of the two neighbouring groups, and neither of the two focal individuals being replaced; and $\frac{m-2}{m}$ for the exchange happening in any of the other $m-2$ pairs.

The equation can be rewritten as

$$q_s = p\frac{1}{m}\frac{2}{n^2}(1-u) + p\left(1 - \frac{2}{mn^2} + \frac{2}{mn}\left(1-\frac{1}{n}\right)(-u)\right)q_s + q\left(\frac{1}{m}\right)(1-u)^2 q_s$$
$$+q\left(\frac{m-1}{m}\right)q_s + r\frac{4}{mn}q_1 + r\left(1-\frac{4}{mn}\right)q_s$$

Expressing q_1 as a function of q_s, this becomes

$$q_1 = \frac{mn}{4r}\left(1 - p + p\frac{2}{mn}\left(u + \frac{1-u}{n}\right) - q\left(\frac{m-1}{m} + \frac{(1-u)^2}{m}\right) - r + r\frac{4}{mn}\right)q_s - \frac{p}{r}\frac{1-u}{2n}$$

Finally, we use $p+q+r=1$ to write

$$q_1 = \frac{mn}{4r}\left(p\frac{2}{mn}\left(u + \frac{1-u}{n}\right) + q\frac{1}{m} - q\frac{(1-u)^2}{m} + r\frac{4}{mn}\right)q_s - \frac{p}{r}\frac{1-u}{2n} \tag{11}$$

4.1.4 Solving the system

Suppose, for $1 \le i \le m-1$, q_i has the following form:

$$q_i = s_i q_1 \tag{12}$$

where $s_1 = s_{m-1} = 1$, and $\lim_{u\to 0} s_i = 1$ for $1 < i < m-1$, since all IBD probabilities approach 1 in the limit of no mutation. If we rewrite Equation (10) with the assumption in Equation (12), we get the following equality:

$$\left(\frac{p}{n} + q + \frac{2r}{n} - (1-u)\left(\frac{p}{n} + \frac{q}{m}\right)\right)s_i q_1 = \left((1-u)\frac{q}{2}\frac{m-1}{m} + \frac{r}{n}\right)(s_{i-1} + s_{i+1})q_1$$

Assuming that $q_1 \neq 0$, this is also:

$$\left(\frac{p}{n} + q + \frac{2r}{n} - (1-u)\left(\frac{p}{n} + \frac{q}{m}\right)\right)s_i = \left((1-u)\frac{q}{2}\frac{m-1}{m} + \frac{r}{n}\right)(s_{i-1} + s_{i+1}) \tag{13}$$

Since $q_i \to 1$ for all i as $u \downarrow 0$, we cannot directly use Equation (7) to calculate relatedness, as both the numerator and the denominator approach zero. Therefore, we will apply L'Hôpital's rule and calculate relatednesses as:

$$r_i = \frac{q_i' - \bar{q}'}{-\bar{q}'} \tag{14}$$

Here the derivatives are taken with respect to u, and evaluated in the limit of $u \downarrow 0$. In order to determine q_i' and $\bar{q}'$, we want to find s_i' by taking derivatives on both sides of Equation (13).

$$\left(\frac{p}{n} + q + \frac{2r}{n} - (1-u)\left(\frac{p}{n} + \frac{q}{m}\right)\right) s_i' + \left(\frac{p}{n} + \frac{q}{m}\right) s_i = \left((1-u)\frac{q}{2}\frac{m-1}{m} + \frac{r}{n}\right)(s_{i-1}' + s_{i+1}') - \frac{q}{2}\frac{m-1}{m}(s_{i-1} + s_{i+1})$$

If we evaluate this in the limit of $u \downarrow 0$, then we can also use that $s_i = 1$ for all i in that limit.

$$\left(q\frac{m-1}{m} + \frac{2r}{n}\right) s_i' + \frac{p}{n} + \frac{q}{m} = \left(\frac{q}{2}\frac{m-1}{m} + \frac{r}{n}\right)(s_{i-1}' + s_{i+1}') - q\frac{m-1}{m}$$

This can be reorganized as follows:

$$s_i' - \frac{s_{i-1}' + s_{i+1}'}{2} = -\frac{\frac{p}{n} + q}{q\frac{m-1}{m} + r\frac{2}{n}} \tag{15}$$

Summing both sides of the above equation for $2 \leq i \leq m-2$ yields

$$\sum_{i=2}^{m-2} s_i' - \frac{1}{2}\sum_{i=2}^{m-2}(s_{i-1}' + s_{i+1}') = -\sum_{i=2}^{m-2} \frac{\frac{p}{n} + q}{q\frac{m-1}{m} + r\frac{2}{n}}$$

$$\sum_{i=2}^{m-2} s_i' - \frac{1}{2}(s_1' + s_{m-1}' + s_2' + s_{m-2}') - \frac{1}{2}\sum_{i=3}^{m-3} 2s_i' = -(m-3)\frac{\frac{p}{n} + q}{q\frac{m-1}{m} + r\frac{2}{n}}$$

$$s_2' + s_{m-2}' - \frac{1}{2}(s_2' + s_{m-2}') = -(m-3)\frac{\frac{p}{n} + q}{q\frac{m-1}{m} + r\frac{2}{n}}$$

$$s_2' = -(m-3)\frac{\frac{p}{n} + q}{q\frac{m-1}{m} + r\frac{2}{n}} \tag{16}$$

where we used the identity $s_1' = s_{m-1}' = 0$ – since s_1 and s_{m-1} are constant – and $s_2' = s_{m-2}'$ – in the third and the fourth lines, respectively. Using the final equation above and the identity $s_1' = 0$, we can derive the limit values for all s_i' as given below:

$$s_i' = -(i-1)(m-i-1)\frac{\frac{p}{n} + q}{q\frac{m-1}{m} + r\frac{2}{n}} \tag{17}$$

for $2 \leq i \leq m-2$.

Before we can plug these into the relatedness formula, it will be helpful to write $\bar{q}'$ differently. Since $\bar{q} = \frac{1}{m}\sum_{i=0}^{m-1} q_i$, we can take the derivatives of all the terms separately.

$$\bar{q}' = \frac{1}{m}\left(q_0' + q_1' + q_{m-1}' + \sum_{i=2}^{m-2} q_i\right) = \frac{1}{m}\left(q_0' + q_1' + q_{m-1}' + \sum_{i=2}^{m-2}(s_i' q_1 + s_i q_1')\right)$$

Now we can use that $q_1 = 1$ and $s_i = 1$ in the limit of $u \downarrow 0$ for all i, that $q_1' = q_{m-1}'$, and that $s_1' = s_{m-1}' = 0$ (because $s_1 = s_{m-1} = 1$ regardless of u) when rewriting $\bar{q}'$.

$$\begin{aligned}\bar{q}' &= \frac{1}{m}\left(q_0' + q_1' + q_{m-1}' + \sum_{i=2}^{m-2}(s_i' + q_1')\right) = \frac{1}{m}\left(q_0' + (m-1)q_1' + \sum_{i=2}^{m-2} s_i'\right) \\ &= \frac{1}{m}\left(q_0' + (m-1)q_1' + \sum_{i=1}^{m-1} s_i'\right)\end{aligned}$$

We can now plug this in Equation (7) to get

$$r_0 = \frac{\frac{m-1}{m}(q_0' - q_1') - \frac{1}{m}\sum_{i=1}^{m-1} s_i'}{-\frac{1}{m}\left(q_0' + (m-1)q_1' + \sum_{i=1}^{m-1} s_i'\right)} = \frac{(m-1)(q_1' - q_0') + \sum_{i=1}^{m-1} s_i'}{q_0' + (m-1)q_1' + \sum_{i=1}^{m-1} s_i'} \tag{18}$$

$$r_1 = \frac{-\frac{1}{m}(q_0' - q_1') - \frac{1}{m}\sum_{i=1}^{m-1} s_i'}{-\frac{1}{m}\left(q_0' + (m-1)q_1' + \sum_{i=1}^{m-1} s_i'\right)} = \frac{-(q_1' - q_0') + \sum_{i=1}^{m-1} s_i'}{q_0' + (m-1)q_1' + \sum_{i=1}^{m-1} s_i'} \tag{19}$$

$$r_i = \frac{s_i' - \frac{1}{m}(q_0' - q_1') - \frac{1}{m}\sum_{i=1}^{m-1} s_i'}{-\frac{1}{m}\left(q_0' + (m-1)q_1' + \sum_{i=1}^{m-1} s_i'\right)} = r_1 - \frac{m s_i'}{q_0' + (m-1)q_1' + \sum_{i=1}^{m-1} s_i'} \tag{20}$$

where

$$\sum_{i=1}^{m-1} s_i' = -\frac{(m-1)(m-2)(m-3)}{6}\frac{\frac{p}{n} + q}{q\frac{m-1}{m} + r\frac{2}{n}} \tag{21}$$

In order to have a formula for relatedness that depends only on the parameters of the model (p, q, r, m and n), we still need to express q_0' and q_1' in terms of these parameters. In order to be able to do that, we will first express q_s' in terms of those parameters.

Step 1 is to take the first derivative with respect to u on both sides of Equation (9), and evaluate them at $u = 0$.

$$\left(1-p-\frac{q}{m}-r\left(\frac{n-1}{n}\right)^2\right)q_1'+\left(\frac{p}{n}+\frac{q}{m}\right)q_1=\left(\frac{q}{2}\frac{m-1}{m}+\frac{r}{n}\right)(q_0'+q_2')-\left(\frac{q}{2}\frac{m-1}{m}\right)(q_0+q_2)$$

At $u=0$, $q_0=q_1=q_2=1$, and hence

$$\left(1-p-\frac{q}{m}-r\left(\frac{n-1}{n}\right)^2\right)q_1'+\frac{p}{n}=\left(\frac{q}{2}\frac{m-1}{m}+\frac{r}{n}\right)(q_0'+q_2')-q$$

Also $q_0'=\frac{n-1}{n}q_s'$ and $q_2'=s_2'q_1+s_2q_1'$, and hence, with $q_2=q_1=1$ and $s_2=1$, at $u=0$, the expresion above becomes

$$\left(1-p-\frac{q}{m}-r\left(\frac{n-1}{n}\right)^2\right)q_1'+\frac{p}{n}=\left(\frac{q}{2}\frac{m-1}{m}+\frac{r}{n}\right)\left(\frac{n-1}{n}q_s'+s_2'+q_1'\right)-q$$

or

$$\left(1-p-\frac{q}{2}\frac{m+1}{m}-r\frac{(n-1)^2+n}{n^2}\right)q_1'+\frac{p}{n}=\left(\frac{q}{2}\frac{m-1}{m}+\frac{r}{n}\right)\left(\frac{n-1}{n}q_s'+s_2'\right)-q$$

Step 2 is to take the first derivative with respect to u in Equation (11)

$$q_1'=\frac{mn}{4r}\left(p\frac{2}{mn}\left(u+\frac{1-u}{n}\right)+q\frac{1}{m}-q\frac{(1-u)^2}{m}+r\frac{4}{mn}\right)q_s'+\frac{mn}{4r}\left(p\frac{2}{mn}\left(1-\frac{1}{n}\right)+2q\frac{(1-u)}{m}\right)q_s+\frac{p}{r}\frac{1}{2n}$$

Evaluated at $u=0$, where also $q_s=1$, this is

$$\begin{aligned}q_1'&=\left(\frac{p}{r}\frac{1}{2n}+1\right)q_s'+\frac{mn}{4r}\left(p\frac{2}{mn}\left(1-\frac{1}{n}\right)+2q\frac{1}{m}\right)+\frac{p}{r}\frac{1}{2n}=\left(\frac{p}{r}\frac{1}{2n}+1\right)q_s'+\frac{p}{2r}\left(1-\frac{1}{n}\right)+\frac{qn}{2r}+\frac{p}{r}\frac{1}{2n}\\&=\left(\frac{p}{r}\frac{1}{2n}+1\right)q_s'+\frac{p}{2r}+\frac{qn}{2r}\end{aligned}$$

If we combine these two steps, we get

$$\left(1-p-\frac{q}{2}\frac{m+1}{m}-r\frac{(n-1)^2+n}{n^2}\right)\left(\left(\frac{p}{r}\frac{1}{2n}+1\right)q_s'+\frac{p}{2r}+\frac{qn}{2r}\right)+\frac{p}{n}=\left(\frac{q}{2}\frac{m-1}{m}+\frac{r}{n}\right)(\frac{n-1}{n}q_s'+s_2')-q$$

or

$$\left(1-p-\frac{q}{2}\frac{m+1}{m}-r\frac{(n-1)^2+n}{n^2}\right)\left(\frac{p}{r}\frac{1}{2n}+1\right)q_s'-\left(\frac{q}{2}\frac{m-1}{m}+\frac{r}{n}\right)\frac{n-1}{n}q_s'$$
$$=\left(\frac{q}{2}\frac{m-1}{m}+\frac{r}{n}\right)s_2'-\left(1-p-\frac{q}{2}\frac{m+1}{m}-r\frac{(n-1)^2+n}{n^2}\right)\left(\frac{p}{2r}+\frac{qn}{2r}\right)-q-\frac{p}{n}$$

or

$$\left(1-p-\frac{q}{2}\frac{m+1}{m}-r\frac{(n-1)^2+n}{n^2}\right)\left(\frac{p}{r}\frac{1}{n}+2\right)q_s'-\left(q\frac{m-1}{m}+\frac{2r}{n}\right)\frac{n-1}{n}q_s'$$
$$=\left(q\frac{m-1}{m}+\frac{2r}{n}\right)s_2'-\left(\frac{n}{r}-\frac{pn}{r}-\frac{qn}{2r}\frac{m+1}{m}-\frac{(n-1)^2+n}{n}\right)\left(\frac{p}{n}+q\right)-2\left(\frac{p}{n}+q\right)$$

which gives

$$q_s'=\frac{\left(q\frac{m-1}{m}+\frac{2r}{n}\right)s_2'-\left(\frac{p}{n}+q\right)\left(\frac{n}{r}-\frac{pn}{r}-\frac{qn}{2r}\frac{m+1}{m}-\frac{(n-1)^2}{n}+1\right)}{\left(1-p-\frac{q}{2}\frac{m+1}{m}-r\frac{(n-1)^2+n}{n^2}\right)\left(\frac{p}{r}\frac{1}{n}+2\right)-\left(q\frac{m-1}{m}+\frac{2r}{n}\right)\frac{n-1}{n}}$$
$$=\frac{\left(q\frac{m-1}{m}+\frac{2r}{n}\right)s_2'-\left(\frac{p}{n}+q\right)\left(3-\frac{1}{n}-\frac{qn}{2r}\frac{m+1}{m}+\frac{n}{r}-\frac{pn}{r}-n\right)}{\left(1-p-\frac{q}{2}\frac{m+1}{m}-r\frac{(n-1)^2+n}{n^2}\right)\left(\frac{p}{r}\frac{1}{n}+2\right)-\left(q\frac{m-1}{m}+\frac{2r}{n}\right)\frac{n-1}{n}}$$

We can simplify this using the formula for s_2' from Equation (16), repeated below.

$$s_2'=-(m-3)\frac{\frac{p}{n}+q}{q\frac{m-1}{m}+r\frac{2}{n}}$$

If we look at the first term in the numerator in Equation (22), we see that the coefficient of s_2' is equal to the denominator of s_2', so we can rewrite the formula for q_s' as follows:

$$q_s'=\frac{-(m-3)\left(\frac{p}{n}+q\right)-\left(\frac{p}{n}+q\right)\left(3-\frac{1}{n}-\frac{qn}{2r}\frac{m+1}{m}+\frac{n}{r}-\frac{pn}{r}-n\right)}{\left(1-p-\frac{q}{2}\frac{m+1}{m}-r\frac{(n-1)^2+n}{n^2}\right)\left(\frac{p}{r}\frac{1}{n}+2\right)-\left(q\frac{m-1}{m}+\frac{2r}{n}\right)\frac{n-1}{n}}$$
$$=\frac{-\left(\frac{p}{n}+q\right)\left(m-\frac{1}{n}-\frac{qn}{2r}\frac{m+1}{m}+\frac{n}{r}-\frac{pn}{r}-n\right)}{\left(1-p-\frac{q}{2}\frac{m+1}{m}-r\frac{(n-1)^2+n}{n^2}\right)\left(\frac{p}{r}\frac{1}{n}+2\right)-\left(q\frac{m-1}{m}+\frac{2r}{n}\right)\frac{n-1}{n}}$$

We can further rewrite the numerator,

$$
\begin{aligned}
q'_s &= \frac{-\left(\frac{p}{n}+q\right)\left(m-\frac{1}{n}-\frac{qn}{2r}\frac{m+1}{m}+n\left(\frac{1-p-r}{r}\right)\right)}{\left(1-p-\frac{q}{2}\frac{m+1}{m}-r\frac{(n-1)^2+n}{n^2}\right)\left(\frac{p}{r}\frac{1}{n}+2\right)-\left(q\frac{m-1}{m}+\frac{2r}{n}\right)\frac{n-1}{n}} \\
&= \frac{-\left(\frac{p}{n}+q\right)\left(m-\frac{1}{n}-\frac{qn}{2r}\frac{m+1}{m}+n\frac{q}{r}\right)}{\left(1-p-\frac{q}{2}\frac{m+1}{m}-r\frac{(n-1)^2+n}{n^2}\right)\left(\frac{p}{r}\frac{1}{n}+2\right)-\left(q\frac{m-1}{m}+\frac{2r}{n}\right)\frac{n-1}{n}} \\
&= \frac{-\left(\frac{p}{n}+q\right)\left(m-\frac{1}{n}+\frac{qn}{2r}\frac{m-1}{m}\right)}{\left(1-p-\frac{q}{2}\frac{m+1}{m}-r\frac{(n-1)^2+n}{n^2}\right)\left(\frac{p}{r}\frac{1}{n}+2\right)-\left(q\frac{m-1}{m}+\frac{2r}{n}\right)\frac{n-1}{n}}
\end{aligned}
$$

where we used $p+q+r=1$ in the second line.

Then, we can rewrite the denominator,

$$
\begin{aligned}
q'_s &= \frac{-\left(\frac{p}{n}+q\right)\left(m-\frac{1}{n}+\frac{qn}{2r}\frac{m-1}{m}\right)}{\left(1-p-\frac{q}{2}\frac{m+1}{m}-r\frac{n^2-2n+1+n}{n^2}\right)\left(\frac{p}{r}\frac{1}{n}+2\right)-\left(q\frac{m-1}{m}+\frac{2r}{n}\right)\frac{n-1}{n}} \\
&= \frac{-\left(\frac{p}{n}+q\right)\left(m-\frac{1}{n}+\frac{qn}{2r}\frac{m-1}{m}\right)}{\left(1-p-\frac{q}{2}\frac{m+1}{m}-r+r\frac{n-1}{n^2}\right)\left(\frac{p}{r}\frac{1}{n}+2\right)-\left(q\frac{m-1}{m}+\frac{2r}{n}\right)\frac{n-1}{n}} \\
&= \frac{-\left(\frac{p}{n}+q\right)\left(m-\frac{1}{n}+\frac{qn}{2r}\frac{m-1}{m}\right)}{\left(q-\frac{q}{2}\frac{m+1}{m}+r\frac{n-1}{n^2}\right)\left(\frac{p}{r}\frac{1}{n}+2\right)-\left(q\frac{m-1}{m}+\frac{2r}{n}\right)\frac{n-1}{n}} \\
&= \frac{-\left(\frac{p}{n}+q\right)\left(m-\frac{1}{n}+\frac{qn}{2r}\frac{m-1}{m}\right)}{\left(\frac{q}{2}\frac{m-1}{m}+r\frac{n-1}{n^2}\right)\left(\frac{p}{r}\frac{1}{n}+2\right)-\left(q\frac{m-1}{m}+\frac{2r}{n}\right)\frac{n-1}{n}} \\
&= \frac{-\left(\frac{p}{n}+q\right)\left(m-\frac{1}{n}+\frac{qn}{2r}\frac{m-1}{m}\right)}{\frac{pq}{2rn}\frac{m-1}{m}+q\frac{m-1}{m}+p\frac{n-1}{n^3}+r\frac{2(n-1)}{n^2}-\left(q\frac{m-1}{m}+\frac{2r}{n}\right)\frac{n-1}{n}}
\end{aligned}
$$

after which we arrive at

$$
q'_s = \frac{-\left(\frac{p}{n}+q\right)\left(m-\frac{1}{n}+\frac{qn}{2r}\frac{m-1}{m}\right)}{\frac{pq}{r}\frac{1}{2n}\frac{m-1}{m}+q\frac{1}{n}\frac{m-1}{m}+p\frac{n-1}{n^3}} \tag{22}
$$

Equation (8) moreover implies

$$
q'_0 = \frac{n-1}{n}q'_s \tag{23}
$$

which links q'_s to q'_0, and Step 2 above gave us

$$
q'_1 = \left(\frac{p}{r}\frac{1}{2n}+1\right)q'_s + \frac{p+nq}{2r} \tag{24}
$$

which links q'_s to q'_1. This implies that we have everything we need to complete Equations (18), (19) and (20)

for the BD process.

4.2 Shift

In the Shift process, an individual can be replaced if:

- An individual event happens, the group the individual is in is chosen to host it, and then within the group, she is chosen to be replaced.
- A group event happens, and a neighbouring group, or its offspring group, pushes the individual's group one position away from where it was, or replaces it. Probabilities for those events are also derived in Allen and Nowak (2012) for the Shift process, where all positions are occupied by individuals instead of groups.
- A migration event occurs, the individual's group is chosen to be one of the groups in which the migration takes place, and the individual is chosen to be swapped.

Combining these events, we can derive the recurrence relations for the stationary IBD probabilities.

4.2.1 $i = 1$

For $i = 1$, we have the following recurrence relation:

$$q_1 = \underbrace{p\frac{2}{m}\frac{1}{n}(1-u)q_1}_{\substack{\text{replaced in an}\\ \text{individual event}}} + \underbrace{q\left(\frac{(m-1)(1-u)}{m^2}q_0 + \frac{1-u}{m}q_1 + \frac{(m-2+(1-u))}{m^2}q_2\right)}_{\text{replaced in a group event}} + \underbrace{r\frac{1}{m}\frac{1}{n}\left(2q_2 + \frac{2(n-1)}{n}q_s + \frac{1}{n}q_1\right)}_{\text{replaced in a migration event}}$$
$$+\left(\underbrace{p\left(\frac{m-2}{m} + \frac{2}{m}\frac{n-1}{n}\right)}_{\substack{\text{no change through}\\ \text{individual events}}} + \underbrace{q\left(\frac{(m-1)(m-2)}{m^2}\right)}_{\text{no change through group events}} + \underbrace{r\left(\frac{m-3}{m} + \frac{2}{m}\frac{n-1}{n} + \frac{1}{m}\frac{(n-1)^2}{n^2}\right)}_{\text{no change through migration events}}\right)q_1$$

The probabilities for individual and migration events are the same as in BD. The probabilities for group events are different. To get the probabilities for group events right, it is important, in the face of equivalent ways to define this update rule, to have an unambiguous rule for who ends up at which location after a group reproduction event. The reproducing group always stays put. If it is also chosen to die, its offspring group takes its place and no group moves. If not, then with probability one half, the offspring group occupies the position to the left of the parent group, and every group in between the reproducing and the dying group moves in the same direction. Also with probability one half, the offspring group occupies the position on the right, and every group in between the reproducing and the dying group moves in that direction.

Now, for a given pair of neighbouring positions, the group on the left replaces the group on the right, if the left one is chosen to reproduce, not chosen to also die, and reproduces on the right. The right one replaces the left one if the right one is chosen to reproduce, not chosen to also die, and reproduces to the left. Both events happen with probability $\frac{1}{2}\frac{m-1}{m^2}$, and after this, two randomly chosen members of the neighbouring groups are IBD with probability $(1-u)q_0$.

The left one replaces itself with probability $\frac{1}{m^2}$. The right one too. After this, two randomly chosen members of the neighbouring groups are IBD with probability $(1-u)q_1$.

The group to the left of the left one reproduces to the right and pushes the left group to the right position if the group to the left of the left one is chosen to reproduce, the left group and the group to the left of the left one are both not chosen to die, and the reproducing group reproduces to the right. This happens with probability $\frac{1}{2}\frac{m-2}{m^2}$. The mirror image of that happens with the same probability. After this, two randomly chosen members of the neighbouring groups are IBD with probability $(1-u)q_1$.

These probabilities and the probabilities with which they replace themselves add up to $\frac{1}{m}$, and we have seen that, for both, the IBD probability is $(1-u)q_1$.

The left one is replaced by the group to the left of it, if the left one is chosen to die, any group other than the left one and its left neighbour is chosen to reproduce, and the reproducing group reproduces to the right. The right one is replaced by the group to the right of it, if the right one is chosen to die, any group other than the right one and its right neighbour is chosen to reproduce, and the reproducing group reproduces to the left. Both events happen with probability $\frac{1}{2}\frac{m-2}{m^2}$, and after this, two randomly chosen members of the neighbouring groups are IBD with probability q_2.

The left one is replaced by the offspring of the group to the left of it, if the left one is chosen to die, its left neighbour is chosen to reproduce, and it reproduces to the right. The right one is replaced by the offspring of the group to the right of it, if the right one is chosen to die, its right neighbour is chosen to reproduce, and it reproduces to the left. Both events happen with probability $\frac{1}{2}\frac{1}{m^2}$, and after this, two randomly chosen members of the neighbouring groups are IBD with probability $(1-u)q_2$.

After all other events, the groups at the two given neighbouring locations are both not the offspring group, and were neighbouring groups in the period before the group event, too.

All of those group event probabilities can also be found in Allen and Nowak (2012). The only differences are that we derive them in a forward looking way, while they do it in a backward looking way, and, since we may have more than one individual at any site, we do not have $q_0 = 1$.

If we gather all terms with q_1 on the left hand side, we get

$$\begin{aligned}\Bigg(1 - p\left(\frac{2}{m}\frac{1}{n}(1-u) + \frac{m-2}{m} + \frac{2}{m}\frac{n-1}{n}\right) - q\left(\frac{1-u}{m} + \frac{(m-1)(m-2)}{m^2}\right) \\ -r\left(\frac{1}{m}\frac{1}{n^2} + \frac{m-3}{m} + \frac{2}{m}\frac{n-1}{n} + \frac{1}{m}\frac{(n-1)^2}{n^2}\right)\Bigg) q_1 = \\ q\left(\frac{(m-1)(1-u)}{m^2}q_0 + \frac{(m-2+(1-u))}{m^2}q_2\right) + r\frac{1}{m}\frac{1}{n}\left(2q_2 + \frac{2(n-1)}{n}q_s\right)\end{aligned}$$

If we use the identity $q_0 = \frac{1+(n-1)q_s}{n}$, and multiply left and right by m, this can be rewritten as

$$\left(m - p\left(m - \frac{2}{n}u\right) - q\left(m - \frac{2m-2}{m} - u\right) - r\left(m - \frac{4n-2}{n^2}\right)\right) q_1 =$$
$$\left(q\frac{m-1}{m} + r\frac{2}{n}\right)(q_0 + q_2) - q\frac{u}{m}((m-1)q_0 + q_2) - r\frac{2}{n^2}$$

If we furthermore use that $p + q + r = 1$, this can be further simplified to

$$\left(q\frac{2(m-1)}{m} + r\frac{2(2n-1)}{n^2} + u\left(p\frac{2}{n} + q\right)\right) q_1 = \left(q\frac{m-1}{m} + r\frac{2}{n}\right)(q_0 + q_2) - q\frac{u}{m}((m-1)q_0 + q_2) - r\frac{2}{n^2} \tag{25}$$

4.2.2 $1 < i < m - 1$

The recurrence relations for $1 < i < m - 1$ are derived in a similar way. The differences with the recurrence relation for $i = 1$ arise because the two groups no longer are each other's neighbours, which means that the groups can no longer replace each other, nor can they swap individuals.

$$q_i = \underbrace{p\frac{2}{m}\frac{1}{n}(1-u)q_i}_{\substack{\text{replaced in an}\\ \text{individual event}}} + \underbrace{q\left(\frac{(m-i)(i-1+(1-u))}{m^2}q_{i-1} + \frac{1-u}{m}q_i + \frac{i(m-i-1+(1-u))}{m^2}q_{i+1}\right)}_{\text{replaced in a group event}} + \underbrace{r\frac{2}{m}\frac{1}{n}(q_{i-1} + q_{i+1})}_{\substack{\text{replaced in a}\\ \text{migration event}}}$$
$$+ \left(\underbrace{p\left(\frac{m-2}{m} + \frac{2}{m}\frac{n-1}{n}\right)}_{\substack{\text{no change through}\\ \text{individual events}}} + \underbrace{q\left(\frac{(m-i)(m-i-1) + i(i-1)}{m^2}\right)}_{\text{no change through group events}} + \underbrace{r\left(\frac{m-4}{m} + \frac{4}{m}\frac{n-1}{n}\right)}_{\substack{\text{no change through}\\ \text{migration events}}}\right) q_i$$

If we gather the terms with q_i on the left-hand side, we get

$$\left(1 - p\left(\frac{2}{m}\frac{1}{n}(1-u) + \frac{m-2}{m} + \frac{2}{m}\frac{n-1}{n}\right) - q\frac{(m-i)(m-i-u) + i(i-u)}{m^2} - r\left(\frac{m-4}{m} + \frac{4}{m}\frac{n-1}{n}\right)\right) q_i$$
$$= \left(q\frac{(m-i)(i-u)}{m^2} + r\frac{2}{m}\frac{1}{n}\right) q_{i-1} + \left(q\frac{i(m-i-u)}{m^2} + r\frac{2}{m}\frac{1}{n}\right) q_{i+1}$$

If we multiply both sides with m, and use $p + q + r = 1$ again, we can rewrite this as

$$\left(q\frac{2i(m-i)}{m} + r\frac{4}{n} + u\left(p\frac{2}{n} + q\right)\right) q_i = \left(q\frac{i(m-i)}{m} + r\frac{2}{n}\right)(q_{i-1} + q_{i+1}) - q\frac{u}{m}((m-i)q_{i-1} + iq_{i+1}) \tag{26}$$

4.2.3 $i = 0$

To derive q_s – and therewith q_0 – we use the recurrence relation concerning the IBD probabilities for two individuals from one and the same group.

$$q_s = p\frac{1}{m}\frac{2}{n^2}(1-u) + p\left(\frac{1}{m}\frac{2(n-1)}{n^2}(1-u) + \frac{1}{m}\frac{n-2}{n} + \frac{m-1}{m}\right)q_s + q\left(\frac{(1-u)^2}{m} + \frac{m-1}{m}\right)q_s$$
$$+r\frac{4}{mn}q_1 + r\left(\frac{m-2}{m} + \frac{2(n-2)}{mn}\right)q_s$$

Again, the probabilities for individual and migration events are the same as in BD. If a group event happens, then any given group reproduces and replaces itself with probability $\frac{1}{m^2}$, is replaced by its left neighbour with probability $\frac{1}{2}\frac{m-1}{m^2}$, and by its right neighbour with the same probability. That adds up to $\frac{1}{m}$, and the IBD probability dilutes to $(1-u)^2 q_s$ in all of these cases. In all other cases, it remains q_s.

Expressing q_1 as a function of q_s, this becomes

$$q_1 = \frac{mn}{4r}\left(1 - p + p\frac{2}{mn}\left(u + \frac{1-u}{n}\right) - q\left(\frac{(1-u)^2}{m} + \frac{m-1}{m}\right) - r + r\frac{4}{mn}\right)q_s - \frac{p}{r}\frac{1-u}{2n}$$

Finally, we use $p + q + r = 1$ to write

$$q_1 = \frac{mn}{4r}\left(p\frac{2}{mn}\left(u + \frac{1-u}{n}\right) + q\frac{1}{m} - q\frac{(1-u)^2}{m} + r\frac{4}{mn}\right)q_s - \frac{p}{r}\frac{1-u}{2n} \quad (27)$$

This turns out to be the same equation for Shift as for BD.

4.2.4 Solving the system

Suppose again that q_i has the following form for $1 \leq i \leq m-1$:

$$q_i = s_i q_1$$

where $s_1 = s_{m-1} = 1$, and $\lim_{u\to 0} s_i = 1$ for $1 < i < m-1$. Then, we can rewrite Equation (26) as follows:

$$\left(q\frac{2i(m-i)}{m} + r\frac{4}{n} + u\left(p\frac{2}{n} + q\right)\right)s_i q_1 = \left(q\frac{i(m-i)}{m} + r\frac{2}{n}\right)(s_{i-1} + s_{i+1})q_1 - q\frac{u}{m}((m-i)s_{i-1} + i s_{i+1})q_1$$

Assuming that $q_1 \neq 0$, this is also:

$$\left(q\frac{2i(m-i)}{m}+r\frac{4}{n}+u\left(p\frac{2}{n}+q\right)\right)s_i=\left(q\frac{i(m-i)}{m}+r\frac{2}{n}\right)(s_{i-1}+s_{i+1})-q\frac{u}{m}((m-i)s_{i-1}+is_{i+1}) \quad (28)$$

Since $q_i \to 1$ for all i as $u \downarrow 0$, we cannot directly use Equation (7) to calculate relatedness, as both the numerator and the denominator approach zero. Therefore, we will apply L'Hôpital's rule and calculate relatednesses as:

$$r_i=\frac{q_i'-\bar{q}'}{-\bar{q}'}$$

Here the derivatives are taken with respect to u, and evaluated in the limit of $u \downarrow 0$. In order to determine q_i' and $\bar{q}'$, we want to find s_i' by taking derivatives with respect to u on both sides of Equation (28).

$$\left(q\frac{2i(m-i)}{m}+r\frac{4}{n}+u\left(p\frac{2}{n}+q\right)\right)s_i'+\left(p\frac{2}{n}+q\right)s_i=$$
$$\left(q\frac{i(m-i)}{m}+r\frac{2}{n}\right)(s_{i-1}'+s_{i+1}')-\frac{q}{m}((m-i)s_{i-1}+is_{i+1})-q\frac{u}{m}\left((m-i)s_{i-1}'+is_{i+1}'\right)$$

If we evaluate this in the limit of $u \downarrow 0$, then we can also use that $s_i = 1$ for all i in that limit.

$$\left(q\frac{2i(m-i)}{m}+r\frac{4}{n}\right)s_i'+\left(p\frac{2}{n}+q\right)=\left(q\frac{i(m-i)}{m}+r\frac{2}{n}\right)(s_{i-1}'+s_{i+1}')-q$$

This can be reorganized as follows

$$\left(q\frac{i(m-i)}{m}+r\frac{2}{n}\right)(2s_i'-(s_{i-1}'+s_{i+1}'))=-\left(p\frac{2}{n}+2q\right)$$

If we divide everything by 2 and by the first term, we get the following equation.

$$s_i'-\frac{s_{i-1}'+s_{i+1}'}{2}=-\frac{\frac{p}{n}+q}{q\frac{i(m-i)}{m}+r\frac{2}{n}} \quad (29)$$

We will call the right hand side of this equation $-\eta_i$. If we do so, and we sum both sides of the equation over $2 \le i \le m-2$, we get

$$\sum_{i=2}^{m-2}s_i'-\frac{1}{2}\sum_{i=2}^{m-2}(s_{i-1}'+s_{i+1}')=-\sum_{i=2}^{m-2}\eta_i$$

$$\sum_{i=2}^{m-2} s'_i - \frac{1}{2}(s'_1 + s'_{m-1} + s'_2 + s'_{m-2}) - \frac{1}{2}\sum_{i=3}^{m-3} 2s'_i = -\sum_{i=2}^{m-2} \eta_i$$

$$s'_2 + s'_{m-2} - \frac{1}{2}(s'_2 + s'_{m-2}) = -\sum_{i=2}^{m-2} \eta_i$$

$$s'_2 = -\sum_{i=2}^{m-2} \eta_i \tag{30}$$

which, together with Equation (29), implies that

$$s'_j = -(j-1)\sum_{i=2}^{m-2} \eta_i + 2\sum_{i=2}^{j-1}(j-i)\eta_i$$

The derivations of Equations (18), (19) and (20) in the previous subsection do not depend on the update process; they only depend on the assumption that $q_i = s_i q_1$ for $1 \le i \le m-1$. Here we make the same assumption for Shift. Hence, we can use the same equations for Shift as we did for BD. Because Equations (18), (19) and (20) also feature the sum of s'_j values, it will help to compute that sum as well. Since $s'_1 = s'_{m-1} = 0$, summing from $j = 2$ to $m-2$ gives the same answer as summing from $j = 1$ to $m-1$.

$$\sum_{j=1}^{m-1} s'_j = \sum_{j=2}^{m-2} s'_j = -\sum_{j=2}^{m-2}(j-1)\sum_{i=2}^{m-2} \eta_i + 2\sum_{j=2}^{m-2}\sum_{i=2}^{j-1}(j-i)\eta_i$$

The first part of the above summation can be calculated as follows

$$\sum_{j=2}^{m-2}(j-1)\sum_{i=2}^{m-2} \eta_i = (1+2+...+m-3)\sum_{i=2}^{m-2} \eta_i = \frac{(m-3)(m-2)}{2}\sum_{i=2}^{m-2} \eta_i \tag{31}$$

The second part of the summation can be calculated as follows

$$\sum_{j=2}^{m-2}\sum_{i=2}^{j-1}(j-i)\eta_i = \underbrace{\eta_2}_{j=3} + \underbrace{2\eta_2 + \eta_3}_{j=4} + \underbrace{3\eta_2 + 2\eta_3 + \eta_4}_{j=5} + ... + \underbrace{(m-4)\eta_2 + (m-5)\eta_3 + ... + 3\eta_{m-5} + 2\eta_{m-4} + \eta_{m-3}}_{j=m-2}$$

When we extend the summation in this way, we see that for every η_i, the first time it appears, it is multiplied by 1; the next time it is multiplied by 2; and so on; and the last point the same term appears, it is multiplied by $(m-2-i)$. Therefore, we can rewrite the above summation as follows

$$\sum_{j=2}^{m-2}\sum_{i=2}^{j-1}(j-i)\eta_i = \sum_{i=2}^{m-3}(1+2+...+m-2-i)\eta_i = \sum_{i=2}^{m-3}\frac{(m-2-i)(m-1-i)}{2}\eta_i \tag{32}$$

Now, if we combine the two parts found in (31) and (32), we see that

$$\begin{aligned}\sum_{j=2}^{m-2} s'_j &= -\sum_{j=2}^{m-2}(j-1)\sum_{i=2}^{m-2}\eta_i + 2\sum_{j=2}^{m-2}\sum_{i=2}^{j-1}(j-i)\eta_i \\ &= -\frac{(m-3)(m-2)}{2}\sum_{i=2}^{m-2}\eta_i + 2\sum_{i=2}^{m-3}\frac{(m-2-i)(m-1-i)}{2}\eta_i\end{aligned}$$

We can write the terms with η_2 and η_{m-2} separately, and use that $\eta_2 = \eta_{m-2}$:

$$\begin{aligned}\sum_{j=2}^{m-2} s'_j &= -\frac{(m-3)(m-2)}{2}(\eta_2+\eta_{m-2}) - \frac{(m-3)(m-2)}{2}\sum_{i=3}^{m-3}\eta_i + (m-4)(m-3)\eta_2 + 2\sum_{i=3}^{m-3}\frac{(m-2-i)(m-1-i)}{2}\eta_i \\ &= -(m-3)(m-2)\eta_2 - \frac{(m-3)(m-2)}{2}\sum_{i=3}^{m-3}\eta_i + (m-4)(m-3)\eta_2 + 2\sum_{i=3}^{m-3}\frac{(m-2-i)(m-1-i)}{2}\eta_i \\ &= -2(m-3)\eta_2 + \sum_{i=3}^{m-3}\left(2\frac{(m-2-i)(m-1-i)}{2} - \frac{(m-3)(m-2)}{2}\right)\eta_i\end{aligned}$$

Rearranging gives the formula below.

$$\sum_{j=1}^{m-1} s'_j = \sum_{j=2}^{m-2} s'_j = -2(m-3)\eta_2 + \sum_{i=3}^{m-3}\left(\frac{m^2-m-2-2i(2m-3)+2i^2}{2}\right)\eta_i \tag{33}$$

Unfortunately, there is no closed-form solution for the values s'_i and their sum. However, it is possible to find numerical solutions once we fix the population size.

In order to have a formula for relatedness that only depends on the parameters of the model (p, q, r, m and n), we still need to express q'_0 and q'_1 in terms of these parameters. In order to be able to do that, we will first express q'_s in terms of those parameters.

Step 1 is to take the first derivative with respect to u on both sides of Equation (25), and evaluate them at $u = 0$.

$$\left(q\frac{2(m-1)}{m} + r\frac{2(2n-1)}{n^2}\right)q'_1 + \left(p\frac{2}{n}+q\right)q_1 = \left(q\frac{m-1}{m}+r\frac{2}{n}\right)(q'_0+q'_2) - \frac{q}{m}((m-1)q_0+q_2)$$

At $u = 0$, also $q_0 = q_1 = q_2 = 1$, and hence

$$\left(q\frac{2(m-1)}{m} + r\frac{2(2n-1)}{n^2}\right) q_1' + \left(p\frac{2}{n} + 2q\right) = \left(q\frac{m-1}{m} + r\frac{2}{n}\right)(q_0' + q_2')$$

Also $q_0' = \frac{n-1}{n} q_s'$ and $q_2' = s_2' q_1 + s_2 q_1'$, and hence, with $q_2 = q_1 = 1$ and $s_2 = 1$ at $u = 0$, this is also

$$\left(q\frac{2(m-1)}{m} + r\frac{2(2n-1)}{n^2}\right) q_1' + \left(p\frac{2}{n} + 2q\right) = \left(q\frac{m-1}{m} + r\frac{2}{n}\right)\left(\frac{n-1}{n} q_s' + s_2' + q_1'\right)$$

or

$$\left(q\frac{m-1}{m} + r\frac{2(n-1)}{n^2}\right) q_1' + \left(p\frac{2}{n} + 2q\right) = \left(q\frac{m-1}{m} + r\frac{2}{n}\right)\left(\frac{n-1}{n} q_s' + s_2'\right)$$

Step 2 is to take the first derivative with respect to u in Equation (27)

$$q_1' = \frac{mn}{4r}\left(p\frac{2}{mn}\left(u + \frac{1-u}{n}\right) + q\frac{1}{m} - q\frac{(1-u)^2}{m} + r\frac{4}{mn}\right) q_s' + \frac{mn}{4r}\left(p\frac{2}{mn}\left(1 - \frac{1}{n}\right) + 2q\frac{(1-u)}{m}\right) q_s + \frac{p}{r}\frac{1}{2n}$$

Evaluated at $u = 0$, where also $q_s = 1$, this is

$$\begin{aligned} q_1' &= \left(\frac{p}{r}\frac{1}{2n} + 1\right) q_s' + \frac{mn}{4r}\left(p\frac{2}{mn}\left(1 - \frac{1}{n}\right) + 2q\frac{1}{m}\right) + \frac{p}{r}\frac{1}{2n} = \left(\frac{p}{r}\frac{1}{2n} + 1\right) q_s' + \frac{p}{2r}\left(1 - \frac{1}{n}\right) + \frac{qn}{2r} + \frac{p}{r}\frac{1}{2n} \\ &= \left(\frac{p}{r}\frac{1}{2n} + 1\right) q_s' + \frac{p}{2r} + \frac{qn}{2r} \end{aligned}$$

If we combine these two steps, we get

$$\left(q\frac{m-1}{m} + r\frac{2(n-1)}{n^2}\right)\left(\left(\frac{p}{r}\frac{1}{2n} + 1\right) q_s' + \frac{p}{2r} + \frac{qn}{2r}\right) + \left(p\frac{2}{n} + 2q\right) = \left(q\frac{m-1}{m} + r\frac{2}{n}\right)\left(\frac{n-1}{n} q_s' + s_2'\right)$$

or

$$\begin{gathered} \left(q\frac{m-1}{m} + r\frac{2(n-1)}{n^2}\right)\left(\frac{p}{r}\frac{1}{2n} + 1\right) q_s' - \left(q\frac{m-1}{m} + r\frac{2}{n}\right)\frac{n-1}{n} q_s' = \\ \left(q\frac{m-1}{m} + r\frac{2}{n}\right) s_2' - \left(q\frac{m-1}{m} + r\frac{2(n-1)}{n^2}\right)\left(\frac{p}{2r} + \frac{qn}{2r}\right) - \left(p\frac{2}{n} + 2q\right) \end{gathered}$$

or

$$\left(\frac{pq}{r}\frac{1}{2n}\frac{m-1}{m}+q\frac{m-1}{m}\frac{1}{n}+p\frac{n-1}{n^3}\right)q'_s =$$
$$\left(q\frac{m-1}{m}+r\frac{2}{n}\right)s'_2-\left(\frac{q}{r}\frac{n}{2}\frac{m-1}{m}+\frac{(n-1)}{n}\right)\left(\frac{p}{n}+q\right)-\left(p\frac{2}{n}+2q\right)$$

which gives

$$q'_s=\frac{\left(q\frac{m-1}{m}+r\frac{2}{n}\right)s'_2-\left(\frac{p}{n}+q\right)\left(3-\frac{1}{n}+\frac{q}{r}\frac{n}{2}\frac{m-1}{m}\right)}{\frac{pq}{r}\frac{1}{2n}\frac{m-1}{m}+q\frac{1}{n}\frac{m-1}{m}+p\frac{n-1}{n^3}}$$

If we plug in the formula for s'_2 from (30), we get the following expression for q'_s:

$$\begin{aligned} q'_s &= \frac{-\left(q\frac{m-1}{m}+r\frac{2}{n}\right)\sum_{i=2}^{m-2}\frac{\frac{p}{n}+q}{q\frac{i(m-i)}{m}+r\frac{2}{n}}-\left(\frac{p}{n}+q\right)\left(3-\frac{1}{n}+\frac{q}{r}\frac{n}{2}\frac{m-1}{m}\right)}{\frac{pq}{r}\frac{1}{2n}\frac{m-1}{m}+q\frac{1}{n}\frac{m-1}{m}+p\frac{n-1}{n^3}} \\ &= \frac{-\left(\frac{p}{n}+q\right)\left(\sum_{i=2}^{m-2}\frac{q\frac{m-1}{m}+r\frac{2}{n}}{q\frac{i(m-i)}{m}+r\frac{2}{n}}+3-\frac{1}{n}+\frac{q}{r}\frac{n}{2}\frac{m-1}{m}\right)}{\frac{pq}{r}\frac{1}{2n}\frac{m-1}{m}+q\frac{1}{n}\frac{m-1}{m}+p\frac{n-1}{n^3}} \end{aligned} \tag{34}$$

As in BD, Equation (8) moreover implies

$$q'_0=\frac{n-1}{n}q'_s \tag{35}$$

which links q'_s to q'_0, and Step 2 above gave us

$$q'_1=\left(\frac{p}{r}\frac{1}{2n}+1\right)q'_s+\frac{p+nq}{2r} \tag{36}$$

which links q'_s to q'_1. This implies that we have everything we need to complete Equations (18), (19) and (20) for the Shift process.

4.3 Three useful identities

The definition of relatedness (Equation 7) implies that relatednesses have to add up to 0.

$$\sum_{i=0}^{m-1}r_i=\sum_{i=0}^{m-1}\frac{q_i-\bar{q}}{1-\bar{q}}=\frac{m\bar{q}-m\bar{q}}{1-\bar{q}}=0$$

Equation (8) relates the relatedness within the group including self and excluding self in an obvious way, which we repeat here:

$$r_0 = \frac{1 + (n-1)r_s}{n}$$

Together, these imply that

$$\sum_{i=1}^{m-1} r_i = -r_0 = -\frac{1}{n} - \frac{(n-1)r_s}{n} \tag{37}$$

If we define r_o as the relatedness found through IBD probabilities, as in the previous subsection, to a randomly drawn individual from another group, where all other groups are equally likely to be drawn, then this is

$$r_o = \frac{1}{m-1} r_1 + \frac{1}{m-1} r_2 + \frac{1}{m-1} r_3 + \cdots + \frac{1}{m-1} r_{m-1} = \frac{1}{m-1} \sum_{i=1}^{m-1} r_i$$

or

$$\sum_{i=1}^{m-1} r_i = (m-1) r_o \tag{38}$$

Combining Equations (37) and (38), we get

$$r_o = -\frac{1}{n(m-1)} - \frac{(n-1)r_s}{n(m-1)} \tag{39}$$

None of the three identities depends on the update process, so they apply to BD as well as Shift.

4.4 Alternative derivation of the three identities

In the limit of $u \downarrow 0$, if two individuals are identical, they are identical by descent. Therefore, if we derive these identities more generally, using conditional probabilities, then they will coincide in this limit. Consider the dynamical system at hand, which is a Markov chain, in which every state is a vector k, where $k_i \in \{0, 1, ..., n\}$ is the number of cooperators in group i, for $i = 1, ..., m$. For every such population state, one can imagine hypothetical chance experiments, and define differences in conditional probabilities as we will below. These can then be aggregated, with weights attached to the population states. The weights could represent how often these states are visited relative to each other (this would be the rare-mutation dimorphic distribution from Allen and Tarnita, 2014, or the rare-mutation conditional distribution from Allen and McAvoy, 2018), but for now, all that matters is that p_k is the weight of population state k, and that $\sum_k p_k = 1$.

4.4.1 Within-group relatedness

Consider the following hypothetical chance experiment for a given population state k. Draw a random individual from the population, with every individual equally likely to be drawn. After this, go back to the same group, and randomly draw another individual from it. Then, for this state, one could define the proto-relatedness as

$$r_{s,k} = \mathbb{P}_{s,k}(C|C) - \mathbb{P}_{s,k}(C|D)$$

The subscript s for same group indicates that this measure is about the relatedness between two different individuals within the same group. The subscript k indicates which population state it pertains to.

Within group relatedness can now be defined as

$$r_s = \sum_k p_k \cdot r_{s,k} = \sum_k p_k \left(\mathbb{P}_{s,k}(C|C) - \mathbb{P}_{s,k}(C|D) \right)$$

4.4.2 Relatedness with an individual from a random other group

Now think of another chance experiment for a given population state k. Draw a random individual from the population, again with every individual equally likely to be drawn. After this, go to a different group, with all other groups equally likely to be chosen, and randomly draw another individual from that group. Then, for this state, proto-relatedness is

$$r_{o,k} = \mathbb{P}_{o,k}(C|C) - \mathbb{P}_{o,k}(C|D)$$

The subscript o for other group indicates that this measure is about the relatedness between two individuals in different groups.

Relatedness between two individuals from randomly chosen different groups can now be defined as

$$r_o = \sum_k p_k \cdot r_{o,k} = \sum_k p_k \left(\mathbb{P}_{o,k}(C|C) - \mathbb{P}_{o,k}(C|D) \right)$$

4.4.3 Relatedness between individuals that are i groups apart

With groups situated on the cycle, we can also define other chance experiments for a given population state k. First draw a random individual from the population, as before. After this, with probability $\frac{1}{2}$ go to the group that is i steps to the left of the first group, and with probability $\frac{1}{2}$ go to the group that is i steps to

the right of the first group, $i = 1, ..., m-1$. Randomly draw another individual from that group. Then, for this state, the i-step proto-relatedness, can now be defined as

$$r_{i,k} = \mathbb{P}_{i,k}(C|C) - \mathbb{P}_{i,k}(C|D)$$

for $i = 1, ..., m-1$, where $r_i = r_j$ if $i = m-j$ for all $1 \leq i \leq m-1$. The subscript i indicates that this measure is about the relatedness between two individuals in groups that are i steps away from each other.

Relatedness between two individuals that are i groups apart can now be defined as

$$r_i = \sum_k p_k \cdot r_{i,k} = \sum_k p_k \left(\mathbb{P}_{i,k}(C|C) - \mathbb{P}_{i,k}(C|D)\right)$$

4.4.4 Identity I

In order to relate these relatednesses to each other, assume that we consider a state k, for which $K = \sum_{j=0}^{m} k_j$ is the total number of cooperators in the population as a whole. Now imagine a chance experiment where we first draw a random individual from the population, with all individuals equally likely to be chosen, and then – without replacement – another individual from the population as a whole, with all remaining individuals equally likely to be drawn. Conditional on the first being a cooperator, the chance that the second is a cooperator can be written in two different ways, which must be equal to each other:

$$\frac{n-1}{nm-1}\mathbb{P}_{s,k}(C|C) + \frac{n(m-1)}{nm-1}\mathbb{P}_{o,k}(C|C) = \frac{K-1}{nm-1}$$

Conditional on the first being a defector, one can also express the chance that the second is a cooperator in two equivalent ways

$$\frac{n-1}{nm-1}\mathbb{P}_{s,k}(C|D) + \frac{n(m-1)}{nm-1}\mathbb{P}_{o,k}(C|D) = \frac{K}{nm-1}$$

These two identities together imply that

$$(n-1)\left(\mathbb{P}_{s,k}(C|C) - \mathbb{P}_{s,k}(C|D)\right) + n(m-1)(\mathbb{P}_{o,k}(C|C) - \mathbb{P}_{o,k}(C|D)) = -1$$

which implies that

$$r_{o,k} = -\frac{1}{n(m-1)} - \frac{n-1}{n(m-1)} r_{s,k}$$

Because the p_k add up to 1, this state-wise identity implies that if we aggregate over states accordingly, the following holds:

$$r_o = -\frac{1}{n(m-1)} - \frac{n-1}{n(m-1)} r_s$$

This is Equation (39).

4.4.5 Identity II

One can also go over the groups, according to their distance to the group from which the first individual was drawn. Then the equalities become:

$$\frac{n-1}{nm-1}\mathbb{P}_{s,k}(C|C) + \frac{n}{nm-1}\sum_{i=1}^{m-1}\mathbb{P}_{i,k}(C|C) = \frac{K-1}{nm-1}$$

and

$$\frac{n-1}{nm-1}\mathbb{P}_{s,k}(C|D) + \frac{n}{nm-1}\sum_{i=1}^{m-1}\mathbb{P}_{i,k}(C|D) = \frac{K}{nm-1}$$

These two identities together imply that

$$(n-1)(\mathbb{P}_{s,k}(C|C) - \mathbb{P}_{s,k}(C|D)) + n\left(\sum_{i=1}^{m-1}\mathbb{P}_{i,k}(C|C) - \sum_{i=1}^{m-1}\mathbb{P}_{i,k}(C|D)\right) = -1$$

which can be rewritten as

$$\sum_{i=1}^{m-1} r_{i,k} = -\frac{1}{n} - \frac{n-1}{n} r_{s,k}$$

Because the p_k add up to 1, this state-wise identity implies that if we aggregate over states accordingly, the following holds:

$$\sum_{i=1}^{m-1} r_i = -\frac{1}{n} - \frac{n-1}{n} r_s$$

This is Equation (37).

4.4.6 Identity III

Given Identity I, Identity II is also equivalent to

$$\sum_{i=1}^{m-1} r_i = (m-1) r_o$$

This is Equation (38).

5 Birth-Death versus Shift

In Section 3, we have found the critical b/c ratios for the the Birth-Death and the Shift process. For the BD process, the critical ratio was given in Condition (2). It is repeated below.

$$\frac{b}{c} > \frac{p}{q}\frac{m}{m-1}\frac{1-r_0}{n(r_0-r_1)}$$

For the Shift process, the critical ratio was given in Condition (4). This is also repeated below.

$$\frac{b}{c} > \frac{p}{q}\frac{1-r_0}{nr_0}$$

These thresholds are expressed as functions of p, q, r, m, and n, as well as relatednesses r_0 and r_1 – or, equivalently, r_s and r_1. These relatednesses will typically be different for different update processes.

In Section 4, we have computed r_0 and r_1, both for the Birth-Death process and for the Shift process, expressing them as functions of p, q, r, m and n as well. For both processes, Equations (18) and (19), reproduced below, apply.

$$\begin{aligned} r_0 &= \frac{(m-1)(q'_1-q'_0)+\sum_{i=1}^{m-1}s'_i}{q'_0+(m-1)q'_1+\sum_{i=1}^{m-1}s'_i} \\ r_1 &= \frac{-(q'_1-q'_0)+\sum_{i=1}^{m-1}s'_i}{q'_0+(m-1)q'_1+\sum_{i=1}^{m-1}s'_i} \end{aligned}$$

In these formulas, we still need to fill in q'_1, q'_0 and $\sum_{i=1}^{m-1}s'_i$, and these will differ between the two processes. For BD, we have Equations (21), (22), (23) and (24), reproduced below.

$$\begin{aligned} \sum_{i=1}^{m-1}s'_i &= -\frac{(m-1)(m-2)(m-3)}{6}\frac{\frac{p}{n}+q}{q\frac{m-1}{m}+r\frac{2}{n}} \\ q'_s &= \frac{-\left(\frac{p}{n}+q\right)\left(m-\frac{1}{n}+\frac{qn}{2r}\frac{m-1}{m}\right)}{\frac{pq}{r}\frac{1}{2n}\frac{m-1}{m}+q\frac{1}{n}\frac{m-1}{m}+p\frac{n-1}{n^3}} \\ q'_0 &= \frac{n-1}{n}q'_s \\ q'_1 &= \left(\frac{p}{r}\frac{1}{2n}+1\right)q'_s+\frac{p+nq}{2r} \end{aligned}$$

For Shift, we have Equations (33), (34), (35) and (36), reproduced below, where $\eta_i = \frac{\frac{p}{n}+q}{q\frac{i(m-i)}{m}+r\frac{2}{n}}$ is given by Equation (29).

$$\sum_{j=1}^{m-1} s'_j = \sum_{j=2}^{m-2} s'_j = -2(m-3)\eta_2 + \sum_{i=3}^{m-3}\left(\frac{m^2-m-2-2i(2m-3)+2i^2}{2}\right)\eta_i$$

$$q'_s = \frac{-\left(\frac{p}{n}+q\right)\left(\sum_{i=2}^{m-2}\frac{q\frac{m-1}{m}+r\frac{2}{n}}{q\frac{i(m-i)}{m}+r\frac{2}{n}}+3-\frac{1}{n}+\frac{q}{r}\frac{n}{2}\frac{m-1}{m}\right)}{\frac{pq}{r}\frac{1}{2n}\frac{m-1}{m}+q\frac{1}{n}\frac{m-1}{m}+p\frac{n-1}{n^3}}$$

$$q'_0 = \frac{n-1}{n}q'_s$$

$$q'_1 = \left(\frac{p}{r}\frac{1}{2n}+1\right)q'_s + \frac{p+nq}{2r}$$

For both processes, we have $q'_0 = \frac{n-1}{n}q'_s$ and $q'_1 = \left(\frac{p}{r}\frac{1}{2n}+1\right)q'_s + \frac{p+nq}{2r}$, even though the value of q'_s will differ between the two processes. Therefore, we can use these formulas to express difference $q'_1 - q'_0$ in the relatedness formulas in terms of model parameters and q'_s only.

$$q'_1 - q'_0 = \left(\frac{p}{r}\frac{1}{2n}+1\right)q'_s + \frac{p+nq}{2r} - \frac{n-1}{n}q'_s = \left(\frac{p}{r}\frac{1}{2n}+1-\frac{n-1}{n}\right)q'_s + \frac{p+nq}{2r}$$
$$= \left(\frac{p}{r}\frac{1}{2n}+\frac{1}{n}\right)q'_s + \frac{p+nq}{2r}$$

Now, if we plug this back into the relatedness formulas, we get the following expressions:

$$r_0 = \frac{(m-1)\left(\frac{p}{r}\frac{1}{2n}+\frac{1}{n}\right)q'_s + (m-1)\frac{p+nq}{2r} + \sum_{i=1}^{m-1}s'_i}{\left(\frac{n-1}{n}+(m-1)\left(\frac{p}{r}\frac{1}{2n}+1\right)\right)q'_s + (m-1)\frac{p+nq}{2r} + \sum_{i=1}^{m-1}s'_i}$$

$$r_1 = \frac{-\left(\frac{p}{r}\frac{1}{2n}+\frac{1}{n}\right)q'_s - \frac{p+nq}{2r} + \sum_{i=1}^{m-1}s'_i}{\left(\frac{n-1}{n}+(m-1)\left(\frac{p}{r}\frac{1}{2n}+1\right)\right)q'_s + (m-1)\frac{p+nq}{2r} + \sum_{i=1}^{m-1}s'_i}$$

Using the relatedness formulas and the relationships between IBD probabilities q'_s, q'_0 and q'_1, we can rewrite $1-r_0$ and r_0-r_1 as follows

$$1-r_0 = \frac{mq'_0}{q'_0+(m-1)q'_1+\sum_{i=1}^{m-1}s'_i} = \frac{m\frac{n-1}{n}q'_s}{\left((m-1)\left(\frac{p}{r}\frac{1}{2n}+1\right)+\frac{n-1}{n}\right)q'_s + (m-1)\frac{p+nq}{2r} + \sum_{i=1}^{m-1}s'_i}$$

$$r_0-r_1 = \frac{m(q'_1-q'_0)}{q'_0+(m-1)q'_1+\sum_{i=1}^{m-1}s'_i} = \frac{m\left(\frac{p}{r}\frac{1}{2n}+\frac{1}{n}\right)q'_s + m\frac{p+nq}{2r}}{\left((m-1)\left(\frac{p}{r}\frac{1}{2n}+1\right)+\frac{n-1}{n}\right)q'_s + (m-1)\frac{p+nq}{2r} + \sum_{i=1}^{m-1}s'_i}$$

If we plug these into the formulas for the critical ratios, we get, for BD,

$$\begin{aligned}\frac{b}{c} &> \frac{p}{q}\frac{1}{n}\frac{m}{m-1}\frac{\frac{mq'_0}{q'_0+(m-1)q'_1+\sum_{i=1}^{m-1}s'_i}}{\frac{m(q'_1-q'_0)}{q'_0+(m-1)q'_1+\sum_{i=1}^{m-1}s'_i}}\\ &= \frac{p}{q}\frac{1}{n}\frac{m}{m-1}\frac{q'_0}{q'_1-q'_0}\\ &= \frac{p}{q}\frac{1}{n}\frac{m}{m-1}\frac{\frac{n-1}{n}q'_s}{\left(\frac{p}{r}\frac{1}{2n}+\frac{1}{n}\right)q'_s+\frac{p+nq}{2r}}\\ &= \frac{p}{q}\frac{n-1}{n^2}\frac{m}{m-1}\frac{q'_s}{\left(\frac{p}{r}\frac{1}{2n}+\frac{1}{n}\right)q'_s+\frac{p+nq}{2r}}\end{aligned}\tag{40}$$

And for Shift, we get

$$\begin{aligned}\frac{b}{c} &> \frac{p}{q}\frac{1}{n}\frac{\frac{mq'_0}{q'_0+(m-1)q'_1+\sum_{i=1}^{m-1}s'_i}}{\frac{(m-1)(q'_1-q'_0)+\sum_{i=1}^{m-1}s'_i}{q'_0+(m-1)q'_1+\sum_{i=1}^{m-1}s'_i}}\\ &= \frac{p}{q}\frac{1}{n}\frac{mq'_0}{(m-1)(q'_1-q'_0)+\sum_{i=1}^{m-1}s'_i}\\ &= \frac{p}{q}\frac{1}{n}\frac{m\frac{n-1}{n}q'_s}{(m-1)\left(\frac{p}{r}\frac{1}{2n}+\frac{1}{n}\right)q'_s+(m-1)\frac{p+nq}{2r}+\sum_{i=1}^{m-1}s'_i}\\ &= \frac{p}{q}\frac{n-1}{n^2}\frac{mq'_s}{(m-1)\left(\frac{p}{r}\frac{1}{2n}+\frac{1}{n}\right)q'_s+(m-1)\frac{p+nq}{2r}+\sum_{i=1}^{m-1}s'_i}\\ &= \frac{p}{q}\frac{n-1}{n^2}\frac{m}{m-1}\frac{q'_s}{\left(\frac{p}{r}\frac{1}{2n}+\frac{1}{n}\right)q'_s+\frac{p+nq}{2r}+\frac{1}{m-1}\sum_{i=1}^{m-1}s'_i}\end{aligned}\tag{41}$$

What we will do in this section, is to compare these two critical b/c ratios, and show that the one for BD is always higher than the one for Shift.

We start by comparing the IBD probabilities q'_s, which now get a superscript, depending on the update process.

$$\begin{aligned}(q'_s)^{BD} &= -\frac{\left(\frac{p}{n}+q\right)\left(m-\frac{1}{n}+\frac{qn}{2r}\frac{m-1}{m}\right)}{\frac{pq}{r}\frac{1}{2n}\frac{m-1}{m}+q\frac{1}{n}\frac{m-1}{m}+p\frac{n-1}{n^3}}\\ (q'_s)^{Shift} &= -\frac{\left(\frac{p}{n}+q\right)\left[\sum_{i=2}^{m-2}\frac{q\frac{m-1}{m}+r\frac{2}{n}}{q\frac{i(m-i)}{m}+r\frac{2}{n}}+3-\frac{1}{n}+\frac{q}{r}\frac{n}{2}\frac{m-1}{m}\right]}{\frac{pq}{r}\frac{1}{2n}\frac{m-1}{m}+q\frac{1}{n}\frac{m-1}{m}+p\frac{n-1}{n^3}}\\ (q'_s)^{BD}-(q'_s)^{Shift} &= -\frac{\left(\frac{p}{n}+q\right)\left(m-\sum_{i=2}^{m-2}\frac{q\frac{m-1}{m}+r\frac{2}{n}}{q\frac{i(m-i)}{m}+r\frac{2}{n}}-3\right)}{\frac{pq}{r}\frac{1}{2n}\frac{m-1}{m}+q\frac{1}{n}\frac{m-1}{m}+p\frac{n-1}{n^3}}\end{aligned}$$

$$(q'_s)^{BD} = (q'_s)^{Shift} - \frac{\left(\frac{p}{n}+q\right)\left(m-3-\sum_{i=2}^{m-2}\frac{q\frac{m-1}{m}+r\frac{2}{n}}{q\frac{i(m-i)}{m}+r\frac{2}{n}}\right)}{\frac{pq}{r}\frac{1}{2n}\frac{m-1}{m}+q\frac{1}{n}\frac{m-1}{m}+p\frac{n-1}{n^3}} := (q'_s)^{Shift} - A \tag{42}$$

For the terms $\frac{q\frac{m-1}{m}+r\frac{2}{n}}{q\frac{i(m-i)}{m}+r\frac{2}{n}}$ within the sum above, we have the following calculations.

$$\frac{i(m-i)}{m}-\frac{m-1}{m}=\frac{i(m-i)-m+1}{m}=\frac{im-i^2-m+1}{m}=\frac{m(i-1)-(i^2-1)}{m}=\frac{m(i-1)-(i-1)(i+1)}{m}$$
$$=\frac{(i-1)(m-i-1)}{m}$$

For any $2 \leq i \leq m-2$, we have $i-1>0$ and $m-i-1>0$, which implies

$$\begin{aligned}
0 &< \frac{i(m-i)}{m}-\frac{m-1}{m}\\
\frac{m-1}{m} &< \frac{i(m-i)}{m}\\
q\frac{m-1}{m} &< q\frac{i(m-i)}{m}\\
q\frac{m-1}{m}+r\frac{2}{n} &< q\frac{i(m-i)}{m}+r\frac{2}{n}\\
\frac{q\frac{m-1}{m}+r\frac{2}{n}}{q\frac{i(m-i)}{m}+r\frac{2}{n}} &< 1
\end{aligned}$$

Therefore, every term within the summation in the numerator of Equation (42) is less than one. Hence, the sum is less than $m-3$, and the numerator is positive, which implies that A is positive. Therefore, $(q_s')^{BD}$ is less than $(q_s')^{Shift}$, and since we know that both q_s''es are negative, this implies that $(q_s')^{BD}$ is "more negative" than $(q_s')^{Shift}$.

To compare the critical ratios for the two processes – given below – we need to compare their last terms, as their first three terms are identical.

$$\frac{b}{c}>\frac{p}{q}\frac{n-1}{n^2}\frac{m}{m-1}\frac{(q_s')^{BD}}{\left(\frac{p}{r}\frac{1}{2n}+\frac{1}{n}\right)(q_s')^{BD}+\frac{p+nq}{2r}} \tag{43}$$

for BD, and

$$\frac{b}{c}>\frac{p}{q}\frac{n-1}{n^2}\frac{m}{m-1}\frac{(q_s')^{Shift}}{\left(\frac{p}{r}\frac{1}{2n}+\frac{1}{n}\right)(q_s')^{Shift}+\frac{p+nq}{2r}+\frac{1}{m-1}\sum_{i=1}^{m-1}(s_i')^{Shift}} \tag{44}$$

for Shift.

5.1 When m is odd

In the calculations in this and the next subsection, we assume that $m>3$ and $n>1$ since these are the interesting cases. If $m \leq 3$, the two update processes become identical, and so do their thresholds. And if

$n = 1$, in every individual event, the offspring replaces the parent and nothing changes in the population state.

First assume that m is odd – the case where m is even is treated below. If m is odd, we can rewrite $\sum_{i=2}^{m-2}(s_i')^{Shift}$ as follows:

$$\begin{aligned}
\sum_{i=2}^{m-2}(s_i')^{Shift} &= -2(m-3)\frac{\frac{p}{n}+q}{q\frac{2(m-2)}{m}+r\frac{2}{n}} + \sum_{i=3}^{m-3}\left(\frac{m^2-m-2-2i(2m-3)+2i^2}{2}\right)\frac{\frac{p}{n}+q}{q\frac{i(m-i)}{m}+r\frac{2}{n}} \\
&= -2(m-3)\frac{\frac{p}{n}+q}{q\frac{2(m-2)}{m}+r\frac{2}{n}} + \sum_{i=3}^{(m-1)/2} 2m\left(\frac{m-1}{m}-\frac{i(m-i)}{m}\right)\frac{\frac{p}{n}+q}{q\frac{i(m-i)}{m}+r\frac{2}{n}} \\
&= -2(m-3)\frac{\frac{p}{n}+q}{q\frac{2(m-2)}{m}+r\frac{2}{n}} - 2m\sum_{i=3}^{(m-1)/2}\left(\frac{i(m-i)}{m}-\frac{m-1}{m}\right)\frac{\frac{p}{n}+q}{q\frac{i(m-i)}{m}+r\frac{2}{n}}
\end{aligned} \tag{45}$$

Going from the first to the second line, we gather the coefficients of $\frac{\frac{p}{n}+q}{q\frac{i(m-i)}{m}+r\frac{2}{n}}$ for i and $m-i$ within the sum since that is when these terms are the same. These coefficients then add up to $\frac{m^2-m-2-2i(2m-3)+2i^2}{2} + \frac{m^2-m-2-2(m-i)(2m-3)+2(m-i)^2}{2} = 2(m-1)-2i(m-i)$.

We need to find out whether the critical ratio for the BD process is always larger than that of the Shift process; hence, we need to compare the right-hand sides of the above two inequalities that give the critical b/c ratios, given in Conditions (43) and (44). To do so, we start with comparing a few terms to zero; and step-by-step, we will make our way to the equations above. We start by showing that the four terms below are positive:

- Term 1:

$$\frac{2m}{m-1} - \frac{2m}{2m\frac{r}{qn}\left(m-\frac{1}{n}\right)+m-1} > 0$$

  since $2m\frac{r}{qn}\left(m-\frac{1}{n}\right) > 0$. Hence,

$$\frac{2m}{m-1} - \frac{\frac{qn}{r}}{m-\frac{1}{n}+\frac{qn}{2r}\frac{m-1}{m}} > 0$$

- Term 2: For $1 < i < m-1$,

$$\frac{1}{q\frac{i(m-i)}{m}+r\frac{2}{n}} > 0$$

- Term 3: As seen before,

$$\frac{i(m-i)}{m} - \frac{m-1}{m} = \frac{(i-1)(m-i-1)}{m}$$

For i ranging from 2 to $m-2$, we have $i-1>0$ and $m-i-1>0$. Therefore,

$$\frac{i(m-i)}{m} - \frac{m-1}{m} > 0$$

- Term 4:

$$\frac{2(m-3)}{m-1} - \frac{2(m-3)}{2m\frac{r}{qn}\left(m-\frac{1}{n}\right)+m-1} > 0$$

since $2m\frac{r}{qn}\left(m-\frac{1}{n}\right) > 0$, and also

$$\frac{1}{q\frac{2(m-2)}{m}+r\frac{2}{n}} > 0$$

Hence,

$$\left(\frac{2(m-3)}{m-1} - \frac{\frac{qn}{r}\frac{m-3}{m}}{m-\frac{1}{n}+\frac{qn}{2r}\frac{m-1}{m}}\right)\frac{1}{q\frac{2(m-2)}{m}+r\frac{2}{n}} > 0$$

Since each of the terms above are individually positive for $1<i<m-1$, their products and sums will be positive as well. If we multiply the first three terms for a given i, the resulting product will be positive. If we sum these products over i, where $3 \leq i \leq \frac{m-1}{2}$, the resulting sum will be positive as well, since each term in the summation is positive. And finally, we add the fourth term above to the summation to reach the expression below, which is again positive:

$$\begin{aligned} 0 < &\left(\frac{2(m-3)}{m-1} - \frac{\frac{qn}{r}\frac{m-3}{m}}{m-\frac{1}{n}+\frac{qn}{2r}\frac{m-1}{m}}\right)\frac{1}{q\frac{2(m-2)}{m}+r\frac{2}{n}} \\ &+ \sum_{i=3}^{(m-1)/2}\left(\frac{2m}{m-1} - \frac{\frac{qn}{r}}{m-\frac{1}{n}+\frac{qn}{2r}\frac{m-1}{m}}\right)\left(\frac{i(m-i)}{m} - \frac{m-1}{m}\right)\frac{1}{q\frac{i(m-i)}{m}+r\frac{2}{n}} \end{aligned}$$

Now, we split this expression into two parts, depending on the sign of the terms, and put the terms with a negative coefficient on the left hand side:

$$
\begin{aligned}
0 < &\frac{2(m-3)}{m-1}\frac{1}{q^{\frac{2(m-2)}{m}}+r^{\frac{2}{n}}} - \frac{\frac{n}{r}}{m-\frac{1}{n}+\frac{qn}{2r}\frac{m-1}{m}}\frac{q^{\frac{m-3}{m}}}{q^{\frac{2(m-2)}{m}}+r^{\frac{2}{n}}} \\
&+\sum_{i=3}^{(m-1)/2}\left(\frac{2m}{m-1}\left(\frac{i(m-i)}{m}-\frac{m-1}{m}\right)-\frac{\frac{n}{r}}{m-\frac{1}{n}+\frac{qn}{2r}\frac{m-1}{m}}\left(q\frac{i(m-i)}{m}-q\frac{m-1}{m}\right)\right)\frac{1}{q^{\frac{i(m-i)}{m}}+r^{\frac{2}{n}}} \\
0 < &\frac{2(m-3)}{m-1}\frac{1}{q^{\frac{2(m-2)}{m}}+r^{\frac{2}{n}}} - \frac{\frac{n}{r}}{m-\frac{1}{n}+\frac{qn}{2r}\frac{m-1}{m}}\frac{q^{\frac{2(m-2)}{m}}-q^{\frac{m-1}{m}}}{q^{\frac{2(m-2)}{m}}+r^{\frac{2}{n}}} \\
&+\sum_{i=3}^{(m-1)/2}\left(\frac{2m}{m-1}\left(\frac{i(m-i)}{m}-\frac{m-1}{m}\right)-\frac{\frac{n}{r}}{m-\frac{1}{n}+\frac{qn}{2r}\frac{m-1}{m}}\left(q\frac{i(m-i)}{m}-q\frac{m-1}{m}\right)\right)\frac{1}{q^{\frac{i(m-i)}{m}}+r^{\frac{2}{n}}} \\
0 < &\frac{2(m-3)}{m-1}\frac{1}{q^{\frac{2(m-2)}{m}}+r^{\frac{2}{n}}} - \frac{\frac{n}{r}}{m-\frac{1}{n}+\frac{qn}{2r}\frac{m-1}{m}}\frac{q^{\frac{2(m-2)}{m}}-q^{\frac{m-1}{m}}}{q^{\frac{2(m-2)}{m}}+r^{\frac{2}{n}}} \\
&+\sum_{i=3}^{(m-1)/2}\frac{2m}{m-1}\left(\frac{i(m-i)}{m}-\frac{m-1}{m}\right)\frac{1}{q^{\frac{i(m-i)}{m}}+r^{\frac{2}{n}}} \\
&-\sum_{i=3}^{(m-1)/2}\frac{\frac{n}{r}}{m-\frac{1}{n}+\frac{qn}{2r}\frac{m-1}{m}}\left(q\frac{i(m-i)}{m}-q\frac{m-1}{m}\right)\frac{1}{q^{\frac{i(m-i)}{m}}+r^{\frac{2}{n}}}
\end{aligned}
$$

This leads to

$$
\begin{aligned}
&\frac{\frac{n}{r}}{m-\frac{1}{n}+\frac{qn}{2r}\frac{m-1}{m}}\left(\sum_{i=2}^{(m-1)/2}\frac{q^{\frac{i(m-i)}{m}}-q^{\frac{m-1}{m}}}{q^{\frac{i(m-i)}{m}}+r^{\frac{2}{n}}}\right) \\
&\qquad < \frac{1}{m-1}\left(2(m-3)\frac{1}{q^{\frac{2(m-2)}{m}}+r^{\frac{2}{n}}}+2m\sum_{i=3}^{(m-1)/2}\left(\frac{i(m-i)}{m}-\frac{m-1}{m}\right)\frac{1}{q^{\frac{i(m-i)}{m}}+r^{\frac{2}{n}}}\right)
\end{aligned}
$$

Notice that the whole term inside the parentheses on the right hand side is the summation from Equation (45) divided by $-\left(\frac{p}{n}+q\right)$, so we can rewrite it as $-\frac{1}{\frac{p}{n}+q}\sum_{i=2}^{m-2}(s'_i)^{Shift}$:

$$\frac{\frac{n}{r}}{m-\frac{1}{n}+\frac{qn}{2r}\frac{m-1}{m}}\left(\sum_{i=2}^{(m-1)/2}\frac{q\frac{i(m-i)}{m}-q\frac{m-1}{m}}{q\frac{i(m-i)}{m}+r\frac{2}{n}}\right) < \frac{1}{m-1}\frac{1}{\frac{p}{n}+q}\left(-\sum_{i=2}^{m-2}(s_i')^{Shift}\right)$$

$$\frac{\frac{n}{r}}{m-\frac{1}{n}+\frac{qn}{2r}\frac{m-1}{m}}\left(\sum_{i=2}^{(m-1)/2}\frac{q\frac{i(m-i)}{m}+r\frac{2}{n}-\left(q\frac{m-1}{m}+r\frac{2}{n}\right)}{q\frac{i(m-i)}{m}+r\frac{2}{n}}\right) < \frac{1}{m-1}\frac{1}{\frac{p}{n}+q}\left(-\sum_{i=2}^{m-2}(s_i')^{Shift}\right)$$

$$\frac{2\frac{n}{2r}}{m-\frac{1}{n}+\frac{qn}{2r}\frac{m-1}{m}}\left(\sum_{i=2}^{(m-1)/2}\left(1-\frac{q\frac{m-1}{m}+r\frac{2}{n}}{q\frac{i(m-i)}{m}+r\frac{2}{n}}\right)\right) < \frac{1}{m-1}\frac{1}{\frac{p}{n}+q}\left(-\sum_{i=2}^{m-2}(s_i')^{Shift}\right)$$

$$\frac{\frac{n}{2r}}{m-\frac{1}{n}+\frac{qn}{2r}\frac{m-1}{m}}\left(\sum_{i=2}^{m-2}\left(1-\frac{q\frac{m-1}{m}+r\frac{2}{n}}{q\frac{i(m-i)}{m}+r\frac{2}{n}}\right)\right) < \frac{1}{m-1}\frac{1}{\frac{p}{n}+q}\left(-\sum_{i=2}^{m-2}(s_i')^{Shift}\right)$$

$$\frac{\frac{n}{2r}}{m-\frac{1}{n}+\frac{qn}{2r}\frac{m-1}{m}}\left(m-3-\sum_{i=2}^{m-2}\frac{q\frac{m-1}{m}+r\frac{2}{n}}{q\frac{i(m-i)}{m}+r\frac{2}{n}}\right) < \frac{1}{m-1}\frac{1}{\frac{p}{n}+q}\left(-\sum_{i=2}^{m-2}(s_i')^{Shift}\right)$$

Going from the third to the fourth line above, we use that the terms for i and $m-i$ in the latter summation are always the same. Also we rewrite $\sum_{i=2}^{m-2}1$ as $m-3$ in the last line. Now, multiply every term with $\frac{p}{n}+q$ to get;

$$\frac{\frac{p+nq}{2r}}{m-\frac{1}{n}+\frac{qn}{2r}\frac{m-1}{m}}\left(m-3-\sum_{i=2}^{m-2}\frac{q\frac{m-1}{m}+r\frac{2}{n}}{q\frac{i(m-i)}{m}+r\frac{2}{n}}\right) < \frac{1}{m-1}\left(-\sum_{i=2}^{m-2}(s_i')^{Shift}\right)$$

Now, if we multiply both sides with -1, the sign also changes:

$$-\frac{\frac{p+nq}{2r}}{m-\frac{1}{n}+\frac{qn}{2r}\frac{m-1}{m}}\left(m-3-\sum_{i=2}^{m-2}\frac{q\frac{m-1}{m}+r\frac{2}{n}}{q\frac{i(m-i)}{m}+r\frac{2}{n}}\right) > \frac{1}{m-1}\left(\sum_{i=2}^{m-2}(s_i')^{Shift}\right)$$

Another step of re-arranging the terms above gives us

$$-\frac{p+nq}{2r}\left(\frac{m-3-\sum_{i=2}^{m-2}\frac{q\frac{m-1}{m}+r\frac{2}{n}}{q\frac{i(m-i)}{m}+r\frac{2}{n}}}{m-\frac{1}{n}+\frac{qn}{2r}\frac{m-1}{m}}\right) > \frac{1}{m-1}\left(\sum_{i=2}^{m-2}(s_i')^{Shift}\right)$$

Multiplying and dividing the left hand side by the term $\left(\frac{p}{n}+q\right)/\left(\frac{pq}{r}\frac{1}{2n}\frac{m-1}{m}+q\frac{1}{n}\frac{m-1}{m}+p\frac{n-1}{n^3}\right)$:

$$-\frac{p+nq}{2r}\left(\frac{\frac{\left(\frac{p}{n}+q\right)\left(m-3-\sum_{i=2}^{m-2}\frac{q\frac{m-1}{m}+r\frac{2}{n}}{q\frac{i(m-i)}{m}+r\frac{2}{n}}\right)}{\frac{pq}{r}\frac{1}{2n}\frac{m-1}{m}+q\frac{1}{n}\frac{m-1}{m}+p\frac{n-1}{n^3}}}{\frac{\left(\frac{p}{n}+q\right)\left(m-\frac{1}{n}+\frac{qn}{2r}\frac{m-1}{m}\right)}{\frac{pq}{r}\frac{1}{2n}\frac{m-1}{m}+q\frac{1}{n}\frac{m-1}{m}+p\frac{n-1}{n^3}}}\right) > \frac{1}{m-1}\sum_{i=1}^{m-1}(s_i')^{Shift} \tag{46}$$

Notice that the term in the numerator within the parentheses on the left hand side is equal to $A = (q_s')^{Shift} - (q_s')^{BD}$ from Equation (42), and that the denominator is equal to $-(q_s')^{BD}$ from Equation (22).

$$-\frac{p+nq}{2r}\left(\frac{(q_s')^{Shift}-(q_s')^{BD}}{-(q_s')^{BD}}\right) > \frac{1}{m-1}\sum_{i=1}^{m-1}(s_i')^{Shift}$$

$$\frac{p+nq}{2r}\left(\frac{(q_s')^{Shift}-(q_s')^{BD}}{(q_s')^{BD}}\right) > \frac{1}{m-1}\sum_{i=1}^{m-1}(s_i')^{Shift}$$

If we divide both sides by $(q_s')^{Shift}$,

$$\frac{p+nq}{2r}\left(\frac{(q_s')^{Shift}-(q_s')^{BD}}{(q_s')^{Shift}(q_s')^{BD}}\right) < \frac{1}{m-1}\frac{1}{(q_s')^{Shift}}\sum_{i=1}^{m-1}(s_i')^{Shift}$$

where the sign of the inequality changes since we multiply both sides with a negative term. Using $\frac{(q_s')^{Shift}-(q_s')^{BD}}{(q_s')^{Shift}(q_s')^{BD}} = \frac{1}{(q_s')^{BD}} - \frac{1}{(q_s')^{Shift}}$, we can rewrite the above inequality as follows,

$$\frac{p+nq}{2r}\left(\frac{1}{(q_s')^{BD}}-\frac{1}{(q_s')^{Shift}}\right) < \frac{1}{m-1}\frac{1}{(q_s')^{Shift}}\sum_{i=1}^{m-1}(s_i')^{Shift}$$

$$\frac{p+nq}{2r}\frac{1}{(q_s')^{BD}} < \frac{p+nq}{2r}\frac{1}{(q_s')^{Shift}}+\frac{1}{m-1}\frac{1}{(q_s')^{Shift}}\sum_{i=1}^{m-1}(s_i')^{Shift}$$

Add $\frac{p}{r}\frac{1}{2n}+\frac{1}{n}$ to both sides

$$\frac{p}{r}\frac{1}{2n}+\frac{1}{n}+\frac{p+nq}{2r}\frac{1}{(q_s')^{BD}} < \frac{p}{r}\frac{1}{2n}+\frac{1}{n}+\frac{p+nq}{2r}\frac{1}{(q_s')^{Shift}}+\frac{1}{m-1}\sum_{i=1}^{m-1}(s_i')^{Shift}\frac{1}{(q_s')^{Shift}}$$

Reverse the numerator and the denominator on both sides of the inequality,

$$\frac{1}{\frac{p}{r}\frac{1}{2n}+\frac{1}{n}+\frac{p+nq}{2r}\frac{1}{(q_s')^{BD}}} > \frac{1}{\frac{p}{r}\frac{1}{2n}+\frac{1}{n}+\frac{p+nq}{2r}\frac{1}{(q_s')^{Shift}}+\frac{1}{m-1}\sum_{i=1}^{m-1}(s_i')^{Shift}\frac{1}{(q_s')^{Shift}}}$$

$$\frac{(q_s')^{BD}}{\left(\frac{p}{r}\frac{1}{2n}+\frac{1}{n}\right)(q_s')^{BD}+\frac{p+nq}{2r}} > \frac{(q_s')^{Shift}}{\left(\frac{p}{r}\frac{1}{2n}+\frac{1}{n}\right)(q_s')^{Shift}+\frac{p+nq}{2r}+\frac{1}{m-1}\sum_{i=1}^{m-1}(s_i')^{Shift}}$$

where the sign of the inequality changes again on the first line above since we are reversing the fractions that we are comparing. Now, if we multiply both sides by $\frac{p}{q}\frac{n-1}{n^2}\frac{m}{m-1}$, we arrive at the inequality:

$$\frac{p}{q}\frac{n-1}{n^2}\frac{m}{m-1}\frac{(q_s')^{BD}}{\left(\frac{p}{r}\frac{1}{2n}+\frac{1}{n}\right)(q_s')^{BD}+\frac{p+nq}{2r}} > \frac{p}{q}\frac{n-1}{n^2}\frac{m}{m-1}\frac{(q_s')^{Shift}}{\left(\frac{p}{r}\frac{1}{2n}+\frac{1}{n}\right)(q_s')^{Shift}+\frac{p+nq}{2r}+\frac{1}{m-1}\sum_{i=1}^{m-1}(s_i')^{Shift}}$$

In this last inequality, the left hand side is the critical b/c ratio for the BD process, and the right hand side is the critical b/c ratio for the Shift process, given in Conditions (43) and (44), respectively.

5.2 When m is even

If m is even, we can rewrite $\sum_{i=2}^{m-2}(s_i')^{Shift}$ as follows:

$$\begin{aligned}\sum_{i=2}^{m-2}(s_i')^{Shift} &= -2(m-3)\frac{\frac{p}{n}+q}{q\frac{2(m-2)}{m}+r\frac{2}{n}}+\sum_{i=3}^{m-3}\left(\frac{m^2-m-2-2i(2m-3)+2i^2}{2}\right)\frac{\frac{p}{n}+q}{q\frac{i(m-i)}{m}+r\frac{2}{n}}\\ &= -2(m-3)\frac{\frac{p}{n}+q}{q\frac{2(m-2)}{m}+r\frac{2}{n}}-2m\sum_{i=3}^{m/2-1}\left(\frac{i(m-i)}{m}-\frac{m-1}{m}\right)\frac{\frac{p}{n}+q}{q\frac{i(m-i)}{m}+r\frac{2}{n}}-\frac{(m-2)^2}{4}\frac{\frac{p}{n}+q}{q\frac{\frac{m}{2}\frac{m}{2}}{m}+r\frac{2}{n}}\end{aligned} \tag{47}$$

This we can do for reasons similar to the ones when m is odd.

To compare the critical ratios for the two update processes from Equations (43) and (44), we are going to follow a very similar path to the case above in Section 5.1. We start with adding a fifth term to the terms given in the previous subsection; and step-by-step, we are going to reach the equations for the critical ratios of the two update processes. We have shown previously that the Terms 1 through 4 are positive. Here, we add another term and show that it is also positive:

- Term 5:

$$\frac{(m-2)^2}{4(m-1)}-\frac{(m-2)^2}{8m\frac{r}{qn}\left(m-\frac{1}{n}\right)+4(m-1)} > 0$$

  since $8m\frac{r}{qn}\left(m-\frac{1}{n}\right) > 0$, therefore,

$$\frac{(m-2)^2}{4(m-1)} - \frac{\frac{qn}{r}\frac{(m-2)^2}{8m}}{m - \frac{1}{n} + \frac{qn}{2r}\frac{m-1}{m}} > 0$$

and

$$\frac{1}{q\frac{m^2}{4m} + r\frac{2}{n}} > 0$$

Hence,

$$\left(\frac{(m-2)^2}{4(m-1)} - \frac{\frac{qn}{r}\frac{(m-2)^2}{8m}}{m - \frac{1}{n} + \frac{qn}{2r}\frac{m-1}{m}}\right)\frac{1}{q\frac{m^2}{4m} + r\frac{2}{n}} > 0$$

Now, if we multiply Term 1, 2 and 3 and sum these products over $3 \leq i \leq \frac{m-2}{2} = \frac{m}{2} - 1$, add Term 4 to the summation from the previous subsection, we know that the resulting term will be positive. Now, we add Term 5 above, which is also positive, to get

$$\begin{aligned}
0 < &\left(\frac{2(m-3)}{m-1} - \frac{\frac{qn}{r}\frac{m-3}{m}}{m - \frac{1}{n} + \frac{qn}{2r}\frac{m-1}{m}}\right)\frac{1}{q\frac{2(m-2)}{m} + r\frac{2}{n}} \\
&+ \sum_{i=3}^{m/2-1}\left(\frac{2m}{m-1} - \frac{\frac{qn}{r}}{m - \frac{1}{n} + \frac{qn}{2r}\frac{m-1}{m}}\right)\left(\frac{i(m-i)}{m} - \frac{m-1}{m}\right)\frac{1}{q\frac{i(m-i)}{m} + r\frac{2}{n}} \\
&+ \left(\frac{(m-2)^2}{4(m-1)} - \frac{\frac{qn}{r}\frac{(m-2)^2}{8m}}{m - \frac{1}{n} + \frac{qn}{2r}\frac{m-1}{m}}\right)\frac{1}{q\frac{m^2}{4m} + r\frac{2}{n}}
\end{aligned}$$

We are re-arranging the expression above such that every term with a negative (positive) sign is on the left hand side (right hand side),

$$\begin{aligned}
&\frac{\frac{qn}{r}\left(\frac{2(m-2)}{m} - \frac{m-1}{m}\right)}{m - \frac{1}{n} + \frac{qn}{2r}\frac{m-1}{m}}\frac{1}{q\frac{2(m-2)}{m} + r\frac{2}{n}} + \sum_{i=3}^{m/2-1}\frac{\frac{qn}{r}}{m - \frac{1}{n} + \frac{qn}{2r}\frac{m-1}{m}}\left(\frac{i(m-i)}{m} - \frac{m-1}{m}\right)\frac{1}{q\frac{i(m-i)}{m} + r\frac{2}{n}} \\
&\quad + \frac{\frac{qn}{r}\frac{(m-2)^2}{8m}}{m - \frac{1}{n} + \frac{qn}{2r}\frac{m-1}{m}}\frac{1}{q\frac{m^2}{4m} + r\frac{2}{n}} \\
&\quad < \frac{2(m-3)}{m-1}\frac{1}{q\frac{2(m-2)}{m} + r\frac{2}{n}} + \sum_{i=3}^{m/2-1}\frac{2m}{m-1}\left(\frac{i(m-i)}{m} - \frac{m-1}{m}\right)\frac{1}{q\frac{i(m-i)}{m} + r\frac{2}{n}} \\
&\quad + \frac{(m-2)^2}{4(m-1)}\frac{1}{q\frac{m^2}{4m} + r\frac{2}{n}}
\end{aligned}$$

Here, we used $\frac{2(m-2)}{m} - \frac{m-1}{m} = \frac{m-3}{m}$ on the first line. Realize that the right hand side in the last line above is equal to $-\frac{1}{m-1}\frac{1}{\frac{p}{n}+q}\sum_{i=2}^{m-2}(s_i')^{Shift}$ where $\sum_{i=2}^{m-2}(s_i')^{Shift}$ is defined in Equation (47).

Using similar steps as in the previous subsection and the fact that

$$\frac{\frac{m}{2}\left(m-\frac{m}{2}\right)}{m} - \frac{m-1}{m} = \frac{m^2}{4m} - \frac{m-1}{m} = \frac{(m-2)^2}{4m}$$

and

$$\frac{q\frac{m^2}{4m} - q\frac{m-1}{m}}{q\frac{m^2}{4m} + r\frac{2}{n}} = 1 - \frac{q\frac{m-1}{m} + r\frac{2}{n}}{q\frac{m^2}{4m} + r\frac{2}{n}}$$

we get

$$\frac{\frac{n}{2r}}{m - \frac{1}{n} + \frac{qn}{2r}\frac{m-1}{m}}\left(m-3-\sum_{i=2}^{m-2}\frac{q\frac{m-1}{m}+\frac{2r}{n}}{q\frac{i(m-i)}{m}+r\frac{2}{n}}\right) < -\frac{1}{m-1}\frac{1}{\frac{p}{n}+q}\sum_{i=2}^{m-2}(s_i')^{Shift}$$

Multiplying both sides by $-\left(\frac{p}{n}+q\right)$, which changes the sign of the inequality, and multiplying and dividing the left hand side by $\left(\frac{p}{n}+q\right) / \left(\frac{pq}{r}\frac{1}{2n}\frac{m-1}{m} + q\frac{1}{n}\frac{m-1}{m} + p\frac{n-1}{n^3}\right)$ gives

$$-\frac{p+nq}{2r}\frac{1}{m-\frac{1}{n}+\frac{qn}{2r}\frac{m-1}{m}}\left(m-3-\sum_{i=2}^{m-2}\frac{q\frac{m-1}{m}+\frac{2r}{n}}{q\frac{i(m-i)}{m}+r\frac{2}{n}}\right) > \frac{1}{m-1}\sum_{i=2}^{m-2}(s_i')^{Shift}$$

$$\frac{p+nq}{2r}\left(\frac{\frac{\left(\frac{p}{n}+q\right)\left(m-3-\sum_{i=2}^{m-2}\frac{q\frac{m-1}{m}+r\frac{2}{n}}{q\frac{i(m-i)}{m}+r\frac{2}{n}}\right)}{\frac{pq}{r}\frac{1}{2n}\frac{m-1}{m}+q\frac{1}{n}\frac{m-1}{m}+p\frac{n-1}{n^3}}}{-\frac{\left(\frac{p}{n}+q\right)\left(m-\frac{1}{n}+\frac{qn}{2r}\frac{m-1}{m}\right)}{\frac{pq}{r}\frac{1}{2n}\frac{m-1}{m}+q\frac{1}{n}\frac{m-1}{m}+p\frac{n-1}{n^3}}}\right) > \frac{1}{m-1}\sum_{i=1}^{m-1}(s_i')^{Shift}$$

which is the same inequality as Inequality (46). Following the same steps after Inequality (46) in the previous subsection, we arrive at the result that

$$\frac{p}{q}\frac{n-1}{n^2}\frac{m}{m-1}\frac{(q_s')^{BD}}{\left(\frac{p}{r}\frac{1}{2n}+\frac{1}{n}\right)(q_s')^{BD}+\frac{p+nq}{2r}} > \frac{p}{q}\frac{n-1}{n^2}\frac{m}{m-1}\frac{(q_s')^{Shift}}{\left(\frac{p}{r}\frac{1}{2n}+\frac{1}{n}\right)(q_s')^{Shift}+\frac{p+nq}{2r}+\frac{1}{m-1}\sum_{i=1}^{m-1}(s_i')^{Shift}}$$

In this last inequality, the left hand side is the critical b/c ratio for the BD process, and the right hand side is the critical b/c ratio for the Shift process, given in Conditions (43) and (44), respectively.

5.3 Limit results for the number of groups approaching infinity

In this section, we explore the results in the limit where the number of groups m approaches infinity.

5.3.1 BD

For the BD process, we first repeat and rewrite Condition (40)

$$\begin{aligned}\frac{b}{c} &> \frac{p}{q}\frac{1}{n}\frac{m}{m-1}\frac{\frac{n-1}{n}q'_s}{\left(\frac{p}{r}\frac{1}{2n}+\frac{1}{n}\right)q'_s+\frac{p+nq}{2r}} \\ &= \frac{p}{q}\frac{1}{n}\frac{m}{m-1}\frac{\frac{n-1}{n}}{\frac{p}{r}\frac{1}{2n}+\frac{1}{n}+\frac{p+nq}{2r}\frac{1}{q'_s}}\end{aligned}$$

as well as Equation (22)

$$q'_s = \frac{-\left(\frac{p}{n}+q\right)\left(m-\frac{1}{n}+\frac{qn}{2r}\frac{m-1}{m}\right)}{\frac{pq}{r}\frac{1}{2n}\frac{m-1}{m}+q\frac{1}{n}\frac{m-1}{m}+p\frac{n-1}{n^3}}$$

Since $\lim_{m\to\infty} q'_s = \infty$, the critical b/c ratio for the BD process converges to

$$\begin{aligned}\frac{b}{c} &> \frac{p}{q}\frac{1}{n}\frac{\frac{n-1}{n}}{\frac{p}{r}\frac{1}{2n}+\frac{1}{n}} \\ &= \frac{p}{q}\frac{n-1}{n}\frac{1}{\frac{p}{2r}+1}\end{aligned} \tag{48}$$

The limit result of the critical ratio we found in Condition (48) is in line with numerical solutions for large m.

5.3.2 Shift

For the Shift process, we first repeat and rewrite Condition (41)

$$\begin{aligned}\frac{b}{c} &> \frac{p}{q}\frac{n-1}{n^2}\frac{m}{m-1}\frac{q'_s}{\left(\frac{p}{r}\frac{1}{2n}+\frac{1}{n}\right)q'_s+\frac{p+nq}{2r}+\frac{1}{m-1}\sum_{i=1}^{m-1}s'_i} \\ &= \frac{p}{q}\frac{n-1}{n^2}\frac{m}{m-1}\frac{1}{\frac{p}{r}\frac{1}{2n}+\frac{1}{n}+\frac{p+nq}{2r}\frac{1}{q'_s}+\frac{1}{m-1}\frac{1}{q'_s}\sum_{i=1}^{m-1}s'_i}\end{aligned}$$

We also have, from Equations (34) and (33), reproduced below, where $\eta_i = \frac{\frac{p}{n}+q}{q\frac{i(m-i)}{m}+r\frac{2}{n}}$ is given by Equation (29).

$$q_s' = \frac{-\left(\frac{p}{n}+q\right)\left(\sum_{i=2}^{m-2}\frac{q\frac{m-1}{m}+r\frac{2}{n}}{q\frac{i(m-i)}{m}+r\frac{2}{n}}+3-\frac{1}{n}+\frac{q}{r}\frac{n}{2}\frac{m-1}{m}\right)}{\frac{pq}{r}\frac{1}{2n}\frac{m-1}{m}+q\frac{1}{n}\frac{m-1}{m}+p\frac{n-1}{n^3}}$$

$$\sum_{j=1}^{m-1} s_j' = -2(m-3)\eta_2 + \sum_{i=3}^{m-3}\left(\frac{m^2-m-2-2i(2m-3)+2i^2}{2}\right)\eta_i$$

Now consider the sum in the numerator in q_s'. Since each term in this summation is less than 1, we have

$$\sum_{i=2}^{m-2}\frac{m-1+m\frac{r}{q}\frac{2}{n}}{i(m-i)+m\frac{r}{q}\frac{2}{n}} < m-3$$

and hence q_s' decreases at most proportional to m. On the other hand, $\sum_{j=1}^{m-1} s_j$ decreases proportional to m^2. Therefore, the term with $\sum_{j=1}^{m-1} s_j$ in the denominator of the critical b/c ratio for the Shift process dominates in the limit $m \to \infty$, and hence, the critical b/c ratio for Shift converges to

$$\frac{b}{c} > 0 \tag{49}$$

The limit result of the critical ratio we found in Condition (49) is in line with numerical solutions for large m.

5.3.3 Birth-Death versus Shift in the limit where number of groups approach infinity

In the previous subsection, we solved for the critical b/c ratios in the limit where the number of groups m approaches infinity. The limit results are repeated below for convenience.

$$\frac{b}{c} > \frac{p}{q}\frac{n-1}{n}\frac{1}{\frac{p}{2r}+1}$$

for BD, and

$$\frac{b}{c} > 0$$

for Shift. From these formulas, it is immediately clear that the critical b/c ratio for Shift is lower than the one for BD in the limit $m \to \infty$.

6 Simulations

Since our model quickly becomes intractable once we move away from the limit of weak selection, we also ran numerical simulations with different intensities of selection. In this section, we describe the details of those simulations.

We programmed the simulation version of model, presented in Section 2, in Matlab, where the population state is represented by a vector at each time step. In the remainder of the text, we will call this "the population vector". Each entry in the population vector represents the number of cooperators in a group at a given location on the circle. The group size is fixed in our model, and therefore the number of defectors is implied by the number of cooperators. This vector represents a circle, and therefore the first and the last entry in the vector are treated like neighbours in any relevant group competition or migration event. At the end of each time step, we update the population vector depending on the changes that happened in that time step. Each simulation run starts with a population vector of zeros, except for the first entry, which is a 1. This represents a mutant cooperator in a population of defectors. Since the cycle we use in our model is a transitive graph (Taylor et al., 2007a), the location of the initial mutant does not matter.[3] As mentioned in Section 2, there are only two absorbing states in our model: one in which there are only cooperators and one with only defectors. We made sure that each individual simulation run presented in our results was long enough such that it reached either one of the two absorbing states.

We used random draws to decide what type of event – individual, group, or migration – happens in a given time step, and to determine the details of the corresponding event.[4] At each time step, we draw a random number from a uniform distribution between 0 and 1,[5] and,

- **if the random draw is lower than p, an individual event happens.** In this case, we draw a random integer between 1 and m to decide in which group the individual event will occur. Individual payoffs within the group are defined as presented in Section 2; cooperators within the group get a payoff of $1 - wc$, and defectors get a payoff of 1. Using these payoffs and the number of cooperators in the group that is chosen for the individual event, we define the probabilities with which the population state changes in this particular event as follows. Suppose that there are k_i cooperators in the group, then with probability p^+, the number of cooperators in the group increases by one – a cooperator is chosen to reproduce and a defector is chosen to die – and, with probability p^-, the number of cooperators decreases by one – a defector is chosen to reproduce and a cooperator is chosen to die – where

[3]We ran robustness checks where we placed the initial mutant at different locations on the population vector, and the results did not change significantly. Therefore, we decided to keep the location of the mutant fixed in the first group.

[4]In all of the random draws in our simulations, we used the *rand* function in Matlab. For the cases where we needed integers, we used the appropriate combination of our population size parameters and the *ceil* function in Matlab. For example, when we needed to choose a group randomly, we used the combination *ceil(rand*m)*, where *rand* draws a number from the uniform distribution on the interval $[0, 1]$, multiplying this number by m turns it into a random draw from the uniform distribution on the interval $[0, m]$, and *ceil* rounds the numbers up such that we end up with only integers between 1 and m, where each integer is equally likely to be drawn.

[5]The default precision of the *rand* function in Matlab is 4 decimal points, therefore, the probability of drawing a specific number is approximately zero. For our decisions on what type of events are happening in the simulations, it does not matter substantially where we place the cases of the random draws being equal to certain numbers, e.g. that the random draw is exactly equal to p.

$$p^+ = \frac{k_i(1-wc)}{n-k_iwc}\frac{n-k_i}{n}$$
$$p^- = \frac{n-k_i}{n-k_iwc}\frac{k_i}{n}$$

With the remaining probability $1-p^+-p^-$, the number of cooperators does not change, which would represent the case in which the reproducing and the dying individuals are both cooperators, or the case in which because they are both defectors. After defining the probabilities p^+ and p^- within the group, we draw another uniform random variable between 0 and 1 to decide which of the above events happens within the group. If the random number is lower than p^+, the number of cooperators in the group increases by one; if it is higher than or equal to p^+ but lower than $p^+ + p^-$, the number of cooperators in the group decreases by one; and, if it is higher than $p^+ + p^-$, the number of cooperators in the group does not change.

- **if the random draw is higher than or equal to p but lower than $\mathbf{p+q}$, a group event happens.** In this case, we define the group payoff for group i as $g(k_i) = 1 + w\frac{k_i}{n}b$, where k_i is the number of cooperators in group i at the beginning of the current time step. Then, based on our model, group i reproduces with probability $q(k_i, K)$, where

$$q(k_i, K) = \frac{g(k_i)}{\sum_{j=1}^{m} g(k_j)} = \frac{1 + w\frac{k_i}{n}b}{n + w\frac{K}{n}b}$$

  and $K = \sum_{j=1}^{m} k_j$ is the total number of cooperators in the population. Together, these give n thresholds; threshold j is $\sum_{i=1}^{j} q(k_i, K)$. Then we draw a uniform random number between 0 and 1, and if it is between threshold $j-1$ and j, group j is chosen to reproduce. Then, two additional uniform random numbers are drawn to choose the dying group and the location of the offspring group. In the BD process, one of the random draws is compared to $1/m$ to determine if the reproducing group is chosen to die or not. The other random draw is compared to 1/2 to decide whether the group above or below in the population vector is chosen to die, in case the reproducing group is not chosen to die. The population vector is updated such that the number of cooperators in the place of the dying group becomes equal to the number of cooperators in its reproducing neighbour. In the Shift process, one of the random draws is used to determine which of the groups from the whole population of groups is chosen to die, and the other random draw is compared to 1/2 to determine the direction in which the offspring group is placed in the population vector. Every entry between the reproducing group and the dying group in the vector is shifted by one spot in the chosen direction.

- **if the random draw is higher than or equal to $\mathbf{p+q}$, a migration event happens.** By drawing a random integer between 1 and m, we choose the first group to take part in the migration event. In our model, migration events occur between immediate neighbours. With the use of another random draw, we decide which neighbour of the first group is going to take part in the migration event as well. Within each group, a random individual is picked to migrate in our model, and, only in the case that the individuals migrating are of different types, i.e. one is a cooperator and the other is a defector, the population state changes. Making use of the number of cooperators given by the population vector, we

calculate the probabilities that the first group sends a cooperator or a defector, and the probabilities that the second group sends a cooperator or a defector. Then, we draw two random numbers to decide what type of player is sent by each group (this is done in a similar fashion to other type of choices mentioned above).

Depending on the type of the event, and what happens in a given event, the population vector at the end of each time step is updated as described. This procedure is repeated until we reach one of the absorbing states, or once the maximum time steps set is reached (in the latter case, we extend the maximum time steps and re-run the same setup until the run ends due to reaching fixation). At the end of each individual simulation run, we record whether cooperators or defectors fixated in that run.

This simulation program is used for finding critical b/c-ratio's for different choices of n, m, p, q, and r. For any given combination of n, m, p, q, and r, we do this by choosing $c = 0.1$, choosing a b, and running the simulation program a 1,000,000 times. This gives an estimate of the fixation probability, which we compare to $\frac{1}{nm}$, which is the fixation probability under neutral selection. The b is then adjusted accordingly, until we find a fixation probability that is indistinguishable from $\frac{1}{nm}$.

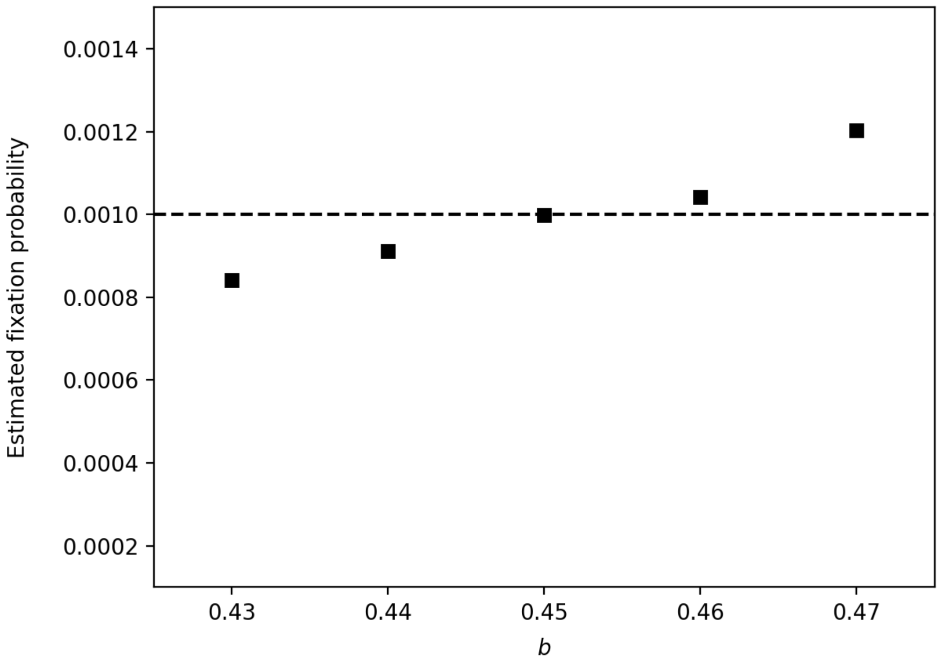

Figure 7: An example of the last few steps in the iterative process of finding the critical b/c-ratio. Here, $m = 50$ and $n = 20$, which makes the fixation probability in the neutral process $\frac{1}{1000}$. Furthermore, this is the Birth-Death process, $c = 0.1$, $w = 0.5$, $p = \frac{18}{21}$, $q = \frac{9}{210}$, and $r = \frac{1}{10}$. In this particular instance, the number of fixations for $b = 0.45$ was almost spot on (999 out of 1,000,000), so we settled on a b/c-ratio of 4.5. In other instances, we rounded to the nearest value for b with 2 digits precision. This procedure results in one critical b/c-ratio, and therefore one point in Fig. 9a.

7 Theoretical results and simulations

In this section, we take the critical b/c ratios in the limit of weak selection, calculated in Section 3, combine them, as we did in Section 5, with the relatednesses in the limit of weak selection, calculated in Section 4, and plot them for a variety of parameter combinations. We combine those analytical results in the limit of weak selection with simulation results not in the limit of weak selection. As we vary group size n, the number of groups m, and migration rate r, we want to choose the probabilities of group versus individual events such that the ratio of probabilities for an individual to die in an individual event and in a group event remains constant under neutral selection. In this case, we choose them so that these probabilities are always equally large. At neutrality, the probability that an individual dies in an individual event is $p\frac{1}{m}\frac{1}{n}$ and the probability that an individual dies in a group event is $q\frac{1}{m}$. Keeping them equal therefore requires

$$p\frac{1}{m}\frac{1}{n} = q\frac{1}{m}$$
$$p = nq$$

We also have the condition $p + q + r = 1$. Together with the equality above, this implies that

$$p + q = 1 - r$$
$$(n + 1)q = 1 - r$$
$$q = (1 - r)\frac{1}{n + 1} \rightarrow p = (1 - r)\frac{n}{n + 1}$$

In Fig. 8 and Fig. 9, we choose $r = 0.1$, and in Fig. 10, r varies.

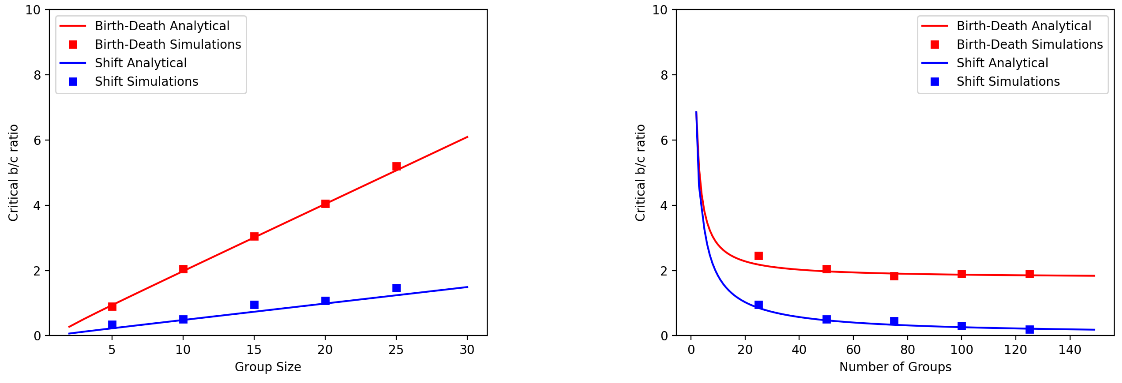

(a) $m = 50$ **(b)** $n = 10$

Figure 8: Results for the critical b/c ratios, combined with simulations with $1,000,000$ independent runs, where $w = 0.1$, $r = 0.1$, $p = 0.9\frac{n}{n+1}$ and $q = 0.9\frac{1}{n+1}$. In **(a)**, the effect of increasing the group size is shown for a fixed number of groups. In **(b)**, the effect of increasing the number of groups is shown for a fixed group size.

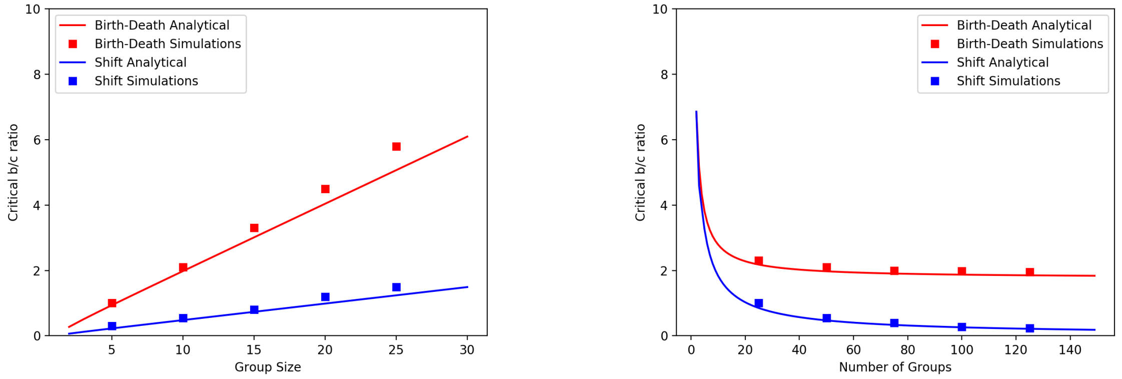

(a) $m = 50$ **(b)** $n = 10$

Figure 9: Same as Fig. 8, but with $w = 0.5$.

In Fig. 8a and Fig. 9a, we see that the thresholds in both processes increase with group size, and that the gap between the critical b/c ratios for the two processes is there for a range of group sizes. In Fig. 8b and Fig. 9b, we see that the thresholds decrease with the number of groups, and, again, that the gap between the critical b/c ratios for the two processes is there for a range of numbers of groups. Both the increase of the thresholds with group size and the decrease with the number of groups are in line with the results of Traulsen and Nowak (2006). The gap between the thresholds for the two processes in relative terms becomes (very) large for (very) large numbers of groups.

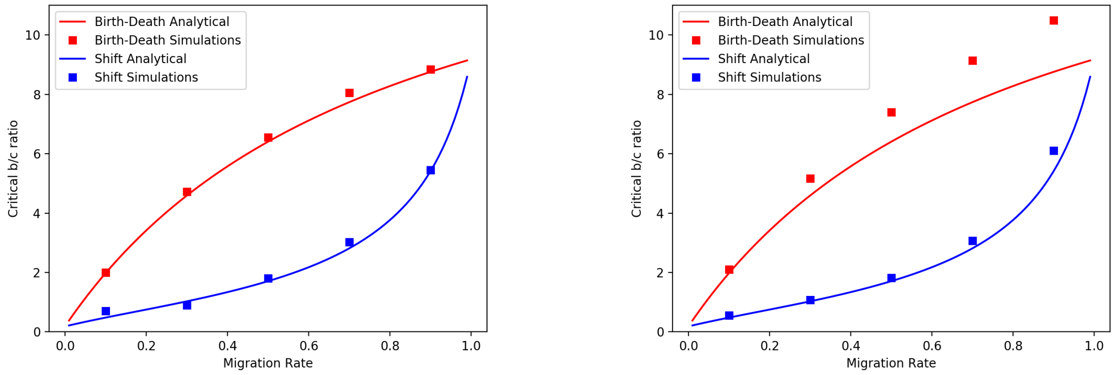

(a) $w = 0.1$ **(b)** $w = 0.5$

Figure 10: Results for the critical b/c ratios for different migration rates r, for $n = 10, m = 50$, again combined with simulations with $1,000,000$ independent runs.

In Fig. 10, we see that both thresholds increase with the migration rate. This is understandable, since a higher migration rate will result in lower relatedness. We also see that the gap disappears for migration rates close to 0 and migration rates close to 1. For migration rates close to 0, and not too few groups, there is a fair chance that the process will spend a lot of time in population states where all groups are at within-group fixation, or, in other words, where all groups consist of only cooperators or only defectors. Once this has happened, there is a fair chance that the population will get to a state where these all-cooperator groups

form one sequence of groups on the circle. Without mutation or migration, this will remain true from then onwards. In Section 8, we will see that if we imagine a mutant group, where all individuals are cooperators, the fixation probability of such a mutant group is the same in either process. If cooperators on average gain ground on (or lose ground to) defectors, they do so faster with Shift than with BD, but the condition under which they gain or loose is the same for both processes. The reason for the difference in speed is that with Shift, in all intermediate population states with a string of all-cooperator groups and a string of all-defector groups, any all-cooperator group has a real chance of replacing any all-defector group and vice versa. On the other hand, in BD, all the action is at the two boundaries between the strings of all-cooperator groups and all-defector groups, while group reproduction events not on the boundary are inconsequential for the population state. This implies that the boundary moves much more with Shift than it does with BD, and if the boundary moves in expectation in favour of the all-cooperator groups, it will move faster with Shift than with BD. However, the speed turns out not to matter for the fixation probability at the group level of a mutant all-cooperator group. The speed does matter if there are mixed groups, because if there are mixed groups, the ground gained at the group level will balance against the ground lost at the individual level. With cooperative groups winning faster in Shift, they can therefore overcome a larger within-group decay of cooperators. Absent mixed groups, however, speed does not matter anymore, and the difference in fixation probabilities disappears.

At migration rates r close to 1, it is not hard to understand why the difference between BD and Shift disappears as well. When almost all events are migration events, the population is shaken and stirred between any two reproduction events, be it at the individual or at the group level, and all that matters is what being a cooperator does to an individual's own birth rate and what it does to its group's birth rate. Those effects are the same in both processes, and therefore the difference between the two processes should disappear at very high migration rates.

This is also reflected in the formulas for the thresholds. In the limit of $r \uparrow 1$, only finite population effects remain, and relatednesses become $r_s = r_1 = -\frac{1}{nm-1}$ and $r_0 = \frac{1}{n} + \frac{n-1}{n} r_s = \frac{1}{n} - \frac{n-1}{n}\frac{1}{nm-1} = \frac{m-1}{nm-1}$. This implies that $1 - r_0 = \frac{(n-1)m}{nm-1}$ and $r_0 - r_1 = \frac{m}{nm-1}$.

Condition (2), the critical b/c-ratio for BD, then becomes

$$\frac{b}{c} > \frac{p}{q}\frac{m}{m-1}\frac{1-r_0}{n(r_0-r_1)} = \frac{p}{q}\frac{m}{m-1}\frac{n-1}{n}$$

Condition (4), the critical b/c-ratio for Shift, then becomes

$$\frac{b}{c} > \frac{p}{q}\frac{1-r_0}{nr_0} = \frac{p}{q}\frac{m}{m-1}\frac{n-1}{n}$$

These thresholds are the same.

8 Analytical solutions, without migration, and for limit cases that are not the limit of weak selection

There are some limit cases, other than the limit of weak selection, for which it also becomes feasible to derive analytical solutions for fixation probabilities. These results will help understand why the difference between the critical b/c ratios for BD and for Shift disappears when migration rates get close to 0. In both limits we consider here, we assume that there is no migration ($r = 0$), we assume strong selection ($w = 1$), and we assume that there is a separation of timescales. In the first limit, where $p \to 1$, group events are rare, and in almost all events, an individual reproduces. This limit is similar to the limit for which Traulsen and Nowak (2006) derive their formula [**1**]. They do consider the limit of weak selection, while we consider strong selection – which means that by scaling the b and c, we can really consider any intensity of selection. In the second limit, where $p \to 0$, individual events are rare, and in almost all events, a group reproduces.

8.1 Fixation probabilities of mutant groups

To compute fixation probabilities in both limits, it will be useful first to compute the fixation probability of a "mutant group", with l cooperators in it, in a population of groups, all of which contain k cooperators. This is the process at the group selection time scale[6]. It will only visit states in which there is one string of subsequent groups with k cooperators, and one string of subsequent groups with l cooperators – besides of course the absorbing states, where all groups have k cooperators or all groups have l cooperators. Therefore, the population state can be denoted by a single number i, $0 \leq i \leq m$, representing the number of groups with l cooperators.

Birth-Death

For the Birth-Death process, the only way in which i can change is if one of the groups on the two boundaries between the strings of different group types is chosen for reproduction; one of the two k-groups on the boundary if i is to go down, and one of the two l-groups on the boundary if i is to go up. Moreover, it needs to replace the group on the other side of the boundary, and not its other neighbour or itself, which happens with probability $\frac{1}{2}\frac{m-1}{m}$. The probability of a transition from i to $i-1$ in the Birth-Death process then becomes $T^{-}_{i,BD} = 2 \cdot \frac{1}{2}\frac{m-1}{m} \cdot \frac{1+\frac{k}{n}b}{i(1+\frac{l}{n}b)+(m-i)(1+\frac{k}{n}b)}$, while the probability of a transition from i to $i+1$ is $T^{+}_{i,BD} = 2 \cdot \frac{1}{2}\frac{m-1}{m} \cdot \frac{1+\frac{l}{n}b}{i(1+\frac{l}{n}b)+(m-i)(1+\frac{k}{n}b)}$. That makes the down-up ratio for the Birth-Death process equal to

$$\frac{T^{-}_{i,BD}}{T^{+}_{i,BD}} = \frac{1+\frac{k}{n}b}{1+\frac{l}{n}b}$$

[6] The fixation probabilities follow more or less directly from the fixation probabilities in the standard Birth-Death process on the cycle (Ohtsuki and Nowak, 2006; Nowak, 2006), or the standard Shift process (Allen and Nowak, 2012). The only difference is that in the standard versions, there are individuals on a cycle, and here we have groups on a cycle instead.

Shift

For the Shift process, change is much more likely. State i changes any time the group that is chosen for reproduction is of a different type than the group that is chosen for death. The probability of a transition from i to $i-1$ is $T^-_{i,Shift} = \frac{(m-i)(1+\frac{k}{n}b)}{i(1+\frac{l}{n}b)+(m-i)(1+\frac{k}{n}b)}\frac{i}{m-1}$, while the probability of a transition from i to $i+1$ is $T^+_{i,Shift} = \frac{i(1+\frac{l}{n}b)}{i(1+\frac{l}{n}b)+(m-i)(1+\frac{k}{n}b)}\frac{m-i}{m-1}$. That makes the down-up ratio for the Shift process also equal to

$$\frac{T^-_{i,Shift}}{T^+_{i,Shift}} = \frac{1+\frac{k}{n}b}{1+\frac{l}{n}b}$$

Birth-Death and Shift

Since the down-up ratios are the same for both, the fixation probability of a group with l cooperators, while all other groups have k of them, is also the same for Birth-Death and Shift:

$$\tau_{k\to l} = \frac{1}{1+\sum_{j=1}^{m-1}\prod_{i=1}^{j}\frac{1+\frac{k}{n}b}{1+\frac{l}{n}b}} = \frac{1}{1+\sum_{j=1}^{m-1}\left(\frac{1+\frac{k}{n}b}{1+\frac{l}{n}b}\right)^j} = \frac{1}{1+\frac{1-\left(\frac{1+\frac{k}{n}b}{1+\frac{l}{n}b}\right)^m}{1-\frac{1+\frac{k}{n}b}{1+\frac{l}{n}b}}-1} = \frac{1-\frac{1+\frac{k}{n}b}{1+\frac{l}{n}b}}{1-\left(\frac{1+\frac{k}{n}b}{1+\frac{l}{n}b}\right)^m}$$

This can be rewritten as

$$\tau_{k\to l} = \frac{\left(1+\frac{l}{n}b\right)-\left(1+\frac{k}{n}b\right)}{\left(1+\frac{l}{n}b\right)^m-\left(1+\frac{k}{n}b\right)^m}\frac{\left(1+\frac{l}{n}b\right)^m}{1+\frac{l}{n}b} = \frac{\frac{l-k}{n}b\left(1+\frac{l}{n}b\right)^{m-1}}{\left(1+\frac{l}{n}b\right)^m-\left(1+\frac{k}{n}b\right)^m}$$

In the limit of $p\to 1$, we will use $\tau_{0\to n}$ and $\tau_{n\to 0}$, and in the limit of $p\to 0$, we will use $\tau_{k\to k+1}$ and $\tau_{k\to k-1}$ for $1\le k\le m-1$.

8.2 The limit $p\to 1$

In this limit, group events are rare, while there are individual events almost all of the time. This implies that within her group, an initial mutant will either have gone extinct, or gone to fixation, before a group event happens. With the separation of timescales, we can first concentrate on the probabilities of fixation within the group happening. With j being the number of cooperators in the group, a defector replaces a cooperator within the group, and a cooperator replaces a defector, with probabilities

$$T^-_j = \frac{n-j}{n-jc}\frac{j}{n} \qquad \text{and} \qquad T^+_j = \frac{j(1-c)}{n-jc}\frac{n-j}{n}$$

The down-up ratio therefore is $\frac{T^-_j}{T^+_j} = \frac{1}{1-c}$, making the fixation probabilities within the group

$$\sigma_C = \frac{1-\frac{1}{1-c}}{1-\left(\frac{1}{1-c}\right)^n} = \frac{c(1-c)^{n-1}}{1-(1-c)^n} \qquad \text{and} \qquad \sigma_D = \left(\frac{1}{1-c}\right)^{n-1} \frac{1-\frac{1}{1-c}}{1-\left(\frac{1}{1-c}\right)^n} = \frac{c}{1-(1-c)^n}$$

After fixation within the group, all groups will be homogeneous. As soon as the population consists of homogeneous groups, the population state can only change with a group event. Therefore, the fixation probability of a mutant will be the product of the fixation probability of the mutant within the group – which is the same for both processes, because this does not involve group events – and the fixation probability of the all-mutant group in the population – which is also the same for both processes, as we have seen above.

$$\rho_C = \sigma_C \tau_{0\to n} = \frac{1-\frac{1}{1-c}}{1-\left(\frac{1}{1-c}\right)^n} \frac{1-\frac{1}{1+b}}{1-\left(\frac{1}{1+b}\right)^m} = \frac{c(1-c)^{n-1}}{1-(1-c)^n} \frac{b(1+b)^{m-1}}{(1+b)^m-1}$$

and

$$\rho_D = \sigma_D \tau_{n\to 0} = \frac{1-(1-c)}{1-(1-c)^n} \frac{1-(1+b)}{1-(1+b)^m} = \frac{c}{1-(1-c)^n} \frac{b}{(1+b)^m-1}$$

If we do consider the limit of weak selection, then we can approximate σ_C with $\frac{1}{n}\left(1-\frac{n-1}{2}c\right)$ for small c, and $\tau_{0\to n}$ with $\frac{1}{m}\left(1+\frac{m-1}{2}b\right)$ for small b. That implies that in the limit of weak selection, $\rho_C > \frac{1}{nm}$ if

$$\frac{b}{c} > \frac{n-1}{m-1}.$$

Condition **[1]** in Traulsen and Nowak (2006) is

$$\frac{b}{c} > 1 + \frac{n}{m}.$$

This condition applies to their model without migration, and also with almost all events being individual replacements. In their model, the individual reproduction rate in all-cooperators groups (and therefore also their group reproduction rate) is $b-c$ higher than the individual reproduction rate in all-defector groups. In our model, the group reproduction rate is increased by b, which explains why there is a 1 on the right hand side in their inequality, which one can also write as $\frac{b-c}{c} > \frac{n}{m}$, and not in ours.

Similarly, we can approximate σ_D with $\frac{1}{n}\left(1+\frac{n-1}{2}c\right)$ for small c, and $\tau_{n\to 0}$ with $\frac{1}{m}\left(1-\frac{m-1}{2}b\right)$ for small b. In the limit of weak selection, that gives the same threshold for when $\rho_D < \frac{1}{nm}$.

Traulsen and Nowak (2006) also consider a version with migration, but because the migration rate is taken to be proportional to the probability that an individual reproduction event induces the group to split (which is vanishingly small), this implies that these events are also only happening occasionally, and if they do, the migrants almost always either take over their new group or go extinct there before anything else happens that is not an individual event.

8.3 The limit $p \to 0$

In this limit, individual events are rare, while there are group events almost all of the time. The population will therefore regularly be in a state in which all groups have the same composition. When each group has the same number of cooperators, the population state can only change as the result of an individual event. If it does, then that event can make the number of cooperators within one group go up by one, or go down by one. After that, a sequence of group events either make the new "mutant group" go extinct, or go to fixation. Fixation of the mutant group happens with probability $\tau_{k\to k+1}$ if the individual event had a cooperator replace a defector, and with probability $\tau_{k\to k-1}$ if a defector replaced a cooperator. With rare individual events, the population, on the larger time scale, therefore moves between states where all groups have the same number of cooperators, and this number goes up or down by at most 1. The probability that it goes down by one is the probability with which a defector reproduces, which is $\frac{n-k}{n-kc}$, times the probability that a cooperator dies, which is $\frac{k}{n}$, times the fixation probability of the "mutant group", which is $\tau_{k\to k-1}$. The probability that it goes up by one is the probability with which a cooperator reproduces, which is $\frac{k(1-c)}{n-kc}$, times the probability that a defector dies, which is $\frac{n-k}{n}$, times the fixation probability of the "mutant group", which is $\tau_{k\to k+1}$. The down-up ratio at the larger timescale therefore is

$$\frac{\mathbf{T}_k^-}{\mathbf{T}_k^+} = \frac{1}{1-c}\frac{\tau_{k\to k-1}}{\tau_{k\to k+1}}$$

They can be smaller or larger than 1, depending on the parameters. With these down-up ratios at the larger timescale, we find the overall fixation probabilities of cooperators and defectors.

$$\lim_{p\to 0} \rho_C = \tau_{0\to 1}\frac{1}{1+\sum_{k=1}^{n-1}\prod_{l=1}^{k}\frac{\mathbf{T}_l^-}{\mathbf{T}_l^+}} = \tau_{0\to 1}\frac{1}{1+\sum_{k=1}^{n-1}\left(\frac{1}{1-c}\right)^k\prod_{l=1}^{k}\frac{\tau_{l\to l-1}}{\tau_{l\to l+1}}}$$

and

$$\lim_{p\to 0} \rho_D = \tau_{n\to n-1}\frac{\prod_{k=1}^{n-1}\frac{\mathbf{T}_k^-}{\mathbf{T}_k^+}}{1+\sum_{k=1}^{n-1}\prod_{l=1}^{k}\frac{\mathbf{T}_l^-}{\mathbf{T}_l^+}} = \tau_{n\to n-1}\frac{\left(\frac{1}{1-c}\right)^{n-1}\prod_{l=1}^{n-1}\frac{\tau_{l\to l-1}}{\tau_{l\to l+1}}}{1+\sum_{k=1}^{n-1}\left(\frac{1}{1-c}\right)^k\prod_{l=1}^{k}\frac{\tau_{l\to l-1}}{\tau_{l\to l+1}}}$$

The fixation probability ρ_C of a cooperator therefore is larger than the fixation probability ρ_D of a defector, if

$$\tau_{0\to 1} > \tau_{n\to n-1}\left(\frac{1}{1-c}\right)^{n-1}\prod_{l=1}^{n-1}\frac{\tau_{l\to l-1}}{\tau_{l\to l+1}},$$

or, in other words, if

$$1 > \left(\frac{1}{1-c}\right)^{n-1} \frac{\prod_{l=1}^{n} \tau_{l\to l-1}}{\prod_{l=0}^{n-1} \tau_{l\to l+1}}$$
$$1 > \frac{1}{(1-c)^{n-1}} \frac{1}{(1+b)^{m-1}}$$

In the limit of weak selection, this can be approximated with

$$1 > (1 + (n-1)c)\,(1 - (m-1)b)\,,$$

and, still in the limit of weak selection, this is true if

$$\frac{b}{c} > \frac{n-1}{m-1}.$$

The effect of the different separations of time scales therefore is the same in the limit of weak selection, and in both of them, the cancellation effect dissipates; in the first, because there are almost no mixed groups, in the second because the population almost never consists of differently composed groups. Note also that this formula makes perfect sense for $n = 1$, in which case any positive group benefit will make cooperators do better than defectors, and for $m = 1$, in which case no benefit is ever high enough to offset positive costs of cooperation.

9 Empirical implications

9.1 F_{ST}

Many empirical papers estimate F_{ST}'s. Our definition of r_s follows the definition of relatedness in Rousset (2004) and Durrett (2008), applied to members of the same group, and our definition of relatedness between members of different groups applies the same definition to individuals from different groups. Many empiricists however use other definitions. We assume that the definitions empiricists use are equivalent to the definition from Rousset (2004) and Durrett (2008), applied to members of the same group, but we have not found a reference where this is proven formally. What we will do below is to indicate how the two definitions used by most empiricists are the same as the probabilistic definition of relatedness within the group that we also used at the end of Section 4. There, relatedness between two individuals in the same group, for a given population state k, is defined as $r_{s,k} = \mathbb{P}_{s,k}(C|C) - \mathbb{P}_{s,k}(C|D)$, where the probabilities refer to the outcomes of two subsequent draws from the same group, without replacement. Relatedness r_s is then defined as the weighted average $r_s = \sum_k q_k r_{s,k}$ of those relatednesses over different states, where the weights q_k of states are given by the invariant distribution of the Markov chain in the limit of no mutation, normalized after excluding the absorbing states where all individuals are cooperators, or all are defectors (Allen and Tarnita, 2014).

Our model has groups of equal size, and let f_i be the fraction of groups with i cooperators out of n group members in population state k (we suppress the population state k in the notation). The overall frequency of cooperators and defectors follow from these fractions of group types: $\sum_{i=0}^{n} f_i \frac{i}{n} = p$ and $\sum_{i=0}^{n} f_i \frac{n-i}{n} = 1-p$. Also the fractions of group types must add up to 1; $\sum_{i=0}^{n} f_i = 1$. Then, we can rewrite $r_{s,k}$ as

$$
\begin{aligned}
r_{s,k} = \mathbb{P}_{s,k}(C|C) - \mathbb{P}_{s,k}(C|D) &= \frac{\sum_{i=0}^{n} f_i \frac{i}{n}\frac{i-1}{n-1}}{\sum_{i=0}^{n} f_i \frac{i}{n}} - \frac{\sum_{i=0}^{n} f_i \frac{n-i}{n}\frac{i}{n-1}}{\sum_{i=0}^{n} f_i \frac{n-i}{n}} = \frac{\sum_{i=0}^{n} f_i \frac{i}{n}\frac{i-1}{n-1}}{p} - \frac{\sum_{i=0}^{n} f_i \frac{n-i}{n}\frac{i}{n-1}}{1-p} \\
&= \frac{\sum_{i=0}^{n} f_i \frac{i}{n}\frac{i-1}{n-1}(1-p)}{p(1-p)} - \frac{\sum_{i=0}^{n} f_i \frac{n-i}{n}\frac{i}{n-1}p}{p(1-p)} \\
&= \frac{\sum_{i=0}^{n} f_i \frac{i}{n}\left(1-\frac{n-i}{n-1}\right)(1-p)}{p(1-p)} - \frac{\sum_{i=0}^{n} f_i \frac{n-i}{n}\frac{i}{n-1}p}{p(1-p)} \\
&= \frac{\sum_{i=0}^{n} f_i \frac{i}{n}(1-p) - \sum_{i=0}^{n} f_i \frac{i}{n}\frac{n-i}{n-1} + \sum_{i=0}^{n} f_i \frac{i}{n}\frac{n-i}{n-1}p}{p(1-p)} - \frac{\sum_{i=0}^{n} f_i \frac{n-i}{n}\frac{i}{n-1}p}{p(1-p)} \\
&= \frac{p(1-p) - \sum_{i=0}^{n} f_i \frac{i}{n}\frac{n-i}{n-1}}{p(1-p)}
\end{aligned}
$$

The final expression matches the way F_{ST} is regularly defined (see Equation 50), except for one detail. With groups numbered from $j = 1, ..., m$, the normal definition would have $\sum_j^m p_j(1-p_j)$ as a second term in the numerator, where $p_j(1-p_j)$ is the variance within group j, and $p(1-p)$ is the variance in the population as a whole. In our case, with f_i counting the numbers of groups with i cooperators out of n group members, this average within group variance would be $\sum_{i=0}^{n} f_i \frac{i}{n}\frac{n-i}{n}$. In our formula, we have $\sum_{i=0}^{n} f_i \frac{i}{n}\frac{n-i}{n-1}$ instead.

Here it is worth noting that the variance is half the expected squared difference between two independent draws from the same distribution. If X_1 and X_2 are two independent draws for the same random variable (which therefore have the same first and second moment), then the expected value of the squared difference

between them is

$$\mathbb{E}\left[X_1 - X_2\right]^2 = \mathbb{E}\left[X_1^2 - X_2^2 - 2X_1X_2\right] = 2\mathbb{E}\left[X_1^2\right] - \mathbb{E}^2\left[X_1\right] = 2Var(X_1)$$

In our case, X_1 and X_2 represent drawing an individual from one and the same group, where drawing a cooperator makes the value of X to be 1, and drawing a defector makes it 0. If we draw two individuals without replacement, instead of with replacement, then they are no longer independent. We can however still compute the expected squared difference between the draws. This is

$$\frac{i}{n}\frac{i-1}{n-1} \cdot 0^2 + \frac{i}{n}\frac{n-i}{n-1} \cdot 1^2 + \frac{n-i}{n}\frac{i}{n-1} \cdot 1^2 + \frac{n-i}{n}\frac{n-i-1}{n-1} \cdot 0^2 = 2\frac{i(n-i)}{n(n-1)}$$

Half of this is what is used instead of the within-group variance. For every state k, the "without-replacement" version of the F_{ST} is therefore equivalent to the definition as a difference in conditional probabilities $r_{s,k} = \mathbb{P}_{s,k}\left(C|C\right) - \mathbb{P}_{s,k}\left(C|D\right)$. For large group sizes n, the difference between the with and without replacement version of the within-group variance disappears.

$$F_{ST} = \frac{\text{population variance} - \text{avergage within group variance}}{\text{population variance}} \tag{50}$$

This definition of the F_{ST} features both the average within-group variance $\sum_{i=0}^{n} f_i \frac{i}{n}\frac{n-i}{n-1}$ (with replacement) and the overall variance. There is yet another definition of F_{ST}, which expresses it in terms of within-group and between-group variance. We can take the following steps to get to that expression.

$$\begin{aligned}
r_{s,k} &= \frac{p(1-p) - \sum_{i=0}^{n} f_i \frac{i}{n}\frac{n-i}{n-1}}{p(1-p)} \\
&= \frac{p - \sum_{i=0}^{n} f_i \frac{i}{n}\frac{n-i}{n-1} - p^2}{p - \sum_{i=0}^{n} f_i \frac{i}{n}\frac{i-1}{n-1} + \sum_{i=0}^{n} f_i \frac{i}{n}\frac{i-1}{n-1} - p^2} \\
&= \frac{\sum_{i=0}^{n} f_i \frac{i}{n} - \sum_{i=0}^{n} f_i \frac{i}{n}\frac{n-i}{n-1} - p^2}{\sum_{i=0}^{n} f_i \frac{i}{n} - \sum_{i=0}^{n} f_i \frac{i}{n}\frac{i-1}{n-1} + \sum_{i=0}^{n} f_i \frac{i}{n}\frac{i-1}{n-1} - p^2} \\
&= \frac{\sum_{i=0}^{n} f_i \frac{i}{n}\left(1 - \frac{n-i}{n-1}\right) - p^2}{\sum_{i=0}^{n} f_i \frac{i}{n}\left(1 - \frac{i-1}{n-1}\right) + \sum_{i=0}^{n} f_i \frac{i}{n}\frac{i-1}{n-1} - p^2} \\
&= \frac{\sum_{i=0}^{n} f_i \frac{i}{n}\frac{i-1}{n-1} - p^2}{\sum_{i=0}^{n} f_i \frac{i}{n}\frac{n-i}{n-1} \quad + \quad \sum_{i=0}^{n} f_i \frac{i}{n}\frac{i-1}{n-1} - p^2}
\end{aligned}$$

Here $\sum_{i=0}^{n} f_i \frac{i}{n}\frac{n-i}{n-1}$ is the without replacement version of within-group variance as before. If we call $\sum_{i=0}^{n} f_i \frac{i}{n}\frac{i-1}{n-1} - p^2 = \sum_{i=0}^{n} f_i \frac{i}{n}\frac{i-1}{n-1} - \left(\sum_{i=0}^{n} f_i \frac{i}{n}\right)^2$ the without replacement version of between-group variance, we would read this as

$$F_{ST} = \frac{\text{between-group variance}}{\text{average within-group variance} + \text{between-group variance}} \quad (51)$$

Again, for large group sizes n, the with or without replacement versions are close.

9.2 Empirical estimates

The condition for cooperation to be selected for by group selection used in, for instance, Bell et al. (2009) (see also Weir and Cockerham 1984; Crow and Aoki 1984; Aoki and Nozawa 1984; Bowles 2006; 2009; Langergraber et al. 2011; Walker 2014; Rusch 2018) is

$$\frac{\beta(w_g, p_g)}{\beta(w_{ig}, p_{ig})} > \frac{1 - F_{ST}}{F_{ST}}$$

Here $\beta(w_g, p_g)$ is the increase in the mean fitness of the group as a result of an increase in the frequency of cooperators, or altruists, and $\beta(w_{ig}, p_{ig})$ is the decrease in fitness of an individual as a result of switching from defection to cooperation. The idea is that this criterion separates the fitness effects, on the left hand side of the inequality, from a measure that characterizes the population structure, on the right hand side of the inequality. In a setting with a linear public goods game, played within groups that compete with each other in a well mixed population of groups, such a separation can indeed be made in this way.

Suppose the fitness of a cooperator and a defector in a group with i cooperators (for cooperators: including the individual itself) are $w_{C,i} = 1 + \frac{i-1}{n-1}b - c$ and $w_{D,i} = 1 + \frac{i}{n-1}b$. Being a cooperator instead of a defector would then give $n-1$ others in the group a fitness benefit of $\frac{1}{n-1}b$, adding up to an aggregate fitness benefit of b, at a fitness cost to the individual of c. The average fitness within the group would go from $\frac{iw_{C,i}+(n-i)w_{D,i}}{n} = 1 + \frac{i}{n}(b-c)$ to $\frac{(i+1)w_{C,i+1}+(n-i-1)w_{D,i+1}}{n} = 1 + \frac{i+1}{n}(b-c)$, which amounts to an increase of $\frac{1}{n}(b-c)$ as a result of an increase in the frequency of cooperators within that group of $\frac{1}{n}$. This makes $\beta(w_g, p_g) = b - c$. The fitness effect measured by $\beta(w_{ig}, p_{ig})$ is similarly interpreted as c. Rewriting $\frac{b-c}{c} > \frac{1-F_{ST}}{F_{ST}}$ as $(b-c)F_{ST} > c(1-F_{ST})$ and then as $F_{ST}b > c$ gives Hamilton's rule, with $r = F_{ST}$.

If we were to measure $\beta(w_g, p_g)$ in a setting in which competition between groups is not actually global, but to some degree local, then the resulting value for $\beta(w_g, p_g)$ would not only reflect the effect of cooperators on the average fitness within the group, but a mixture of these fitness effects and the cancellation effect. A moderate value for $\beta(w_g, p_g)$ can both be the result of a moderate group benefit and the absence of the cancellation effect, and a high group benefit combined with the cancellation effect at the group level. In the latter case, the negative effect of having neighbouring groups with many cooperators, combined with the positive correlation between being a cooperative group and having neighbouring groups with many cooperators, would bias the estimated effect of – all else equal – the number of cooperators on average fitness within the group downwards. In other words, this term would end up absorbing the cancellation effect. In order to disentangle all fitness effects and the cancellation effect, one would have to estimate a more complex statistical model, which would not only use the composition of the own group as explanatory variable of the average fitness within the group, but also include the composition of neighbouring groups as an explanatory variable. This would be hard to estimate because it will require sufficiently high independent variation to overcome multicollinearity, but, if

successful, it would separate the positive effect of the cooperation within the group from the negative effect of having a cooperative neighbouring group.

What most empirical papers do, however, is only estimate the F_{ST}, which is then taken as an indication of how conducive the population structure is to cooperation. We have seen that the absence or presence of the cancellation effect – which is part of the population structure – can however make a huge difference for how much the group needs to benefit from cooperators in it, relative to the individual costs, in order for cooperation to spread in the population.

References

Benjamin Allen and Alex McAvoy. A mathematical formalism for natural selection with arbitrary spatial and genetic structure. *arXiv:1806.04717 [q-bio.PE]*, 2018.

Benjamin Allen and Martin A. Nowak. Evolutionary shift dynamics on a cycle. *Journal of Theoretical Biology*, 311:28–39, 2012.

Benjamin Allen and Corina E. Tarnita. Measures of success in a class of evolutionary models with fixed population size and structure. *Journal of Mathematical Biology*, 68:109–143, 2014.

Benjamin Allen, Arne Traulsen, Corina E. Tarnita, and Martin A. Nowak. How mutation affects evolutionary games on graphs. *Journal of Theoretical Biology*, 299:97–105, 2012.

Kenichi Aoki and Ken Nozawa. Average coefficient of relationship within troops of the japanese monkey and other primate species with reference to the possibility of group selection. *Primates*, 25(2):171–184, 1984.

Adrian V. Bell, Peter J. Richerson, and Richard McElreath. Culture rather than genes provides greater scope for the evolution of large-scale human prosociality. *Proceedings of the National Academy of Sciences*, 106 (42):17671–17674, 2009.

Samuel Bowles. Group competition, reproductive leveling, and the evolution of human altruism. *Science*, 314(5805):1569–1572, 2006.

Samuel Bowles. Did warfare among ancestral hunter-gatherers affect the evolution of human social behaviors? *Science*, 324(5932):1293–1298, 2009.

Rob Boyd. Density-dependent mortality and the evolution of social interactions. *Animal Behaviour*, 30: 972–982, 1982.

James F. Crow and Kenichi Aoki. Group selection for a polygenic behavioral trait: estimating the degree of population subdivision. *Proceedings of the National Academy of Sciences*, 81(19):6073–6077, 1984.

Richard Durrett. *Probability models for DNA sequence evolution. Probability and its applications, 2nd Edition.* Springer, New York, 2008.

Alan Grafen. Natural selection, kin selection and group selection. In J.R. Kreps and N.B. Davies, editors, *Behavioural Ecology, 2nd edn.*, pages 62–84. Blackwell, 1983.

Alan Grafen. An inclusive fitness analysis of altruism on a cyclical network. *Journal of Evolutionary Biology*, 20(6):2278–2283, 2007.

William D. Hamilton. The genetical evolution of social behaviour. I. *Journal of Theoretical Biology*, 7(1): 1–16, 1964a.

William D. Hamilton. The genetical evolution of social behaviour. II. *Journal of Theoretical Biology*, 7(1): 17–52, 1964b.

William D Hamilton. Selection of selfish and altruistic behaviour in some extreme models. In J.F. Eisenberg and W.S. Dillon, editors, *Man and beast: comparative social behaviour*. 1971.

Kevin Langergraber, Grit Schubert, Carolyn Rowney, Richard Wrangham, Zinta Zommers, and Linda Vigilant. Genetic differentiation and the evolution of cooperation in chimpanzees and humans. *Proceedings of the Royal Society B: Biological Sciences*, 278(1717):2546–2552, 2011.

Shishi Luo. A unifying framework reveals key properties of multilevel selection. *Journal of Theoretical Biology*, 341:41–52, 2014.

Shishi Luo and Jonathan C Mattingly. Scaling limits of a model for selection at two scales. *Nonlinearity*, 30 (4):1682, 2017.

Gustave Malécot. *Les Mathématiques de l'Hérédite.* Masson et Cie, Paris, 1948.

Martin A. Nowak. *Evolutionary dynamics.* Harvard University Press, 2006.

Martin A. Nowak, Corina E. Tarnita, and Edward O. Wilson. The evolution of eusociality. *Nature*, 466 (7310):1057, 2010.

Hisashi Ohtsuki. Does synergy rescue the evolution of cooperation? an analysis for homogeneous populations with non-overlapping generations. *Journal of Theoretical Biology*, 307:20–28, 2012.

Hisashi Ohtsuki and Martin A. Nowak. Evolutionary games on cycles. *Proceedings of the Royal Society of London B: Biological Sciences*, 273(1598):2249–2256, 2006.

Hisashi Ohtsuki, Christoph Hauert, Erez Lieberman, and Martin A. Nowak. A simple rule for the evolution of cooperation on graphs and social networks. *Nature*, 441(7092):502, 2006.

David C. Queller. Does population viscosity promote kin selection? *Trends in Ecology & Evolution*, 7(10): 322–324, 1992.

David C. Queller. Genetic relatedness in viscous populations. *Evolutionary Ecology*, 8(1):70–73, 1994.

Peter Richerson, Ryan Baldini, Adrian V Bell, Kathryn Demps, Karl Frost, Vicken Hillis, Sarah Mathew, Emily K Newton, Nicole Naar, Lesley Newson, et al. Cultural group selection plays an essential role in explaining human cooperation: A sketch of the evidence. *Behavioral and Brain Sciences*, 39, 2016.

François Rousset. *Genetic structure and selection in subdivided populations.* Princeton University Press, Princeton, NJ, 2004.

Hannes Rusch. Ancestral kinship patterns substantially reduce the negative effect of increasing group size on incentives for public goods provision. *Journal of Economic Psychology*, 64:105–115, 2018.

Burton Simon. A dynamical model of two-level selection. *Evolutionary Ecology Research*, 12(5):555–588, 2010.

Burton Simon, Jeffrey A. Fletcher, and Michael Doebeli. Towards a general theory of group selection. *Evolution*, 67:1561–1572, 2013.

Elliott Sober and David Sloan Wilson. *Unto others: the evolution and psychology of unselfish behavior.* Cambridge, MA: Harvard University Press, 1998.

Peter D. Taylor. Altruism in viscous populations—an inclusive fitness model. *Evolutionary Ecology*, 6(4): 352–356, 1992a.

Peter D. Taylor. Inclusive fitness in a homogeneous environment. In *Proc. R. Soc. Lond. B*, volume 249, pages 299–302. The Royal Society, 1992b.

Peter D. Taylor, Troy Day, and Geoff Wild. Evolution of cooperation in a finite homogeneous graph. *Nature*, 447(7143):469–472, 2007a.

Peter D. Taylor, Troy Day, and Geoff Wild. From inclusive fitness to fixation probability in homogeneous structured populations. *Journal of Theoretical Biology*, 249(1):101–110, 2007b.

Arne Traulsen and Martin A. Nowak. Evolution of cooperation by multilevel selection. *Proceedings of the National Academy of Sciences*, 103(29):10952–10955, 2006.

Matthijs van Veelen. Can Hamilton's rule be violated? *eLife*, 7:e41901, 2018.

Matthijs van Veelen, Shishi Luo, and Burton Simon. A simple model of group selection that cannot be analyzed with inclusive fitness. *Journal of Theoretical Biology*, 360:279–289, 2014.

Matthijs van Veelen, Benjamin Allen, Moshe Hoffman, Burton Simon, and Carl Veller. Hamilton's rule. *Journal of Theoretical Biology*, 414:176–230, 2017.

Robert S. Walker. Amazonian horticulturalists live in larger, more related groups than hunter–gatherers. *Evolution and Human Behavior*, 35(5):384–388, 2014.

Bruce S. Weir and C. Clark Cockerham. Estimating F-statistics for the analysis of population structure. *Evolution*, 38(6):1358–1370, 1984.

Stuart A. West, Ido Pen, and Ashleigh S. Griffin. Cooperation and competition between relatives. *Science*, 296(5565):72–75, 2002.

David Sloan Wilson and Edward Osborne Wilson. Rethinking the theoretical foundations of socio-biology. *Quarterly Review of Biology*, 82:327–348, 2007.

David Sloan Wilson, Gregory B. Pollock, and Lee A. Dugatkin. Can altruism evolve in purely viscous populations? *Evolutionary Ecology*, 6(4):331–341, 1992.
